# Effect of True and Sham Acupuncture on Radiation-Induced Xerostomia Among Patients With Head and Neck Cancer: A Randomized Clinical Trial

**DOI:** 10.1001/jamanetworkopen.2019.16910

**Published:** 2019-12-06

**Authors:** M. Kay Garcia, Zhiqiang Meng, David I. Rosenthal, Yehua Shen, Mark Chambers, Peiying Yang, Qi Wei, Chaosu Hu, Caijun Wu, Wenying Bei, Sarah Prinsloo, Joseph Chiang, Gabriel Lopez, Lorenzo Cohen

**Affiliations:** 1 Department of Palliative, Rehabilitation, and Integrative Medicine, University of Texas MD Anderson Cancer Center, Houston; 2 Department of Integrative Oncology, Fudan University Shanghai Cancer Center, Shanghai, China; 3 Department of Radiation Oncology, University of Texas MD Anderson Cancer Center, Houston; 4 Department of Dental Oncology, University of Texas MD Anderson Cancer Center, Houston; 5 Department of Radiation, Fudan University Shanghai Cancer Center, Shanghai, China; 6 Department of Anesthesiology, University of Texas MD Anderson Cancer Center, Houston

## Abstract

**Importance:**

Radiation-induced xerostomia (RIX) is a common, often debilitating, adverse effect of radiation therapy among patients with head and neck cancer. Quality of life can be severely affected, and current treatments have limited benefit.

**Objective:**

To determine if acupuncture can prevent RIX in patients with head and neck cancer undergoing radiation therapy.

**Design, Setting, and Participants:**

This 2-center, phase 3, randomized clinical trial compared a standard care control (SCC) with true acupuncture (TA) and sham acupuncture (SA) among patients with oropharyngeal or nasopharyngeal carcinoma who were undergoing radiation therapy in comprehensive cancer centers in the United States and China. Patients were enrolled between December 16, 2011, and July 7, 2015. Final follow-up was August 15, 2016. Analyses were conducted February 1 through 28, 2019.

**Intervention:**

Either TA or SA using a validated acupuncture placebo device was performed 3 times per week during a 6- to 7-week course of radiation therapy.

**Main Outcomes and Measures:**

The primary end point was RIX, as determined by the Xerostomia Questionnaire in which a higher score indicates worse RIX, for combined institutions 1 year after radiation therapy ended. Secondary outcomes included incidence of clinically significant xerostomia (score >30), salivary flow, quality of life, salivary constituents, and role of baseline expectancy related to acupuncture on outcomes.

**Results:**

Of 399 patients randomized, 339 were included in the final analysis (mean [SD] age, 51.3 [11.7] years; age range, 21-79 years; 258 [77.6%] men), including 112 patients in the TA group, 115 patients in the SA group, and 112 patients in the SCC group. For the primary aim, the adjusted least square mean (SD) xerostomia score in the TA group (26.6 [17.7]) was significantly lower than in the SCC group (34.8 [18.7]) (*P* = .001; effect size = −0.44) and marginally lower but not statistically significant different from the SA group (31.3 [18.6]) (*P* = .06; effect size = −0.26). Incidence of clinically significant xerostomia 1 year after radiation therapy ended followed a similar pattern, with 38 patients in the TA group (34.6%), 54 patients in the SA group (47.8%), and 60 patients in the SCC group (55.1%) experiencing clinically significant xerostomia (*P* = .009). Post hoc comparisons revealed a significant difference between the TA and SCC groups at both institutions, but TA was significantly different from SA only at Fudan University Cancer Center, Shanghai, China (estimated difference [SE]: TA vs SCC, −9.9 [2.5]; *P* < .001; SA vs SCC, −1.7 [2.5]; *P* = .50; TA vs SA, −8.2 [2.5]; *P* = .001), and SA was significantly different from SCC only at the University of Texas MD Anderson Cancer Center, Houston, Texas (estimated difference [SE]: TA vs SCC, −8.1 [3.4]; *P* = .016; SA vs SCC, −10.5 [3.3]; *P* = .002; TA vs SA, 2.4 [3.2]; *P* = .45).

**Conclusions and Relevance:**

This randomized clinical trial found that TA resulted in significantly fewer and less severe RIX symptoms 1 year after treatment vs SCC. However, further studies are needed to confirm clinical relevance and generalizability of this finding and to evaluate inconsistencies in response to sham acupuncture between patients in the United States and China.

**Trial Registration:**

ClinicalTrials.gov identifier: NCT01266044

## Key Points

**Question:**

Can acupuncture prevent radiation-induced xerostomia, an adverse effect among patients with head and neck cancer undergoing radiation therapy?

**Findings:**

In this randomized clinical trial with 339 participants, 12 months after the end of radiation therapy, the xerostomia score of the true acupuncture group was significantly lower than that of the standard care control group.

**Meaning:**

These findings suggest that acupuncture should be considered for the prevention of radiation-induced xerostomia, but further studies are needed to confirm their clinical relevance and generalizability.

## Teaser

This randomized clinical trial examines the efficacy of true or sham acupuncture vs standard care in the treatment of radiation-induced xerostomia among patients in the United States and China.

## Introduction

Salivary glands are markedly sensitive to radiation therapy, and damage is generally irreversible at doses higher than 50 Gy (to convert to rad, multiply by 100).^1^ By the end of treatment, more than 50% of patients who undergo radiation therapy involving major salivary glands experience the perception of hyposalivation, termed *radiation-induced xerostomia* (RIX).^1,2,3^ Dental complications, dysgeusia, dysphagia, odynophagia, and difficulty sleeping and speaking affect quality of life and are often associated with RIX.^4^ Despite some success with cytoprotection (eg, amifostine)^5^ and physical techniques designed to reduce salivary gland exposure during the delivery of radiation therapy,^6^ acute and chronic RIX still occurs,^7^ and there is no reliable method to treat established RIX, to our knowledge.^8^

The biological mechanisms by which acupuncture treatment affects xerostomia are not well understood, but in 1993, a study by Blom et al^9^ suggested that tissues surrounding the parotid glands experienced a significant increase in local blood flux after acupuncture. Several small studies have since shown acupuncture may reduce xerostomia symptoms.^10,11,12,13^ One study by Blom and Lundeberg^14^ found that in some patients, as few as 5 acupuncture treatments were associated with symptom relief for up to 3 years. Two pilot randomized clinical trials from our group^12,15^ reported that acupuncture could prevent RIX when given concurrently with radiation therapy.

This phase 3, randomized, sham-controlled, patient- and assessor-blinded clinical trial was designed to determine whether true acupuncture (TA), compared with sham acupuncture (SA) or a standard care control (SCC) and given concurrently with a 6- to 7-week course of radiation therapy, reduces the incidence or severity of RIX among patients with head and neck cancer. We also sought to explore whether the effects of acupuncture differed by treatment site (ie, United States vs China).

## Methods

**Figure 1.**
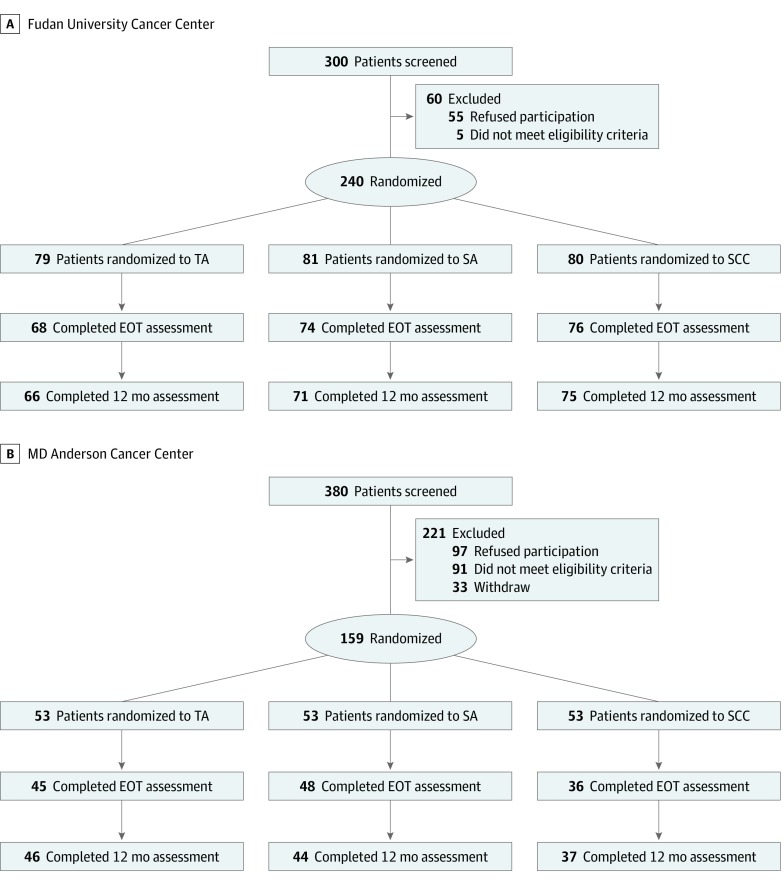
CONSORT Study Flow Diagram by Institution A, Among randomized patients at Fudan Cancer Center, 22 patients dropped out before the 12-month follow-up, including 17 who were unable to accommodate treatment requirements or scheduling, 4 who did not want to be in the SCC group, and 1 who felt acupuncture was too painful. B, Among patients at MD Anderson Cancer Center, 28 randomized patients dropped out before the 12 month follow-up, including 9 who withdrew after randomization into SCC, 8 unable to accommodate treatment requirements or scheduling, 6 who died, 2 missing baselines data for the primary end point, 2 who dropped out after beginning anticoagulant therapy, and 1 who felt acupuncture was too painful. A further 2 patients did not complete the end-of-treatment (EOT) assessment but did complete the 12-month assessment. SA indicates sham acupuncture; SCC, standard care control; and TA, true acupuncture.

This study was approved by the institutional review boards of the University of Texas MD Anderson Cancer Center in Houston, Texas (hereafter, *MD Anderson*) and Fudan University Cancer Center in Shanghai, China (hereafter, *Fudan*). Between December 16, 2011, and July 7, 2015, participants were identified by faculty in the radiation oncology departments at both institutions and referred to study personnel for assessment of eligibility (Figure 1). All study details were reviewed with eligible patients and oral and written informed consent were obtained from interested participants. Patients were compensated for their time at each assessment (US$35; ¥100 [US $14.15]). The study followed all Consolidated Standards of Reporting Trials (CONSORT) reporting guidelines, including details on recruitment and retention, study assignment, follow-up details, and information on final analyzed sample. The trial also followed the Standards for Reporting Interventions in Controlled Trials of Acupuncture (STRICTA) guidelines. The original trial protocol, original statistical analysis plan, summary of protocol revisions, summary of statistical analysis revisions, final trial protocol, and final statistical analysis plan are available in Supplement 1.

### Study Design

Using a centralized computer system, patients were randomized to 1 of 3 groups: TA, SA, or SCC. Patients were randomized separately at each institution by adaptive randomization (minimization). To ensure equal distribution of all factors across all groups and balance at each site, patients were stratified by stage of disease, age (running mean), sex, the mean planned parotid dose (left and right calculated separately and balanced between groups, <10, 10 to <20, 20 to <26, 26 to <30, 30 to <35, 35 to <40, 40 to <50, 50 to <60, or ≥60 Gy), induction therapy (yes or no), and concurrent chemotherapy (yes or no). Patients assigned to either TA or SA received acupuncture 3 days per week (same day as radiation treatment) during a 6- to 7-week course of radiation therapy. Patients in the SCC group received standard care, which included information about oral hygiene (brushing with fluoride toothpaste, flossing, and daily use of fluoride tray applications).

Patient-reported Xerostomia Questionnaires (XQs) and sialometry data were collected at baseline, at the end of radiation therapy (week 7), and 3, 6, and 12 months after the end of radiation therapy. Adverse events were recorded on each visit regardless of whether they were associated with acupuncture treatment.

### Study Population

Regardless of age, sex, or race/ethnicity, patients were eligible if they had received a diagnosis of head and neck squamous cell carcinoma (primarily oropharyngeal or nasopharyngeal) and were scheduled to undergo intensity-modulated radiation therapy with image guidance, with or without concurrent chemotherapy, at a mean dose of at least 24 Gy to at least 1 of the parotid glands (the other gland could receive any dose level). At Fudan, participants underwent radiation therapy as inpatients. At MD Anderson, patients underwent radiation therapy as outpatients. All patients were acupuncture naive. Full eligibility criteria and radiation therapy procedures are presented in eTable 1 and eTable 2 in Supplement 2.

### Acupuncture Treatment

Treatments were given by 6 qualified, hospital-credentialed acupuncturists with a mean (range) of 10 (5-25) years of experience. Quality control was maintained by having members of the study team, including acupuncturists, from MD Anderson visit Fudan, and vice versa, approximately every 6 months during the study period. Patients were treated in a comfortable supine or semisupine position on the day of radiation therapy, either before or after radiation therapy. Upon insertion, needles were manipulated until the de qi sensation was elicited at the appropriate points. They were not manipulated further during the needle retention period. The specific acupuncture points and needling methods used are reported in detail elsewhere^12,15^ and are provided in eTable 3 in Supplement 2. Patients were told that the purpose of the study was to test 2 different acupuncture approaches but that 1 approach might not target their dry mouth symptoms. This language was used to avoid deception while maintaining naiveté as to the existence of a sham group. The sham procedure in this randomized clinical trial involved a real needle at a real point not indicated for xerostomia, real needles at sham points, and placebo needles at sham points. The mixture of real and sham points and needles used is defined as acupuncture. The Park system, a validated, nonpenetrating, telescoping needle with a separate device that attaches it to the skin, was used for the placebo needles.^16,17^

### Measures

The primary aim of this study was to compare patient-reported outcome scores for xerostomia among the TA, SA, and SCC groups using the validated XQ. Because the subjective sensation of dry mouth is not highly correlated with the actual saliva flow rate, the US Food and Drug Administration requires patient-reported outcome scores for assessing xerostomia interventions. The XQ consists of 8 items scored on an 11-point scale of 0 to 10, has been validated in several cohorts, and is regarded as the criterion standard for measuring xerostomia.^1,2^ The sum of the item scores is transformed linearly to produce a final summary score between 0 and 100. Higher scores represent more xerostomia. Studies by Eisbruch et al^1^ and Pacholke et al^2^ suggest that an XQ score of 30 or less corresponds to mild to no symptoms of xerostomia, and a 10-point difference in score is considered clinically significant.

The Acupuncture Expectancy Scale^18^ was used to evaluate the association of baseline expectations related to acupuncture with clinical response. The Acupuncture Expectancy Scale is a 4-item instrument shown to be reliable (Cronbach α = 0.82) and valid by positive correlation with patient self-reported efficacy and satisfaction, with higher scores indicating higher expectations (range, 0-16). The scale has been further validated among patients with cancer who were acupuncture naive. All participants completed the Acupuncture Expectancy Scale at baseline. Patients in the 2 acupuncture groups also completed the form during the middle and at the end of radiation therapy.

Adverse events were recorded using Common Terminology Criteria for Adverse Events (CTCAE) version 3.0. Events that occurred during acupuncture treatment sessions and between treatment sessions were recorded regardless of whether they were related to acupuncture.

### Primary End Point

The primary end point was to determine whether TA was more effective than SA or SCC for reducing the severity and incidence of RIX among patients with cancer at MD Anderson and Fudan 1 year after the end of radiation therapy. Alpha for significance was adjusted to *P* = .025 to account for the 2 primary tests (TA vs SA and TA vs SCC at the 12-month follow-up). Other analyses are considered exploratory and should be interpreted with caution.

### Statistical Analysis

With 100 patients per group, and assuming a 2-sided significance level of .05, we had 80% power to detect an effect size of 0.4 between any pair of groups across the 3 postintervention time points (3, 6, and 12 months after radiation therapy). Per our preliminary data,^13,14^ we found differences on postintervention XQ means ranging from 0.80 to 0.68 SDs at the end of radiation therapy. We did not know the exact differences that would occur between the sham acupuncture group and the other 2 groups, but given the definition of clinically significant effects (ie, effect sizes [ESs] of approximately 0.5 SDs),^12,19,20^ this study was well positioned to detect clinically significant differences between any 2 groups. To allow for up to a 25% dropout rate, we randomized 399 participants.

To test our primary end point, analysis of covariance was applied to test the treatment difference 1 year after the end of radiation therapy, controlling for baseline XQ score and institution. Clinically relevant XQ scores were examined using χ^2^ tests. Exploratory analyses examined any group by institute interactions. Mixed-model analyses of repeated measures were used to examine changes in XQ scores over time using the MIXED procedure in SAS statistical software version 9.4 (SAS Institute) with the covariance type unstructured.^21^

## Results

### Enrollment

Of 680 patients screened (300 patients at Fudan and 380 patients at MD Anderson), 60 patients from Fudan and 221 patients from MD Anderson were excluded, mainly owing to not meeting eligibility criteria (eg, having previously received acupuncture, not having intact parotids), already being part of a different randomized clinical trial, being unwilling to consent, or being unable to accommodate the study schedule. The larger number excluded at MD Anderson was mainly due to challenges in accommodating schedules. This was less of an issue at Fudan because radiation therapy was delivered as an inpatient procedure. The remaining 399 eligible participants (58.7%) consented to participate and were randomized. There was a total of 132 patients in the TA group, including 79 patients at Fudan and 53 patients at MD Anderson; 134 patients in the SA group, including 81 patients at Fudan and 53 patients at MD Anderson; and 133 patients in the SCC group, including 80 patients at Fudan and 53 patients at MD Anderson (Figure 1).

### Patient Characteristics

There were no group differences in age, sex, disease stage, mean tumor dose, or type of treatment (Table 1). At baseline, there were no differences between institutions or groups in expectations of acupuncture’s effect on RIX symptoms. Likewise, there were no changes in expectations of acupuncture’s effect on xerostomia symptoms over time (eTable 4 in Supplement 2). There were group differences in mean (SD) XQ scores at baseline between institutions but not within institutions (Fudan: TA, 0.6 [2.3]; SA, 0.4 [1.3]; SCC, 0.5 [1.5]; MD Anderson: TA, 5.9 [11.2]; SA, 9.0 [15.3]; SCC, 8.4 [11.5]) (Table 2).

**Table 1.** Baseline Participant Characteristics

Variable	Fudan				MD Anderson			
	No. (%)^a^			*P* Value	No. (%)^a^			*P* Value
	TA (n = 71)	SA (n = 74)	SCC (n = 76)		TA (n = 47)	SA (n = 50)	SCC (n = 40)	
Age, y								
Mean (SD)	46.3 (11.5)	48.3 (11.1)	46.4 (10.4)	.45	57.5 (9.4)	57.7 (9.7)	58.7 (9.6)	.83
Median (range)	47 (21-71)	48 (31-79)	46 (22-76)		58 (29-76)	57 (26-75)	58.5 (38-78)	
Sex								
Men	55 (77.5)	55 (74.3)	58 (76.3)	.90	39 (83.0)	43 (86.0)	31 (77.5)	.57
Women	16 (22.5)	19 (25.7)	18 (23.7)		8 (17.0)	7 (14.0)	9 (22.5)	
Cancer stage								
1	3 (4.2)	2 (2.7)	1 (1.3)	.89	1 (2.1)	2 (4.0)	2 (5.0)	.99
2	9 (12.7)	8 (10.8)	11 (14.5)		3 (6.4)	3 (6.0)	3 (7.5)	
3	35 (49.3)	33 (44.6)	35 (46.1)		4 (8.5)	6 (12.0)	5 (12.5)	
4	24 (33.8)	31 (41.9)	29 (38.2)		39 (83.0)	39 (78.0)	30 (75.0)	
Dose sparing, >26 Gy								
Both sides	0	0	0	.35	12 (25.6)	13 (26.0)	7 (17.5)	.44
1 Side	1 (1.4)	0	0		27 (57.4)	32 (64.0)	30 (75.0)	
None	70 (98.6)	74 (100)	76 (100)		8 (17.0)	5 (10.0)	3 (7.5)	
Type of treatment								
Radiation only	12 (16.9)	9 (12.2)	12 (15.8)	.99	6 (12.8)	7 (14.0)	7 (17.5)	.98
Radiation with concurrent chemotherapy	5 (7.0)	6 (8.1)	6 (7.9)		26 (55.3)	25 (50.0)	19 (47.5)	
Radiation with induction chemotherapy	8 (11.3)	10 (13.5)	9 (11.8)		6 (12.8)	7 (14.0)	7 (17.5)	
Radiation with induction and concurrent chemotherapy	46 (64.8)	49 (66.2)	49 (64.5)		9 (19.2)	11 (22.0)	7 (17.5)	

Abbreviations: SA, sham acupuncture; SCC, standard care control; TA, true acupuncture.

To convert grays to rads, multiply by 100.

^a^ Includes all participants with baseline data plus at least 1 follow-up at any time.

**Table 2.** Unadjusted Mean XQ Scores With ES Estimates

Institution	XQ Score, Mean (SD)			TA vs SCC		SA vs SCC		TA vs SA	
	TA	SA	SCC	*P* Value^a^	ES^b^	*P* Value^a^	ES^b^	*P* Value^a^	ES^b^
Combined patients, No.^c^	118	124	116						
Baseline	2.7 (7.8)	3.8 (10.6)	3.2 (7.8)	.001	−0.44	.16	−0.19	.06	−0.26
1-y follow-up	25.5 (16.3)	31.1 (19.3)	33.8 (21.8)						
Fudan patients, No.^c^	71	74	76						
Baseline	0.55 (2.3)	0.35 (1.3)	0.5 (1.5)	.005	−0.48	.92	0.02	.004	−0.5
1-y follow-up	20.74 (13.9)	30 (19.9)	29.59 (20.3)						
MD Anderson patients, No.^c^	47	50	40						
Baseline	5.88 (11.4)	8.98 (15.3)	8.44 (11.5)	.07	−0.42	.01	−0.59	.45	0.16
1-y follow-up	32.91 (17.1)	32.90 (18.6)	42.94 (22.4)						

Abbreviations: ES, effect size; SA, sham acupuncture; SCC, standard care control; TA, true acupuncture; XQ, Xerostomia Questionnaire.

^a^ *P* values from mixed-model analysis of variance controlling for baseline XQ score and institution for combined analysis and baseline XQ score only for individual institution analysis.

^b^ Calculated from least square means and estimated SD from model.

^c^ Includes all participants with baseline data plus at least 1 follow-up at any time.

### Missing Data and Dropouts

Adherence to acupuncture treatments (3 times per week for 6-7 weeks of radiation therapy) was very high in both groups (95.9%). Participants were included in the analysis if they had a baseline XQ assessment and at least 1 follow-up XQ assessment (Figure 1). Of 399 patients randomized, 339 patients (212 patients at Fudan and 127 patients at MD Anderson) completed the 1-year follow-up assessment. The mean (SD) age of these patients was 51.3 (11.7) years (range, 21-79 years), and 258 (77.6%) were men. Although we estimated a 25% dropout rate, our actual dropout rate at 12 months was lower, at 15%. We compared the baseline demographic and medical characteristics of 339 patients with and 60 patients without XQ outcomes at the 12-month assessment. There were no group differences on any variable except that the group with missing data had a greater proportion of stage IV cancer at study entry.

### Xerostomia

In the analysis of covariance controlling for baseline XQ score and institution, the adjusted least square mean (SD) XQ score in the TA group (26.6 [17.7]) was significantly lower than in the SCC group (34.8 [18.7]) (*P* = .001; ES = −0.44) and marginally lower than in the SA group (31.3 [18.6]) but not significantly so (*P* = .06; ES = −0.26), and there was no difference between the SA and SCC groups (*P* = .16). In secondary analysis, the acupuncture groups (TA and SA) were combined and compared with SCC, revealing significantly lower adjusted least square mean (SD) XQ scores for acupuncture (28.3 [18.7] vs 34.0 [18.9]; *P* = .008). All analyses were also conducted controlling for baseline XQ, age, sex, stage, treatment type (induction and concurrent chemotherapy), and radiation therapy dose, and the results remained the same. There were no group differences when comparing dose sparing to both sides, 1 side, or neither side.

**Figure 2.**
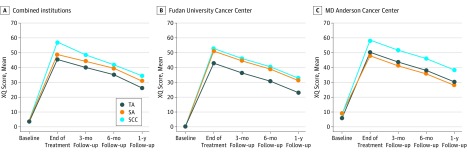
Least Square Means Derived From Mixed-Model Analyses of Xerostomia Questionnaire (XQ) Scores for Combined and Individual Institutions SA indicates sham acupuncture; SCC, standard care control; and TA, true acupuncture.

Exploratory mixed-model analysis of variance controlling for baseline XQ score revealed a significant center-by-group effect showing that for the patients in China, there were significant differences in adjusted least square mean (SD) XQ scores between the TA group (20.8 [21.0]) and the SCC group (29.6 [18.3]) (*P* = .005; ES = −0.48) and between the TA group and the SA group (29.9 [18.3]) (*P* = .004; ES = −0.5) but not between SA and SCC groups (*P* = .92). For patients in the United States, there was a marginal but not significant difference in adjusted least square mean (SD) XQ scores between the TA group (34.7 [17.7]) and SCC group (42.2 [17.6]) (*P* = .07; ES = −0.42) and a significant difference between the SA group (31.8 [17.6]) and the SCC group (*P* = .01; ES = −0.59) but not between the TA and SA groups (*P* = .44). The incidence of clinically significant xerostomia followed a similar pattern as did changes over time since the end of radiation therapy (Table 3; Figure 2).

**Table 3.** Incidence of Clinically Significant Xerostomia for Combined and Individual Institutions

Institution	Incidence, No. (%)^a^			*P* Value
	TA	SA	SCC	
Combined				
Baseline	3 (2.5)	4 (3.2)	1 (0.9)	.45
1-y follow-up	38 (34.6)	54 (47.8)	60 (55.1)	.009
Fudan				
Baseline	0	0	0	NA
1-y follow-up	15 (22.7)	32 (46.4)	36 (48.7)	.003
MD Anderson				
Baseline	3 (6.4)	4 (8.0)	1 (2.5)	.53
1-y follow-up	23 (52.3)	22 (50.0)	24 (68.6)	.21

Abbreviations: NA, not applicable; SA, sham acupuncture; SCC, standard care control; TA, true acupuncture.

^a^ Based on unadjusted mean scores higher than 30 for the Xerostomia Questionnaire.

In the mixed-model analyses of repeated measures (Figure 2), the time effect (parameter estimate [SE], −2.6 [0.38]; *P* < .001) and the quadratic time effect (parameter estimate [SE], −0.06 [0.02]; *P* = .007) were statistically significant. These findings suggest that XQ scores improved through time (time main effect) and that the rate of improvement decreased through time (quadratic time effect). There was no significant group by time or group by quadratic time interaction. The group main effect was significant (adjusted least square mean XQ [SE] score: TA, 36.5 [1.45]; SA, 40.5 [1.40]; SCC, 45.4 [1.46]; *P* < .001). Post hoc 2-by-2 comparisons revealed statistically significant differences between TA and SCC groups (estimated difference [SE] score, −9.0 [2.0]; *P* < .001), SA and SCC groups (estimated difference [SE] score, −4.9 [2.0]; *P* = .01), and TA and SA groups (estimated difference [SE] score, −4.0 [2.0], *P* = .046). In a separate mixed model, we found a significant interaction between group and institution (*F* = 4.0; *P* = .02). Post hoc comparisons revealed a significant difference between TA and SCC groups at both institutions, but TA was significantly different from SA only at Fudan (estimated difference [SE]: TA vs SCC, −9.9 [2.5]; *P* < .001; SA vs SCC, −1.7 [2.5]; *P* = .50; TA vs SA, −8.2 [2.5]; *P* = .001), and SA was significantly different from SCC only at MD Anderson (estimated difference [SE]: TA vs SCC, −8.1 [3.4]; *P* = .016; SA vs SCC, −10.5 [3.3]; *P* = .002; TA vs SA, 2.4 [3.2]; *P* = .45). The results from multiple imputation analyses using the MIANALYZE procedure in SAS were the same.

### Adverse Events

No adverse events reported at MD Anderson were related to acupuncture, and only 1 adverse event reported at Fudan was related to acupuncture (pain from needling at 1 site in the ear). Overall, there were no differences in adverse events reported between groups or institutions, with 185 adverse events reported at Fudan, including 33 in the TA group, 27 in the SA group, and 38 in the SCC group, and 185 adverse events reported at MD Anderson, including 24 in the TA group, 39 in the SA group, and 24 in the SCC group.

### Expectancy

Assessments of expectations, a measure of credibility, were measured at baseline for all patients and after 4 sessions and at the end of acupuncture treatment for the 2 treatment groups. The assessment revealed no group differences or differences between sites at any time point (eTable 4 in Supplement 2).

## Discussion

This is the first phase 3 randomized clinical trial to evaluate the use of acupuncture to reduce the incidence and severity of RIX in patients with head and neck cancer undergoing radiation therapy, to our knowledge. These results support previous findings from several smaller trials.^10,11,12,13,15,22,23^ As current methods for treating established RIX have shown little benefit, our findings indicate acupuncture may be a compelling adjunct to standard treatment for patients at risk of developing RIX, particularly since acupuncture has a low adverse effect profile and relatively low cost.

For the primary analysis, we were interested in the sample as a whole, controlling for baseline XQ score and site only. After receiving acupuncture 3 times per week during a 6- to 7-week course of radiation therapy, patients who underwent TA reported significantly less xerostomia 12 months after treatment than those in the SCC group. The ES was of medium magnitude, but the differences did not reach statistical significance (a 10-point difference, whereas TA vs SCC had a 9-point difference). Although sham-controlled clinical trials impart important information toward understanding putative mechanisms and a validated approach was used in this study, the choice of sham comparators in acupuncture trials is still highly debated. Thus, as other large, 3-arm acupuncture trials^24^ have demonstrated, the most relevant comparison is between TA and SCC. However, combining the 2 acupuncture groups also revealed significant differences vs SCC.

Site and site by group were included as part of secondary post hoc analyses. Patients in China who received TA experienced significantly less xerostomia than did those who received SA or SCC. In the United States, both TA and SA reduced xerostomia symptoms 1 year after radiation therapy compared with SCC. There was a significant group-by-institution effect, and although the reasons are unclear, there are several important issues to consider.

### Limitations

This study had some limitations. Importantly, participants in China were treated as inpatients, whereas US participants were treated as outpatients. Owing to logistics at Fudan, the acupuncture sessions were delivered in a busy, loud, semiprivate clinical space. At MD Anderson, treatments were delivered in a quiet, private room with dimmed lighting. Although it is unclear how this may have affected the study results, it could have influenced the sham response. We attempted to control for this by using an SCC control group and procedures that have been described elsewhere.^12,15^ Some positive effects associated with acupuncture treatment may be due to nonspecific factors, such as conditioning, expectations, self-empowerment effects, and cultural influence. A study by Kaptchuk et al^25^ found that the patient-practitioner relationship is the most robust component of a placebo effect, and this relationship is highly influenced by culture. In this study, aspects of the patient-clinician encounter were not highly controlled between centers. We did not monitor verbal interactions or document factors related to relaxation during the clinical encounter. This may explain some of the differences between centers and the lack of a placebo effect in Fudan. A recent report also documented an increase in the placebo response over time for clinical trials for neuropathic pain, with this effect only evident for trials conducted in the United States.^26^ Future studies should ensure that the context in which acupuncture is delivered is well controlled.

In addition, we did not systematically collect data on the use of salivary substitutes, and this may have differed by institution. Yet, as salivary substitutes are short acting (<4 hours) and patients were required to refrain from their use for 24 hours before data collection, it is unlikely that this influenced the findings. Although patients were instructed not to take any herbs of supplements while participating in the study, it is possible that patients did not adhere to this request, and this may have been more likely among the Chinese patients.

It is also possible that Chinese patients in this trial became unblinded, which could partially explain the significant group by institution effect. As there is greater cultural awareness of acupuncture in China, the patients may have noticed the use of more sham needle devices in the SA group, which could have changed their perception of the procedure. However, every effort was made to keep the patients from seeing the needles in all groups, and we used the same procedures as in our pilot studies^12,15^ where blinding was maintained.

Furthermore, the perception of the effects of real vs sham acupuncture may have a differential effect at the central nervous system level and may be moderated by culture. A 2008 study^27^ reported that cultural background moderates the activation of brain networks engaged during even simple tasks. In a separate study,^28^ we found that the placebo effect, as evidenced by activation of distinct brain regions, differed between Chinese and US acupuncture sessions. Specifically, brain activity in areas that have been identified as being influenced by culture showed increased activity in brains of East Asian individuals compared with brains of Western individuals during placebo acupuncture. In predetermined regions of interest, brain activity between placebo and real acupuncture conditions was minimally different in the brains of Western individuals, with larger differences noted in the brains of Chinese individuals.^28^

## Conclusions

In this randomized clinical trial, we found acupuncture to be superior to standard care in relieving symptoms of RIX. Although a cost-benefit analysis was not an aim of the current study, acupuncture is minimally invasive and has a very low incidence of adverse effects. No adverse effects related to the treatment, outside of mild discomfort from needle insertion, were reported by participants. On the basis of these findings, acupuncture may be considered an adjunct to standard care for patients who are interested in receiving acupuncture and at risk of developing RIX. Owing to the inconsistencies observed in this trial between treatment locations and the small to medium ESs, further studies are needed to confirm the clinical relevance and generalizability of our findings.

## Notes

Supplement 1. **Trial Protocol and Statistical Analysis Plan**

Supplement 2. **eTable 1.** Inclusion and Exclusion Eligibility Criteria **eTable 2.** Radiation Therapy Procedures **eTable 3.** Acupuncture Procedures **eReferences.** **eTable 4.** Acupuncture Expectation Score by Group

Supplement 3. **Data Sharing Statement**
