# Supplementary material for: Effect of True and Sham Acupuncture on Radiation-Induced Xerostomia Among Patients With Head and Neck Cancer: A Randomized Clinical Trial
Source: JAMA Netw Open. 2019 Dec 6;2(12):e1916910. doi: 10.1001/jamanetworkopen.2019.16910 (PMC6902763; doi:10.1001/jamanetworkopen.2019.16910)
Supplement: Supplement 1. — Trial Protocol and Statistical Analysis Plan [file jamanetwopen-2-e1916910-s001.pdf]

Supplementary Online Content

Table of Contents

		Page
**Supplement 1**	Original Study Protocol	**2**
	Original Statistical Analysis Plan	**11**
	Summary of Protocol Revisions	**22**
	Summary of Statistical Analysis Revisions	**24**
	Final Protocol	**25**
	Final Statistical Analysis Plan	**37**

Supplement 1: Study Protocol & Statistical Analysis Plan

Original Protocol

Acupuncture for Prevention of Radiation-induced Xerostomia (RIX) in Cancer Patients: Randomized, Sham-controlled Trial

1. SPECIFIC AIMS

Xerostomia (dry mouth) is a common problem among cancer patients who have received radiation treatment to the head and neck. Xerostomia often severely impairs quality of life (QOL), and available treatments have a low success rate. Patients suffering from radiation-induced xerostomia experience taste aberrations, dysphagia, odynophagia, difficulty sleeping and speaking, and loss of appetite. Such changes can lead to substantial nutritional deficits that can be difficult to reverse. Several pharmaceutical approaches to treatment, including saliva substitutes, chewing gum, lozenges, pilocarpine, and amifostine, have yielded limited results.

Studies conducted in the United States and Europe suggests that acupuncture is useful for treating radiation-induced xerostomia. No studies, however, have examined the use of acupuncture to reduce the severity of radiation-induced xerostomia or measured the duration of response to acupuncture treatment. Initial research conducted at The University of Texas M. D. Anderson Cancer Center (M. D. Anderson) and collaborative research at Fudan University Cancer Hospital (Fudan Cancer Hospital) in Shanghai, China, found that acupuncture can diminish symptoms in patients with xerostomia and could prevent the severity of xerostomia symptoms and improve quality of life in patients undergoing radiotherapy.

The proposed multi-center, randomized, placebo-controlled, single blind trial will examine the effectiveness of acupuncture at preventing radiation-induced xerostomia in patients undergoing radiotherapy for head and neck cancer at M. D. Anderson or nasopharyngeal carcinoma (NPC) at Fudan Cancer Hospital. Prior to receiving radiation treatment, patients will be randomized to one of three groups: Group 1 (G1) will receive true acupuncture, Group 2 (G2) will receive sham acupuncture, and Group 3 (G3) will receive standard care during the radiation treatment period. Subjective and objective measures of xerostomia will be collected prior to, during, and at the end of the radiation treatment period as well as 3, 6, and 12 months later. The aims of this study are to:

PRIMARY AIM

1. Determine if true acupuncture is more effective than sham acupuncture or standard care for preventing radiation-induced xerostomia among cancer patients at M. D. Anderson and Fudan Cancer Hospital. The primary outcome will be self-reported xerostomia symptoms using the Xerostomia Questionnaire (XQ).

SECONDARY AIMS

1. Examine group differences in salivary flow rates using unstimulated and stimulated whole saliva flow rates. This will provide a more objective measure of salivary function.
2. Determine if true acupuncture is more effective than sham acupuncture or standard care for reducing the incidence of radiation-induced xerostomia.
3. Examine whether true acupuncture results in better overall quality of life than sham acupuncture or standard of care.
4. Determine acupuncture's effects on saliva-based factors including pH, buffer capacity, and viscosity as well as levels of total protein and the neuropeptides calcitonin-gene-related peptide (CGRP) and vasoactive intestinal polypeptide (VIP).
5. Examine the role of expectancy for the benefits of acupuncture in predicting outcomes. The role of expectancy as a moderator of the effects of treatment will be thoroughly examined.

2. <u>RESEARCH STRATEGY</u>

A. <u>Significance</u>

The proposed research is important for several reasons. The salivary glands are uniquely sensitive to damage

from irradiation, and the development of radiation-induced xerostomia is clearly related to the exposure of salivary tissue. Mean parotid radiation doses >35Gy result in permanent xerostomia for most patients with no meaningful likelihood for recovery (1). Reduction in salivary flow begins during the first few days of treatment, with an 80% decrease noted at 6 weeks. In addition to a decrease in volume, other quantitative changes noted are a decrease in salivary pH, an increase in viscosity, and a reduction of salivary constituents (i.e., immunoglobulins, buffering capacity, small organic molecules) (2, 3).

Due to the high prevalence of smoking in China (4), head and neck cancer rates are high. In China, the incidence of nasopharyngeal cancer (NPC) is higher than in Western countries (5, 6). Available statistics indicate that the incidence of head and neck cancers in Shanghai is 14.1/100K and 6.5/100K for men and women, respectively (7). Among these, approximately 1000 are treated annually with radiation therapy at Fudan Cancer Hospital, and almost all patients receiving radiation to the head and neck area develop xerostomia. Furthermore, in the United States, nearly 40,000 patients are diagnosed annually with head and neck cancer, and approximately 80% will receive radiotherapy (8). In FY08, more than 1000 patients diagnosed with head and neck cancer were seen at M. D. Anderson and 466 underwent radiation treatment.

During the past decade, several new strategies to prevent radiation-induced xerostomia have been investigated including: 1) improved targeting of the radiation beam through intensity-modulated radiation therapy (IMRT); 2) development of radioprotective agents such as amifostine, and; 3) new surgical techniques that involve relocation of the submandibular gland to the submental space before radiation treatment. These approaches are limited by several factors, including technological constraints, primary site of disease, and proximity of the tumor and involved lymph nodes to the parotid glands. Treatment for xerostomia is, at best, palliative for symptom relief. Several approaches, including saliva substitutes, chewing gum, lozenges, pilocarpine, and amifostine, have been attempted with limited results and low acceptance due to unpleasant side effects (9-17). Also, pilocarpine is not commonly used in China, where there is currently no widely accepted treatment for radiation-induced xerostomia. Although IMRT has been shown to reduce the incidence of late grade 2 xerostomia (18, 19), all patients do not receive IMRT, and it is not commonly available in many areas of China. Also, IMRT only partly spares the parotid glands and minor salivary glands within the oral cavity; thus, the development of xerostomia remains high (20-24). In a pilot trial at M. D. Anderson investigating the use of acupuncture to treat radiation-induced xerostomia (25), most participants had received IMRT, yet these patients still benefited from the acupuncture treatment (see Preliminary Studies C.8).

Acupuncture therapy has been practiced in China and other regions for thousands of years. According to the World Health Organization, it is used in at least 78 countries worldwide (26). The therapeutic effects of acupuncture treatment range widely, and Eastern cultures commonly use it for a variety of conditions from alleviation of general fatigue to the treatment of specific diseases (27). According to traditional Chinese medicine (TCM), acupuncture points are specific sites along meridians that are related to internal organs. Modern anatomical and physiological studies have found that these acupoints are areas on the skin surface with lower electrical resistance and increased conductance compared to that of their immediate environment (28). Some studies have shown that acupuncture points correspond to peripheral cranial and spinal nerve endings (29). To stimulate these points, the most common technique remains penetration of the skin by thin, solid, metallic needles, which are manipulated manually or by electrical stimulation.

From a TCM perspective, loss of body fluids is considered a Yin deficiency syndrome (30). Although Yin deficiencies may be related to various organ systems, xerostomia is primarily considered a deficiency of the Stomach and/or Kidney Yin. Individual patients may concomitantly exhibit signs and symptoms of Lung Yin, Heart Yin, and/or Qi-Blood deficiencies as well as any number of other underlying imbalances. Most xerostomia studies conducted to date that base acupuncture point selection on classical TCM theory have been designed to address Stomach and/or Kidney Yin deficiency and “clear heat.” Heat in TCM dries body fluids and can occur in both excess and deficiency states. In xerostomia patients, excess heat is caused from the radiation and deficiency heat is caused by the underlying Yin imbalance.

A number of positive clinical trials (25, 31-41) have been conducted examining the use of acupuncture to treat radiation-induced xerostomia (See Appendix C for a Table summarizing the findings). However, two systematic reviews (42, 43) indicated that most of these trials have been small and methodological rigor is often lacking, indicating the need for larger randomized trials.

Neuronal substrates involved in changes in saliva production after stimulation of acupuncture point LI-2 have been explored by Deng and colleagues (44) using functional magnetic resonance imaging. In this randomized, sham-controlled cross-over trial, 20 healthy volunteers received both true and sham acupuncture in random order. Unilateral manual acupuncture was associated with bilateral activation of areas of the brain where gustatory, olfactory, visual stimuli and signals from expectation/suggestion are integrated. Sham acupuncture did not produce this effect. Furthermore, true acupuncture caused significantly more saliva production in participants than sham acupuncture ($p=0.02$), and there was a positive correlation ($r=0.63$) between the amount of saliva produced and changes in the regions of interest volume. This brain imaging trial, along with the other small positive studies, suggests further research is needed in this area. Finally, there needs to be a better understanding of the putative biological mechanisms involved in the treatment of xerostomia using acupuncture.

This trial seeks to improve scientific knowledge regarding the benefits of acupuncture for xerostomia by conducting a multi-center, placebo-controlled, phase III clinical trial of acupuncture to prevent xerostomia. In addition to self-report measures of xerostomia and quality of life (QOL), we will quantify changes in saliva flow and examine potential mechanisms through quantitative assessment of changes in saliva following acupuncture treatment. Although other studies (2, 3) have evaluated salivary viscosity, pH, and buffering capacity related to xerostomia, changes in these measures in response to acupuncture have not been investigated. We will also expand data from previous acupuncture studies (38, 39) reporting changes in objective indices by measuring changes in calcitonin-gene-related peptide (CGRP) and vasoactive intestinal polypeptide (VIP).

B. <u>Innovation</u>

Since the mid-1990s, studies conducted by different investigators from both the United States (25, 31, 35, 44-47) and Europe (34, 36-41, 48, 49) have shown that acupuncture can have a positive effect on symptoms related to radiation-induced xerostomia. Patients have reported relief in as few as 5 treatments with benefits lasting up to 3 years. Only our two studies (see sections C.8 and C.9), however, have examined whether acupuncture can prevent this often-debilitating side-effect of radiation treatment from occurring or if it can diminish the symptoms once it develops. There have also been no large, definitive, phase III placebo- controlled trials to determine whether acupuncture is an effective treatment for managing radiation-induced xerostomia.

Most previous trials of acupuncture to treat xerostomia have been single-institution studies. Furthermore, few studies have examined NPC, as it is not prevalent in Western countries, which is where all the previous research on acupuncture for xerostomia has been conducted. The proposed trial is unique in that we will extend the findings from our phase II trial and pilot placebo-controlled trial conducted at Fudan Cancer Hospital by now conducting a multi-center, placebo-controlled, phase III clinical trial of acupuncture to prevent xerostomia in patients with NPC and other head and neck cancers (mainly oropharyngeal cancer). The inclusion of a placebo control group will allow us to more definitively determine the benefits of acupuncture for preventing this difficult-to-manage side effect of radiotherapy to the head and neck. Examining the effects within two different cancer populations, at two different centers, in two different countries, will increase the generalizability of our findings. Importantly, in addition to using validated self-report measures for xerostomia accepted by the Food and Drug Administration as the “gold standard” for the approval of treatments for xerostomia, we will assess more general aspects of QOL, assess objective measures of salivary flow, and quantify changes in potential mechanisms through assessment of salivary constituents.

Another innovative aspect of this study involves evaluation of participant expectations on outcomes. This will allow further exploration of how psychological phenomena may be related to outcomes in acupuncture research. Although well-conducted, placebo-controlled clinical trials have offered insights into the efficacy of acupuncture, the substantial effect seen in many placebo acupuncture groups presents a significant challenge in interpreting treatment results. Many studies, including several meta-analyses (50-56), have found that both real and placebo acupuncture produce statistically and clinically significant changes when compared to no treatment or standard/enhanced medical care. These findings suggest that the effect found in placebo acupuncture groups cannot be entirely attributed to either regression to the mean or natural disease processes; thus, the “non-specific” effect of acupuncture must be responsible in part for patients’ clinical response. Nevertheless, until we can parse the components that make up the “non-specific” effect, this effect, powerful as it may be, is likely to be discarded from a scientific standpoint. Years of psychological research has found that expectancy is one of the central pieces in this so-called “non-specific” effect. In particular,

response expectancy, a form of outcome expectancy defined as “expectations held by the individual about one’s own emotional and physiological response” related to a situation or therapy, may produce important clinical changes (57). In a systematic review, Crow et al. (58) showed that when clinicians stated positive outcome expectations as opposed to uncertain expectations, most studies found improvements in patient self-reports of anxiety, pain, and distress.

A few studies have explored the association between expectancy and clinical outcomes in the context of acupuncture (59, 60). While these studies provided preliminary evidence that greater expectancy may produce better clinical response, none used a validated instrument to measure expectancy. In the proposed study, we will use an expectancy questionnaire that has been validated in both English and Mandarin (61). Investigating expectancy as part of the complex mind-body interactions of acupuncture care will likely yield important knowledge that is both scientifically sound and clinically meaningful to patients who suffer from xerostomia and other distressing symptoms.

C. Approach

C.1 Overview and Design

The primary purpose of this trial is to investigate whether true acupuncture is more effective than sham acupuncture or standard care for preventing radiation-induced xerostomia. Prior to radiation therapy, patients will be randomized into three groups: G1 (n=100) will receive true acupuncture, G2 (n=100) will receive sham acupuncture, and G3 (n=100) will receive standard care. Patients in the acupuncture treatment groups will receive treatments 3 days a week for 6 - 7 weeks. The duration of acupuncture therapy in the proposed study is the same as in our pilot trials. It has been shown to have a good effect in our phase II trial and small placebo controlled trial conducted at Fudan Cancer Hospital. Furthermore, inconvenience is minimized for participants because acupuncture is provided on the same day as radiation therapy.

Potential study participants will be identified by faculty in the departments of Radiation Oncology at Fudan Cancer Hospital and M. D. Anderson and referred to the research nurse for assessment of eligibility and informed consent counseling. Potentially eligible patients will be contacted in person when they are in the clinic so that the research nurse can describe the study in detail, answer the patients’ questions, and assess their interest in the study. Half the patients in each group (n=50) will be recruited from each center (150 patients/center) for a total sample size after attrition of 300 patients (375 patients before attrition) (see Appendix D. Study Schema, Study Schedule, Study Timeline).

C.2 Eligibility and Participants

Inclusion criteria: Adult ≥ 18 years of age and able to give informed consent; Diagnosed with head and neck cancer, which will be primarily oropharyngeal, and scheduled to undergo IMRT with or without concurrent chemotherapy at M. D. Anderson; Treatment plan that includes external beam radiation at a mean dose of at least 25 Gy or more to one of the parotid glands (the other gland can receive less than 25 Gy); Anatomically intact parotid and submandibular glands; Karnofsky performance status > 60 (see Appendix E).

Exclusion criteria: History of xerostomia prior to head and neck radiation therapy or history of Sjögren's disease or another underlying systemic illness known to cause xerostomia; Prior head and neck radiation treatment; Suspected or confirmed physical closure of salivary gland ducts on either side; Known bleeding disorders or taking any dose of warfarin or heparin; Upper or lower extremity deformities that could interfere with accurate acupoint location or alter the energy pathway as defined by traditional acupuncture theory; Local skin infections at or near the acupuncture sites or active systemic infection; History of cerebrovascular accident or spinal cord injury since the mechanism of acupuncture may be associated with central nervous system activity; Mental incapacitation or significant emotional or psychiatric disorder that, in the opinion of the investigator, precludes study entry as these patients may not be able to cooperate with this slightly invasive procedure or with the data collection process; Current acknowledged use of any illicit drugs or evidence of alcohol abuse as defined by The American Psychiatric Association criteria (62); Participants who are taking, or who have taken any investigational new drug within the past 30 days, or who are planning to take such a drug during the course of the study; Current acknowledged use of other alternative medicines, such as herbal preparations, that could affect salivary function. If a patient is using

any salivary substitute, they will be asked to refrain from using for at least 24 hours prior to saliva and questionnaire data collection. Each patient will be asked for a list of herbal supplements they are currently taking, and this will be reviewed on an individual basis. Patients will be excluded if they are taking any herbs known or suspected to affect salivary function. Participants taking amifostine, cholinergic agonist medications (pilocarpine, cevimeline), beta adrenergic antagonists, anticholinergic agents, saliva substitutes or other medications known to affect salivary function will also be excluded. All patients will be prohibited from taking these medications while on study (see Appendix F). If a patient reports use of one of the prohibited medications, they will be considered non-compliant and dropped from the study. Finally, participants will be excluded who are currently receiving acupuncture for any condition or if they have ever used acupuncture before.

Participants: Over 400 patients undergo IMRT radiation for head and neck cancer each year at M. D. Anderson or one of its satellite clinics. Within the next 2 years at least two more satellite clinics will open, increasing the possible patient base. At Fudan Cancer Hospital, approximately 450 patients undergo IMRT for NPC each year. Our pilot study experience at both institutions suggests that approximately 70% of these patients will be eligible for the proposed study. Therefore, at least 500 patients will be eligible per year for a total of over 1500 patients during the 36 months designated for recruitment.

All eligible patients will be asked to participate. We expect a relatively modest acceptance rate of approximately 60%. Patients generally decline either because they do not want to be randomized or because of time constraints. This conservatively estimated patient accrual rate would permit us to recruit at least 70 patients per year at each institution. In our previous studies, the attrition rate was less than 20%; however with longer follow-up, we expect the rate will increase somewhat. Thus, with even slightly higher attrition, we will still be able to accrue at least 50 evaluable patients per year per institution, for a total of 300 evaluable patients over the approximate 3 years of recruitment (n=100 per group; n=125 per group before attrition).

C.3 Procedures

After informed consent and prior to randomization and radiation treatment, the research nurses will collect baseline measures. Patients will be informed the study is examining the effects of two different forms of acupuncture delivered during radiotherapy. This is important because in most previous sham-controlled acupuncture trials patients could accurately guess group assignment. In this trial, patients in both acupuncture groups are in fact getting real needles inserted at real acupuncture points on the body and, therefore, this is technically classified as real acupuncture. Therefore, this is not deception and, in fact, we do not actually know whether G1 will have any better outcomes than G2. In reality, we are testing two forms of acupuncture. When a patient in G2 finishes the trial, we will debrief them to let them know that they were in an acupuncture group that we were uncertain if it would work or not. We will offer them three free sessions of acupuncture after the 12 month follow-up assessment.

A modified medical examination of participants will be obtained that includes a medical history and physical; review of systems, concomitant medications, and head and neck malignancy; surgical and radiation history; and planned radiation treatment. Self-report measures and sialometry measurements, including unstimulated whole salivary flow rate (USFR) and stimulated whole salivary flow rate (SSFR), will be completed. In order to identify potential mechanisms associated with acupuncture elicited stimulation of saliva secretion, measurement of pH and buffering capacity will be performed on fresh saliva from SSFR. Samples will then be frozen for later analysis of viscosity, total protein and neuropeptides, including CGRP, and VIP. After all analyses are complete, the saliva samples will be destroyed.

At each subsequent visit, vital signs (blood pressure, pulse) will be taken, and medications will be reviewed prior to the acupuncture treatment. All measures will be collected again during the course of radiation treatment (mid-treatment; except the assessment of salivary constituents), and at the end of the radiation treatment period (week 7). In the middle and end of radiotherapy, patients in G1 and G2 will indicate whether they think they were assigned to active or inactive acupuncture. Patients will also be asked to complete a form about their oral hygiene. All patients will be provided with standard oral care recommendations. These include keeping their mouth clean by brushing their teeth at least two times a day and preferably after each meal.

They will be also encouraged to not use candies, lozenges, or drink sugary beverages. They will also be told to consume up to 1.5 liters a day of water to help diminish the effects of radiation-induced xerostomia and keep hydrated.

Follow-up assessments will be obtained 3, 6, and 12 months after the end of radiation treatment for a total of 6 assessments (baseline; middle of radiotherapy; end of radiotherapy; and 3, 6, and 12 months after the end of radiation treatment). Participants will be given a small gift certificate (100 RMB [approximately $15] value at Fudan Cancer Hospital; $35 value at M. D. Anderson) for completing each assessment. After all data are collected at the 12-month follow-up visit, patients in the G2 and G3 study arms will be offered true acupuncture treatment for free for one week (3 treatments). For the complete study schema, schedule, and timeline, please see Appendix D. All data collection will be conducted by research staff who are blinded to group assignments. A procedures manual will be developed that will include all aspects of conducting the trial, including the acupuncture treatments.

Patients will be randomized by a form of adaptive randomization, called minimization, because simple randomization could result in covariate imbalances (63). Statistical adjustment of covariates can take imbalances into consideration, but results are generally more credible when they are obtained from groups with comparable baseline distributions. In minimization, group assignment is done sequentially. In this study, the patient characteristics used for group assignment will be stage of disease (I – IV), age (dichotomized above and below the running mean or equal to median of age), sex (M, F), the mean planned parotid doses (left and right side will be calculated separately and balanced between groups; <3000, 3001-5000, 5001-10000, 10,001-15,000, >15,000), induction therapy (yes/no), concurrent chemotherapy (yes/no), and type of chemotherapy received (1= high dose cisplatin (1 x every 3 weeks); 2= low dose cisplatin (weekly); 3=taxol/carboplatin; 4= cituximab; 5=carboplatin; 6= gemcitabine (for Shanghai only)). Randomization will be conducted at each site separately. This will ensure equal distribution of all factors across all groups and balanced at each site. Information on group assignment will be only communicated to the acupuncturists by the data managers to insure that the research staff remains blinded.

C.4 <u>Acupuncture Treatment Procedures</u>

Treatments will be given in the acupuncture clinics at M. D. Anderson and Fudan Cancer Hospital. Patients will be placed in a supine position and, thus, blocked from seeing any of the needles. The needles will remain in place for 20 minutes with each treatment. Standardized techniques for point location will be utilized, which are based on anatomical landmarks as well as proportional measurements using the patient's own body. For example, finger breadth is based on each patient's middle finger, and the proportional unit of measure, the "cun," is defined as the distance between the two medial ends of the creases of the interphalangeal joints when the middle finger is flexed (30, 64). The acupuncture procedures will be performed using standard aseptic technique (64). The needles come in individual sterile packages, and the same level of sterility as that used for an invasive medical procedure will be applied.

All treatment providers will be licensed, experienced acupuncturists. The acupuncturist and research nurses at Fudan Cancer Hospital have been trained at M. D. Anderson. Any new acupuncturists will have one-on-one training with the lead research acupuncturist from M. D. Anderson (Dr. Kay Garcia). All acupuncturists will also participate in a video-based training program. At the end of the training period, a return demonstration of 10 treatments (5 active and 5 sham) on individuals who are not potential study subjects will be required. Finally, in order to ensure continued quality control, participating acupuncturists will be observed and evaluated three times per year. Faculty and staff from M. D. Anderson will also visit Fudan Cancer Hospital twice a year to check on the running of the trial. We are confident in the quality control procedures in place as we have been conducting collaborative research now for over 6 years, including the specifics of this protocol in two different trials at Fudan Cancer Hospital.

a. <u>True acupuncture</u>

Although many point combinations could be used, the investigators have attempted to identify a set of acupuncture points that integrates TCM and biomedicine, with a focus on using a minimal number of sites. The active acupuncture points for this study were selected on the basis of successful experience from our pilot studies as well as on previously published trials (35-41, 45, 46, 65-67). Points were also selected on the basis

of their indications according to the classical theory of TCM (30, 64) and current understanding of the various anatomical locations and neurovascular tissues associated with each point (68).

The body acupuncture points selected for this protocol are Ren 24, Lung 7 (LU 7), and Kidney 6 (K6). One placebo needle will also be placed at Gallbladder 32 (Gb32) on the right side. This is intended to provide participants in the active treatment group with a stimulus that will not elicit De Qi sensation. Ear points selected are Shenmen, Point Zero, Salivary Gland 2' (SG 2-prime), and Larynx. Except for Ren 24, which is located in the midline and the placebo needle at Gb32, all points will be treated bilaterally. The only facial point used in this study will be Ren 24. After careful consideration and discussion, we have chosen not to use points on the face that were selected in some prior studies (i.e., Stomach 4-7). Even though problems are rare, we prefer to err on the side of precaution and avoid needling tissues that may still be friable or easily injured after radiation. Furthermore, Johnstone et al. (35, 45, 46) obtained good results without using facial points. Ren 24 is located in the midline and should not pose a problem. Patients will be excluded from the study, however, if there is any indication of skin irritation or infection at this location.

Needle insertion endpoints will be the standardized recommended depth of insertion (30, 64) or achievement of De Qi sensation per the acupuncturists' determination. De Qi is a feeling of tingling, aching, numbness, or warmth at the acupuncture site. Acupuncturists also describe this feeling as if the muscle is grabbing the needle. Propagation of this sensation along acupuncture channels is thought to carry the therapeutic signal to target areas (30, 68). Once the standard depth of insertion or De Qi is achieved at the body points, the needles will not be manipulated further unless one becomes displaced. No electrical stimulation will be applied to the needles in this study. Acupuncture needles used for the body points will be 0.25 x 40-mm in length and for the ears 0.16 x 15-mm in length (see Appendix G for further details).

b. <u>Sham acupuncture</u>

Well-designed clinical acupuncture trials require a sham procedure that is indistinguishable from the real treatment, yet inactive. Although no "gold standard" has been established for placebo controls in acupuncture trials, ideally, non-penetrating needles placed at inactive points should be used. Park and colleagues validated a non-penetrating, telescoping needle with a separate device that attaches it to the skin (69, 70). This device will be used on body points in the proposed study but is too heavy and bulky for use on auricular points. For sham auricular points, Usichenko and colleagues have used points on the helix of the ear with success in 3 different studies (71-73). We propose to use a similar procedure for this study.

The sham procedure for participants in G2 is: Sham Location 1 - placebo needle at inactive point located 0.5 cun below and 0.5 cun lateral to CV 24 on the chin; Sham Location 2 - placebo needle at inactive point located 0.5 cun radial and 0.5 cun proximal to SJ 6 between SJ and LI Channels (bilateral upper extremities); Sham Location 3 - placebo needle at inactive point located 1.0 cun below and 0.5 cun lateral to St 36, between St and Gb Channels (bilateral lower extremities). In order to elicit De Qi in the control group, one 0.25 x 40mm acupuncture needle will be used at Gb 32 above the right knee. This point is not indicated for dry mouth. Finally, four 0.16 x 15mm acupuncture needles on the helix of each ear (8 ear points) will be included. Location of inactive points will be confirmed with an electrodermal point finder.

The sham treatment will be given according to the same schedule as the true acupuncture treatment. Participants in both groups will be placed in a comfortable position. Each point will be identified and marked on the skin. A total of 14 points will be used in both groups. As the active acupuncture group is receiving treatment using a placebo needle and the placebo acupuncture group is receiving active treatment at a real acupuncture point (both at Gb 32) and with acupuncture needles inserted at inactive points on the ear, the blinding of the two groups should be excellent. In the two-arm placebo-controlled pilot study conducted at Fudan Cancer Hospital using the same two acupuncture groups (G1 and G2), perfect blinding was indicated (see C.9 below).

c. <u>Usual care</u>

Participants in G3 will receive standard care. This will include being provided with standard oral care recommendations. Participants in all groups will receive the same recommendations.

C.5 Evaluation

All questionnaires will be administered at the patient's clinic visits. If, however, there are time constraints, patients will be given a self-addressed stamped envelope in which to return the questionnaires. Baseline assessments will be completed within 7 days prior to starting radiotherapy, and patients will then complete the same assessments again at middle and end of radiotherapy and 3, 6, and 12 months after the end of radiotherapy. At middle and end of radiotherapy, data will be collected before acupuncture treatment. For patients at Fudan Cancer Hospital, the questionnaires were forward and backward translated into Mandarin Chinese (the MDASI and FACT-G were also validated in Mandarin Chinese; see Appendix H). All of the questionnaires and saliva collection techniques have been used successfully at both institutions.

To determine if true acupuncture is more effective than sham acupuncture or standard care for reducing the incidence of radiation-induced xerostomia we will evaluate xerostomia graded according to Common Terminology Criteria for Adverse Events (CTCAE) (78) (see Appendix I).

a. Xerostomia and quality of life questionnaires

i. Xerostomia Questionnaire (XQ): The XQ is an 8-item questionnaire that has been validated in several cohorts (22, 74). Subjects rate each symptom on an 11-point ordinal Likert scale from 0 to 10, with higher scores indicating greater dryness or discomfort due to dryness. The item cores are added and the sum is transformed linearly to produce a final summary score ranging between 0 and 100, with higher scores representing more xerostomia. The clinical response of the XQ has been suggested by Eisbruch (22) and Pacholke research (74) with XQ scores ≤ 30 regarded as mild to no symptoms of xerostomia.

ii. The Functional Assessment of Cancer Therapy (FACT-G): Health-related quality of life will be assessed with the FACT-G (77). This instrument is able to discriminate between individuals with metastatic and non-metastatic disease, as well as between patients at different stages of illness. The scale has been found to have good concurrent validity, high internal consistency (0.89), and good test-re-test reliability (0.82 to0.88).

iii. M. D. Anderson Symptom Inventory for Head and Neck Cancer (MDASI-HN): Cancer-related symptoms will be assessed using the validated MDASI-HN (75, 76). The MDASI measures, on a numeric rating scale of 0-10, both the severity of symptoms and the interference symptoms cause in patients' daily activities. The 13 core MDASI symptom items are based on extensive evaluation of symptoms common to cancer and cancer treatment. The MDASI-HN includes 9 head and neck–specific items. The instrument was validated in a cohort of more than 200 patients and found to be highly reliable (76).

iv. Fatigue will be assessed using the Brief Fatigue Inventory (BFI) [95]. The BFI is a 9-item questionnaire designed to be used in the clinical setting to rapidly assess fatigue severity. The items are ranked from 0 to 10, and patients rate their fatigue at its "worst" and "usual" and as it is "now," with 0 = "no fatigue" and 10 = "fatigue as bad as you can imagine." Patients also rate how much their fatigue has interfered with their life.This single-dimension instrument was tested in a sample of 305 patients with cancer, and provided an internally stable measure of fatigue severity (0.80-0.92) [95].

v. The Brief Pain Inventory (Short Form) is a validated, widely used, self-administered questionnaire to assess severity of pain and impact of pain on daily functioning among patients (101-103). Item 1 assesses whether or not patients are currently experiencing pain other than minor headache, sprain, or toothache. Item 2 allows patients to indicate on a whole body diagram the exact location of pain. Items 3-6 measure severity of pain on a 0-10 numeric scale. Item 7 assesses current treatment and item 8 assesses relief from pain treatments or medications within the last 24 hours. Finally, item 9 includes 7 subratings regarding the impact pain within the previous 24 hours has had on patients' QOL.

vi. Depression will be assessed using the Centers for Epidemiological Studies-Depression measures (CES-D) [98]. The CES-D is a well-validated 20-item self-report measure of depression that focuses on affective components of depression. Respondents rate the frequency of the behavior or feeling using a 4-point Likert-type scale ranging from "almost never" to "almost always." The internal consistency of the instrument is high in the general population and in patient populations. It also has demonstrated adequate convergent validity with

other measures of depression [98].

vii. To thoroughly evaluate social processes, we will assess the availability of social support perceived by participants, the frequency and quality of their social interactions, and the level of expression of emotions with others. Perceived availability of social support will be measured using the Medical Outcomes Study Social Support Survey (MOS-SSS) [99], which was developed with the SF-36 quality of life index and other MOS measures in one of the largest and most comprehensive studies of health status in the chronically ill. The scale focuses on the perception of the availability of functional support. Patients rate the perceived availability of: emotional/informational support, tangible support, positive interactions, and affectional support. Internal consistency was high, ranging from 0.91 to 0.96, and test-retest reliability ranged from 0.72 to 0.76. It has good predictive validity reflecting significant relations between social support and overall health status [100]. We have also added a few questions that ask how satisfied they are with the contacts.

viii. Two items assessing behavioral and characterological self-blame for patients' cancer cause will be administered based on previous work in breast cancer patients (96). Patients will also complete two treatment regret items previously used by Hu et al. in a sample of prostate cancer patients (97). These questions will take about 1 minute to complete.

ix. Self-report measure of oral hygiene will be assessed at baseline, weekly during radiotherapy, and then 3, 6, and 12 months after the end of radiotherapy. Patients will be asked to indicate in the past week the frequency with which they brushed their teeth, flossed their teeth, and estimate how much water they consumed on daily basis.

b. Saliva flow examination

Unstimulated Whole Saliva (USFR): Patients are instructed to refrain from eating, drinking, and dental hygiene for a minimum of 60 minutes before saliva collection. The patient is seated upright in a quiet area where he/she will remain undisturbed and to minimize orofacial movements. The patient will relax in the designated area for 15 minutes before saliva collection. Patients are instructed not to attempt to increase or control salivation actively (such as sucking or swallowing) but to simply relax. A collection vial (including seal and cover) will be weighed on a calibrated balance with accuracy to 0.001 gm. The patient is instructed to first clear his/her mouth by swallowing. Then, with the head held slightly forward, the patient is instructed not to talk or swallow during the 5-minute collection but to allow saliva to collect in the floor of the mouth. The 5-minute collection time is initiated on a digital timer and in view of the patient. The patient should then expectorate the accumulated saliva into the pre-weighed vial after 60 seconds. The patient should repeat this procedure 4 more times for a total collection time of 5 minutes. Patients are reminded not to swallow during the entire collection period. At the end of the 5 minutes, the collection vial is promptly sealed, weighed, placed on ice and transported to the laboratory.

Stimulated Whole Saliva (SSFR): Patients are to rest for 5 minutes prior to stimulated saliva collection. The exogenous stimulant will be a neutral chewing gum from Wrigley (unflavored gum base) used previously for SSFR. The patient will chew the gum for 3 minutes, then expectorate the gum and saliva into a disposable cup. Next, the patient should swallow to clear the mouth. The patient's saliva will then be collected in the vial for 5 minutes using a method identical to USFR collection. After a 5 minute rest period, repeat (i.e. chew gum for 3 minutes, expectorate gum and saliva into a disposable cup, swallow, then collect saliva for 5 minutes as above.)

c. Saliva constituent analyses

We will determine acupuncture's effects on saliva-based factors including pH, buffering capacity, and viscosity as well as levels of total protein, CGRP and VIP from SSFR collected at baseline, end of radiotherapy (week 7), and 3, 6, and 12 months later. The value of pH and buffering capacity will be measured using a portable pH meter with micro electroprobe. Buffering capacity will be measured using the method previously described by Siqueria et al. (79). Buffering capacity and pH will be conducted on fresh saliva. Viscosity, total protein, CGRP, and VIP will be measured at M. D. Anderson from frozen samples assayed in batch to decrease variance. Viscosity of the saliva (0.5 ml) will be determined using LVT Wells-Brookfield cone-and-plate digital viscometer (Brookfield Engineering Laboratory) (80). The total amount of protein in each saliva sample will be determined using the Bradford assay (Biorad protein assay kit). Levels of CGRP and VIP will be quantified using

neuropeptide-specific RIA kits (Peninsula-Bachem) (81). The concentration of peptides will be normalized by the amount of protein in each sample. All assays will be conducted in the laboratory of Dr. Peiying Yang. Once the saliva samples have been assayed they will be destroyed.

d. Expectations

Acupuncture Expectancy Scale (AES): To determine the relationship between outcome expectancy related to acupuncture and clinical response, we will use the Acupuncture Expectancy Scale. This 4-item instrument was developed by Mao et al. and found to be reliable (Cronbach's α of 0.82) and valid by positive correlation with patient self-reported efficacy and satisfaction (61). The scale was further validated among cancer patients who were mostly acupuncture naïve (57). Expectancy of a benefit from acupuncture appeared to be increased by educating patients on the scientific theory and clinical evidence of acupuncture. Higher expectancy was also found in patients who had previously participated in an acupuncture trial compared with those who had not participated in an acupuncture trial (57). The scale has been translated and validated in Mandarin in China. In the proposed study, we will evaluate expectancy as a predictor of response to acupuncture. This questionnaire will be completed at baseline by all participants. Patients in the acupuncture treatment groups will complete the form again during the middle and at the end of radiotherapy.

e. Background/Exposure

Background/Exposure Questionnaire variables: The purpose of this questionnaire is to collect information on environmental and occupational exposures, phenotypic characteristics, health history, family history of cancer, and behavior patterns that could be associated with cancer of the head and neck. The data collection instrument will be comprised of the interview-based questionnaires that are currently used at MDACC. The questionnaire includes questions on the following items:

-- Demographic data, including name, address, date and place of birth, marital status, ethnicity, total years of education, and income.
-- Tobacco and alcohol exposure, including past and present smoking and drinking status, number of years smoked/drank, and number of cigarettes smoked or drinks consumed/day.
-- Behavior patterns that could affect an individual's exposure.
-- Medical history information on previous health such as history of cancer, immune conditions, and hormone and x-ray treatment.

C.6 Statistical Considerations

As our first step, before analyses, we will conduct extensive descriptive analyses on the data collected at baseline and at each follow-up. Descriptive statistics (i.e., frequencies, ranges, means, proportions, standard deviations, measures of skewness and kurtosis), including 95% confidence intervals (CIs), will be computed for the measures. We will closely examine distribution characteristics of the variables using box plots, histograms, scatter plots, and the Kolmogorov-Smirnov test of normality where appropriate. Distribution assumptions will be evaluated, and if indicated, normalizing transformations or robust procedures will be used. We will evaluate bivariate associations between the outcome measures and selected demographic and disease-related variables, including age, ethnicity, time since diagnosis, and disease stage, using Pearson product-moment correlation coefficients, chi-square, or eta-squared estimates from analyses of variance where appropriate.

a. Data analysis

Our primary objective is to determine if true acupuncture is more effective than sham acupuncture or standard care for preventing radiation-induced xerostomia among cancer patients at M. D. Anderson and Fudan Cancer Hospital. The primary outcome will be self-reported xerostomia symptoms using the Xerostomia Questionnaire (XQ). We will have information available at baseline and at 5 additional points in time (middle of radiotherapy, end of radiotherapy, and 3, 6, and 12 months after the end of radiotherapy).

Our initial analysis will be a repeated-measures analysis of XQ scores over time by acupuncture group, testing for group effects, time effects, and group by time interaction. This will be done using generalized linear mixed model regression (GLMM) (see below for further details). The primary significance level which will be used is 0.05. Our primary time endpoint is the end of radiotherapy, or 7 weeks. All three arms will be compared at this time point using an analysis of variance, and if the test is significant ($p<0.05$), we will use a Duncan's Multiple

Range Test which controls the type I comparison-wise error rate to determine which groups are different from each other.

At each point in time, we will also be able to determine if xerostomia has occurred (grade 1 or higher on the v3.0 CTCAE) and will classify each patient as either experiencing xerostomia (one or more episodes of xerostomia) or not. A secondary goal is to determine if the proportion of persons who experience xerostomia in the true acupuncture group is less than that in the sham acupuncture group or the standard care group. We will use the binomial test to test for these differences at each follow-up. We will also use the clinical response criteria of determining whether or not xerostomia has occurred or not as used by Eisbruch (22) and Pacholke (74). A significant response will be indicated if the summation XQ score≤is30. Eisbruch and colleagues revealed with bilateral radiation therapy treatment, the peak effect of XQ xerostomia scoring at 3 months post treatment is 45 +/- 10 (22). Using the criteria of ≤ 30 on the XQ score, we will use the binomial test to test for these differing proportions between groups at each follow-up. However, if 15 tests are made, in order for the results to be considered as significance as those with a 0.05 significance level, the significance level would need to be 0.003 or less (0.05/15) using a conservative Bonferroni method. We will also include a GLMM analysis to evaluate treatment and time effects upon incidence (general patterns in incidence). This analysis will test the overall treatment effect, the overall time effect and a treatment by time effect.

Other outcomes include the MDASI-HN, FACT-G, USFR, SSFR, and saliva constituents including pH, buffering capacity, and viscosity as well as levels of total protein, CGRP, and VIP. These variables are repeated measurements on multiple outcomes, possibly including hierarchical structure over subscales and related outcome measures. Desired inference includes hypotheses about treatment effects at different times and on different outcomes. Also, inference about treatment effects has to appropriately adjust for recorded baseline characteristics of the enrolled patients. With these considerations in mind, we propose the following strategy for the data analysis of these variables. As we expect scores on the criterion measures to be correlated within individuals over time, we will use GLMM. Separate sets of analyses will be conducted for each criterion variable (e.g., primary outcome: severity of self-reported xerostomia symptoms (XQ); secondary outcomes: MDASI-HN, FACT-G, saliva flow rates, and saliva constituents). For each criterion variable, we will use data across the set of post-intervention assessment points. In modeling these data, intervention condition is a between-subjects factor, time is a within-subjects factor, and baseline measures will be included as covariates.

GLMM is a flexible analytic approach with wide use in the health sciences (82). Mixed model regression allows for repeated measures across individuals by modeling the correlation among the repeated measures. GLMM can accommodate a range of correlation structures among the measures, as well as continuous and discrete outcome distributions, unbalanced designs, different link and variance functions, and, in the case of the analysis of independent data, is equivalent to logistic, ordinary least squares regression, or analysis of covariance modeling. For the GLMM, first we will assess the covariance structure of the data so that inferences about means are valid. We cannot assume that repeated measures for an individual will be equally correlated because two measurements taken at adjacent times may be more highly correlated than two measurements taken several time points apart. To select the best method for modeling the repeated measures, we will use the methods of Wolfinger (83) and statistics such as Akaike's and Schwarz's information criteria.

After determining the appropriate covariance structure, we will develop longitudinal models of the effect of intervention condition on post-intervention (follow-up) criterion measures that take into consideration the baseline values for the corresponding criterion measure as well as important covariates. Mixed model parameter estimates and tests will allow us to compare intervention effects and to examine intervention effects over the course of follow-up time points. Post-intervention follow-up criterion measures will be regressed onto study intervention condition, time of assessment, a polynomial function of time (for example, quadratic) if appropriate, and the measure of the criterion at baseline. In addition, we will add important covariates, including adaptive randomization variables and selected demographic variables, such as ethnicity and education, as well as suitable baseline health-related variables and medical characteristics. Tests of the interaction of intervention condition with time of measurement will be evaluated to determine whether the effect of intervention condition varied as a function of time. In the case of statistically significant interactions of intervention condition with time, tests of the effect of intervention condition will be conducted at each individual

time point. We will use Bonferonni corrections for each set of multiple comparisons where appropriate. Standard methods of model diagnostics will be used to identify influential observations and to determine suitable transformations where necessary.

We will compare compliance with acupuncture between patients randomized to the acupuncture and sham acupuncture groups. Compliance will be measured as the number of times the participants received their scheduled intervention per week. At each assessment after the end of radiotherapy, we will determine if patients in the two groups differ in the number of times they received their scheduled intervention (since the last analysis) using a Wilcoxon rank-sum test. We expect that these frequencies will not be normally distributed so we plan to use a nonparametric test. If there is a statistically significant difference or trend for significance in compliance between any two groups ($p<0.10$), we will also determine if there is a difference between other outcomes. We will use either t-tests or nonparametric tests such as the Wilcoxon rank-sum test to determine the significance of these differences.

We will also explore the association between expectancy and outcomes using information from the Acupuncture Expectancy Scale (AES). Expectancy will be assessed using the AES at baseline in the acupuncture and sham acupuncture groups and will be correlated with severity of xerostomia using the XQ (and other continuous variables) during and after radiotherapy. More complex analyses will also evaluate whether expectancy moderates outcomes. Following the procedures of Baron and Kenny (84), the evaluation of moderation involves examining the interaction between intervention condition and baseline expectancy. In a series of mixed model analyses, tests of the interaction of intervention condition with expectancy will be evaluated to determine whether baseline expectancy changes the impact of acupuncture condition on the outcome measures [i.e., determine whether acupuncture (active or inactive) is more effective based on baseline expectancy]. Changes in USFR, SSFR, and saliva constituents will also be examined as possible mediators of the effects of acupuncture on xerostomia symptoms following the procedures of Baron and Kenny (84) using generalized linear mixed model regression. The mediation effect estimate will be computed according to MacKinnon (85), who describes mediation as the difference of the intervention effect on outcome with and without the presence of the mediators, or alternatively, the product of the effect of the intervention on the mediators and the effect of the mediators on the outcome controlling for the intervention condition.

b. Missing data and drop-outs

Some individuals will fail to complete all questionnaires. GLMM is designed to handle this type of missing data and will give unbiased estimates of intervention effects provided that the probability of having missing data depends only on the covariates in the model. We will check this assumption by looking at predictors of missing data. We will also run analyses to examine whether study participants who drop out of the study differ from those who do not. We will examine the data with complete case analyses (intent to treat). With the use of GLMM, we will be able to include all participants in the analysis, even those with missing values for some time points and those who drop out early. Everyone who begins their intended treatment is considered to be part of the trial whether they finish it or not. The methods of GLMM in effect estimates missing values, but we will also use alternative multiple imputation methods to estimate missing values using differing assumptions if the data appear to not be missing at random.

c. Sample size calculations

These precision and power estimates are based on a final post-intervention sample size of 300 study participants, 100 in each of the three intervention groups. To achieve this post-intervention sample size, we will oversample by 20% (i.e., 375 participants) to account for attrition. That is, we will allow for up to a 20% drop-out rate.

Our primary analysis is a mixed model regression analysis with repeated measures to evaluate the between group differences across the post-intervention time points with adjustment for covariates. Assuming a two-sided significance level of 0.05, we will have at least 80% power to detect differences between any pair of group means of 0.4 standard deviation units (SD – Effect Size Index) across the three post-intervention time points. In addition, the detectable mean difference between groups is 0.49 SD units with a two-sided significance level of 0.01. In our previous study, we found differences on post-intervention XQ means ranging from 0.80 SDs at the end of radiotherapy to 0.68 SDs at 1 month when comparing the acupuncture group with

the standard care group. The associated XQ average values for the two groups at 1 month post radiotherapy were: 32.2 (SD=10.6) for acupuncture group and 41.5 (SD=9.6) for control group. As we do not know the exact differences that will occur between the sham acupuncture group and the other two groups, and given the definition of clinically significant effects (i.e., effect sizes of approximately 0.5 SDs being argued to be clinically meaningful) (25, 86, 87) the current study is still well-positioned to detect clinically significant effects between groups that may be present. We prefer to justify sample size using a paired t-test for simplicity and because it is conservative approach, although the later analyses using repeated measures will have greater power to detect differences between groups.

C.7 Limitations

There are several challenges with the current study that should be noted. There is the possibility of loss to follow-up to a greater degree than in our pilot studies due to following patients out to 1 year post radiotherapy. Several strategies will be used to decrease the chance of missing data and non-compliance. Forms will be reviewed upon receipt to ensure all items are completed, and if incomplete, participants will be contacted to determine if the question was deliberately skipped and, if not, to get a response. Scheduling of follow-up visits will also coincide with visits to the radiation oncologist, and as such, missing data will be kept at a minimum. Nevertheless, some individuals may fail to complete all questionnaires and statistical procedures noted above will be implemented. Also, although we will make every effort to recruit minority and underserved participants, as all patients will be recruited from either M. D. Anderson or Fudan Cancer Hospital, the study population will be somewhat select. Nevertheless, the sample will be representative of patients seen at the two hospitals.

C.8 Adverse Events

Acupuncture is a safe technique with few complications. The majority of reported adverse effects are best attributed to insufficient basic medical knowledge, inadequate acupuncture education, or lack of compliance with standardized practice of clean needle technique (104). Several studies by different investigators have shown that most serious adverse events are due to a lack of education or negligence on the part of the practitioner and are not due to the treatment itself (105-109). The side effects most commonly reported when acupuncture is performed correctly by properly trained personnel are relatively minor and include fainting, nausea, vomiting, bruising, and mild discomfort (105-109). In the past, patients occasionally developed contact dermatitis to components in the needles (110, 111), but this currently is rare with improved manufacturing techniques for the stainless steel needles.

For higher risk patients (i.e., those with heart disease or neutropenia), special precautions may be necessary such as avoiding the use of electrostimulation in patients with pacemakers or taking additional measures to prevent infection in patients with compromised immunity (112). Electrical stimulation will not be used in this study, and patients with evidence of infection or compromised immunity will not be included. Although the likelihood of acupuncture-induced infection in this study is low, procedures will be treated as invasive medical procedures and performed using aseptic techniques. The acupuncture needles used will be sterile and individually packaged. Reportable adverse events will include:

a. syncope
b. infection at the acupuncture site
c. allergic contact dermatitis at the acupuncture site
d. excess bleeding at the acupuncture site

C.9 Xerostomia acupuncture research at M. D. Anderson and Fudan Cancer Hospital

A single-arm pilot study completed at M. D. Anderson assessed whether radiation-induced xerostomia could be reversed using acupuncture (25). A total of 19 patients received 8 treatments over 4 weeks, with weekly assessments during treatment and then at week 5 and week 8 (1 month after treatment). Fifteen patients received IMRT, and subset analysis did not reveal any significant relationship between the amount of radiation received and response to acupuncture. The median interval from completion of radiation therapy to start of acupuncture was 21.5 months. Xerostomia scores, as assessed by the Xerostomia Inventory and the Patient Benefit Questionnaire were significantly better after acupuncture, beginning at week 2 and continuing through week 8. QOL assessed using the FACT-H&N indicated a significant difference in scores for questions related to head/neck cancer at weeks 4 and 8 (p=0.04 and p=0.006, respectively). At week 8, there was also a significant

difference in the physical well-being subscale (p=0.04) as well as in the total score (p=0.03).

For the past 3 years, we have also been conducting a randomized controlled trial at Fudan Cancer Hospital examining the effects of acupuncture on prevention of radiation-induced xerostomia. Eighty-five patients were randomized to receive either acupuncture three times a week during radiotherapy or standard care. All patients were treated following the same treatment plan, as proposed in the current study. Self-report assessments were collected weekly for the 7 weeks of radiotherapy and on week 11 (1 month after radiotherapy) and 6 months post treatment. Saliva flow using unstimulated and stimulated whole saliva flow rates was measured at baseline and weeks 1, 3, 4, 6, 7, and 11 and 6 months post acupuncture/radiotherapy. On weeks 3 and 6 the saliva tests were conducted before the acupuncture treatments.

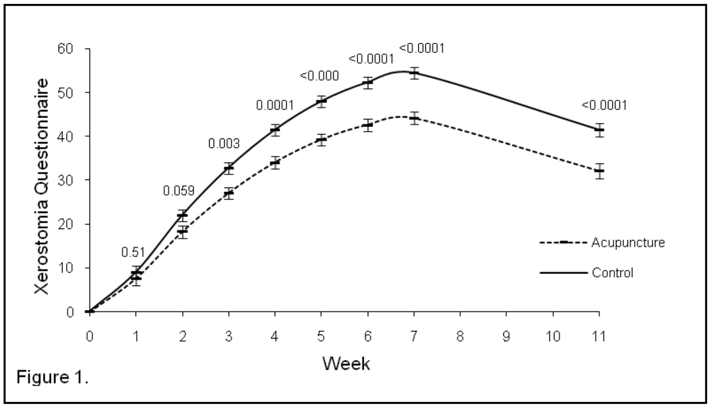

Figure 1.

As can be seen in Figure 1, starting in week 3 of treatment and lasting through the 1-month follow-up (week 11), the XQ scores were significantly lower for patients who received acupuncture (GLMM analyses, including a quadratic term for time). By the end of treatment and 1 month later, the group differences were 10 points or greater [week 7: acupuncture = 44.2 vs control = 54.5 (effect size = 0.80); week 11 acupuncture = 32.2 vs control = 41.5 (effect size = .68)]. Importantly, the mean score in the acupuncture group dropped to just above 30, which is viewed as a clinically significant response (22, 74). Examination of XQ scores of 30 or above by group using chi square analyses showed that by week 11, the acupuncture group had significantly fewer patients with scores over 30 suggesting clinically significant differences (week 11- acupuncture 60.0%; control 88.4%, P < 0.004). In addition, the effect size differences also suggest clinically significant differences (25, 86, 87). Similar findings are seen in Figures 2 and 3 for quality of life assessed with the MDASI-HN.

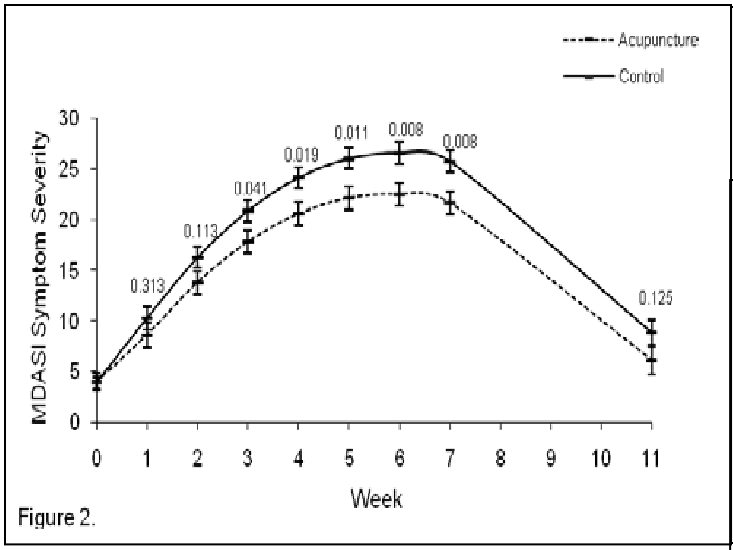

Figure 2.

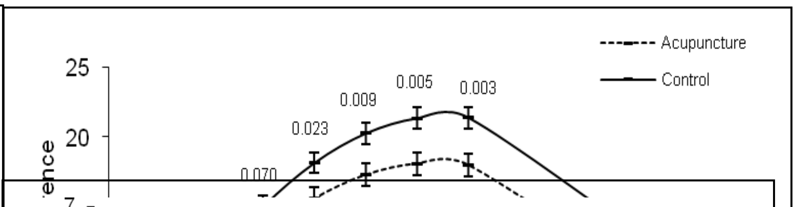

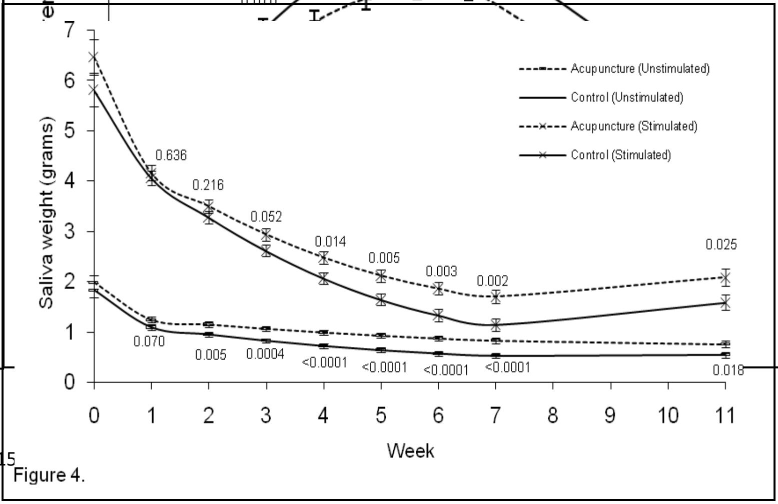

Figure 4.

Importantly, the measures of saliva flow, both stimulated and unstimulated, also indicated improvement for the acupuncture group (Figure 4). This was true even when the saliva sampling was done before the start of acupuncture treatment, as on weeks 3 and 6 (sampling done after acupuncture treatment on weeks 1, 2, 4, 5, and 7). Group differences emerged as early as week 2 and remained significant through week 11.

C.10 <u>Progress Report Since Last Review</u>

The original application was submitted in June 2009. Since then, we have completed a 6-month follow-up of 70 of the original 85 patients from the randomized trial to examine the duration of the response. Although both groups had a reduction in xerostomia symptoms, the difference in XQ scores between groups remained about the same, with lower scores in the acupuncture group (acupuncture = 21.9 vs control = 34.0, $p < 0.0006$). Examination of XQ scores of 30 or above using chi square analyses showed that by 6 months, the acupuncture group had significantly fewer patients with scores over 30 (acupuncture 27.6%; control 63.6%, $p = 0.005$). The group differences in MDASI-HN scores also remained different (MDASI-HN Symptoms: acupuncture = 2.6 vs control = 4.6, $p = 0.07$; MDASI-HN Interference: acupuncture = 2.6 vs control = 4.0, $p = 0.02$). Importantly, 6 months after the end of radiotherapy, the measure of stimulated saliva flow indicated continued improvement for the acupuncture group (acupuncture = 1.57 vs control = 0.95, $p = 0.003$). For unstimulated saliva flow, group means were in the expected direction but were not statistically significantly different (acupuncture = 0.50 vs control = 0.46, $p = 0.60$). The proposed trial will allow us to follow a larger sample size out to 12 months to determine the stability of the response.

Since this grant application was last reviewed, we also completed a small placebo/sham acupuncture trial at Fudan Cancer Hospital in order to demonstrate the feasibility and examine initial efficacy. This pilot trial used a design similar to that of the previous trial and the one outlined in the proposed study, including the inclusion/exclusion criteria (e.g., patients undergoing IMRT). Of the 30 eligible patients approached, 21 patients consented and were randomly assigned to real or sham acupuncture. The recruitment rate was similar to that in the previous study, and the research nurse remained blinded to group assignment. The XQ was collected weekly starting at baseline for the duration of radiotherapy and then again 1 month later. Salivary flow rate was collected at baseline, and weeks 1, 3, 4, 6, and then again 1 month later. Although the main purpose of this pilot trial was simply to show feasibility of doing a placebo-controlled trial, initial examination of the data suggests improved outcomes for the true versus sham acupuncture. In fact, the findings were remarkably similar to the previous trial, with the expected lower rate of xerostomia symptoms for both groups due to patients undergoing IMRT. However, there were significant group differences starting in week 3 for XQ levels (see Figure 5). The saliva flow outcomes were in the expected direction, but due to the small sample size and high variance, differences did not reach statistical significance (see Figures 6 and 7).

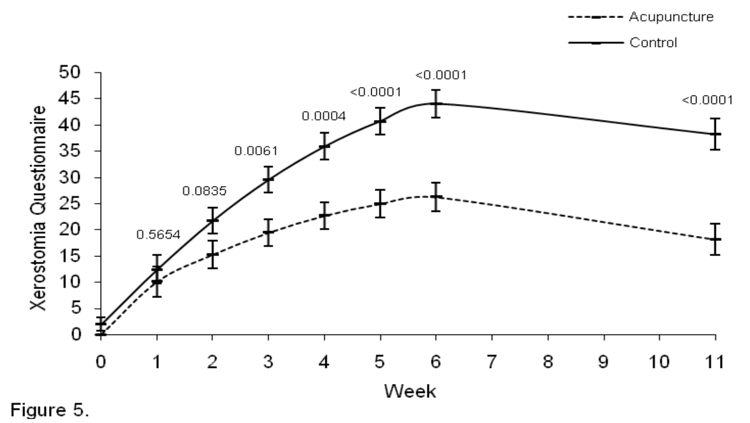

Figure 5.

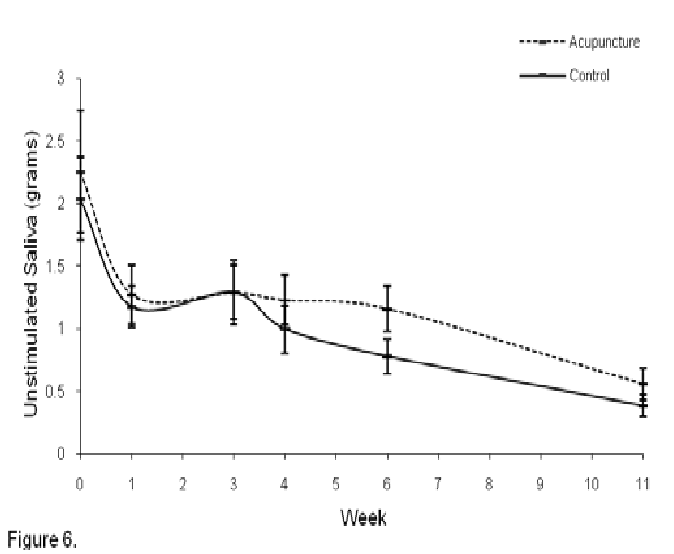

Figure 6.

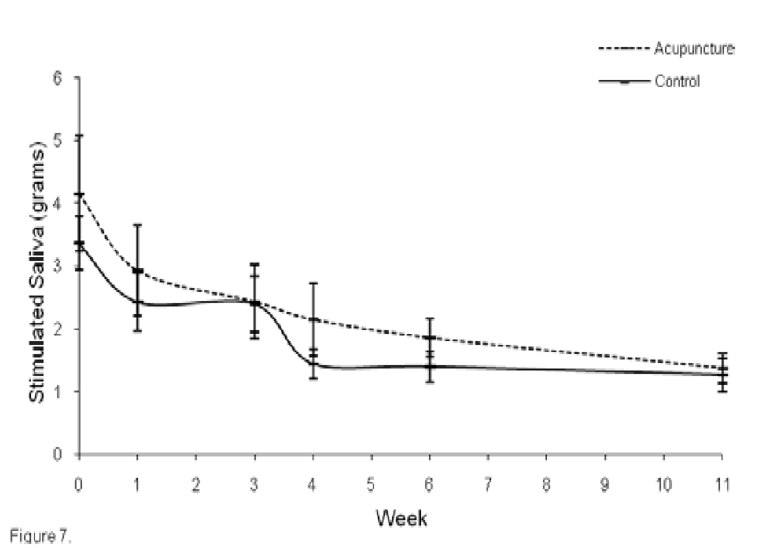

Figure 7.

The sham group remained blinded, with all patients believing they were in the active acupuncture group. Importantly, there was no indication that the sham treatment protocol had an impact on the development of xerostomia (similar outcomes to the standard care group from the previous trial, although slightly diminished XQ score due to IMRT). However, a larger trial is necessary to confirm that the placebo treatment group is in fact similar to the standard care group. Based on the positive findings on subjective (XQ) and objective (salivary flow) measures from the previous trial that compared acupuncture to standard care, it is important at this stage to conduct a large phase III clinical trial, as outlined in the proposed study, comparing true acupuncture to sham acupuncture and standard care.

In summary, our research group has had extensive experience conducting clinical trials at both M. D. Anderson and Fudan Cancer Hospital, and we have been able to implement the highest level of quality control in the oversight of the trials. Initial clinical evidence from our studies conducted at Fudan University Cancer Hospital suggests that acupuncture can prevent the development of xerostomia and reduce the symptoms of xerostomia when it develops after radiotherapy in patients with NPC. The proposed study will extend these findings by (1) ensuring the effects are not simply attributable to placebo, (2) examining the benefits for an extended period of time, and (3) conducting the trial at a second center in patients with a different type of head and neck cancer to determine the generalizability of the findings.

LITERATURE CITED

1. Emami B, Lyman J, Brown A, Coia L, Goitein M, Munzenrider JE, et al. Tolerance of normal tissue to therapeutic irradiation. Int J Radiat Oncol Biol Phys. 1991;21(1):109-22.
2. Chambers MS, Toth BB, Martin JW, Fleming TJ, Lemon JC. Oral and dental management of the cancer patient: prevention and treatment of complications. Support Care Cancer. 1995;3(3):168-75.
3. Chambers MS. Clinical commentary on prophylactic treatment of radiation-induced xerostomia. Arch Otolaryngol Head Neck Surg. 2003;129(2):251-2.
4. Yang GH. Epidemiology study of smoking of China in 1996. China Journal of Cancer. 1998;7:3-5.
5. Chang ET, Adami HO. The enigmatic epidemiology of nasopharyngeal carcinoma. Cancer Epidemiol Biomarkers Prev. 2006;15(10):1765-77.
6. Liu YL, Cao KJ, Ma GS, Deng F. Analysis of incidence and morbidity of nasopharyngeal cancer in Guangzhou. Chinese Journal of Tumor. 2008;17(7):563-4.
7. Tang ZY, Shen ZZ, Zhao S. Contemporary Oncology 2008, 11th Edition. 2008:987.
8. Jemal A, Siegel R, Ward E, Murray T, Xu J, Thun MJ. Cancer statistics, 2007. CA Cancer J Clin. 2007;57(1):43-66.
9. Fox PC, Van der Ven PF, Baum BJ, Mandel ID. Pilocarpine for the treatment of xerostomia associated with salivary gland dysfunction. Oral Surg Oral Med Oral Pathol. 1986;61(3):243-8.
10. Greenspan D, Daniels TE. Effectiveness of pilocarpine in postradiation xerostomia. Cancer. 1987;59(6):1123-5.
11. Johnson JT, Ferretti GA, Nethery WJ, Valdez IH, Fox PC, Ng D, et al. Oral pilocarpine for

postirradiation xerostomia in patients with head and neck cancer. N Engl J Med. 1993;329(6):390-5.
12. Zimmerman RP, Mark RJ, Tran LM, Juillard GF. Concomitant pilocarpine during head and neck irradiation is associated with decreased posttreatment xerostomia. Int J Radiat Oncol Biol Phys. 1997;37(3):571-5.
13. Horiot JC, Lipinski F, Schraub S, Maulard-Durdux C, Bensadoun RJ, Ardiet JM, et al. Post-radiation severe xerostomia relieved by pilocarpine: a prospective French cooperative study. Radiother Oncol. 2000;55(3):233-9.
14. Brizel DM, Wasserman TH, Henke M, Strnad V, Rudat V, Monnier A, et al. Phase III randomized trial of amifostine as a radioprotector in head and neck cancer. J Clin Oncol. 2000;18(19):3339-45.
15. Rudat V, Meyer J, Momm F, Bendel M, Henke M, Strnad V, et al. Protective effect of amifostine on dental health after radiotherapy of the head and neck. Int J Radiat Oncol Biol Phys. 2000;48(5):1339-43.
16. Wasserman T, Mackowiak JI, Brizel DM, Oster W, Zhang J, Peeples PJ, et al. Effect of amifostine on patient assessed clinical benefit in irradiated head and neck cancer. Int J Radiat Oncol Biol Phys. 2000;48(4):1035-9.
17. Lindegaard JC, Grau C. Has the outlook improved for amifostine as a clinical radioprotector? Radiother Oncol. 2000;57(2):113-8.
18. Chao KS, Majhail N, Huang CJ, Simpson JR, Perez CA, Haughey B, et al. Intensity-modulated radiation therapy reduces late salivary toxicity without compromising tumor control in patients with oropharyngeal carcinoma: a comparison with conventional techniques. Radiother Oncol. 2001;61(3):275-80.
19. Lee N, Xia P, Quivey JM, Sultanem K, Poon I, Akazawa C, et al. Intensity-modulated radiotherapy in the treatment of nasopharyngeal carcinoma: an update of the UCSF experience. Int J Radiat Oncol Biol Phys. 2002;53(1):12-22.
20. Eisbruch A, Rhodus N, Rosenthal D, Murphy B, Rasch C, Sonis S, et al. The prevention and treatment of radiotherapy - induced xerostomia. Semin Radiat Oncol. 2003;13(3):302-8.
21. Amosson CM, Teh BS, Van TJ, Uy N, Huang E, Mai WY, et al. Dosimetric predictors of xerostomia for head-and-neck cancer patients treated with the smart (simultaneous modulated accelerated radiation therapy) boost technique. Int J Radiat Oncol Biol Phys. 2003;56(1):136-44.
22. Eisbruch A, Kim HM, Terrell JE, Marsh LH, Dawson LA, Ship JA. Xerostomia and its predictors following parotid-sparing irradiation of head-and-neck cancer. Int J Radiat Oncol Biol Phys. 2001;50(3):695-704.
23. Eisbruch A, Ten Haken RK, Kim HM, Marsh LH, Ship JA. Dose, volume, and function relationships in parotid salivary glands following conformal and intensity-modulated irradiation of head and neck cancer. Int J Radiat Oncol Biol Phys. 1999;45:577-87.
24. Pow EH, Kwong DL, McMillan AS, Wong MC, Sham JS, Leung LH, et al. Xerostomia and quality of life after intensity-modulated radiotherapy vs. conventional radiotherapy for early-stage nasopharyngeal carcinoma: initial report on a randomized controlled clinical trial. Int J Radiat Oncol Biol Phys. 2006;66(4):981-91.
25. Garcia MK, Chiang JS, Cohen L, Liu M, Palmer JL, Rosenthal DI, et al. Acupuncture for radiation induced xerostomia in patients with cancer: a pilot study. Head Neck. 2009;31(10):1360-8.
26. WHO. World Health Organization: WHO Traditional Medicine Strategy 2002-2005. Geneva: World Health Organization; 2002.
27. Kaptchuk TJ. The Web That Has No Weaver: Understanding Chinese Medicine. Chicago: Congdon & Weed; 1983.
28. Mittleman E, Graynor JS. A brief overview of the analgesic and immunologic effects of acupuncture in domestic animals. JAVMA. 2000;217:1201-5.
29. A relationship between points of meridian and peripheral nerves. Acupuncture Anesthetic Theory Study: Human Anatomy Department of Shanghai Medical University. Shanghai: Shanghai Peoples' Publishing House, 1973.
30. Deng L, Gan Y, He S, Ji X, al. E. Chinese Acupuncture and Moxibustion. Cheng Y, editor. Beijing: Foreign Languages Press; 1997.
31. Cho JH, Chung WK, Kang W, Choi SM, Cho CK, Son CG. Manual acupuncture improved quality of life in cancer patients with radiation-induced xerostomia. J Altern Complement Med. 2008;14(5):523-6.
32. Meidell L, Holritz Rasmussen B. Acupuncture as an optional treatment for hospice patients with xerostomia: an intervention study. Int J Palliat Nurs. 2009;15(1):12-20.
33. Simcock R, Fallowfield L, Jenkins V. Group acupuncture to relieve radiation induced xerostomia: a

feasibility study. Acupunct Med. 2009;27(3):109-13.
34. Braga FP, Sugaya NN, Hirota SK, Weinfeld I, Magalhaes MH, Migliari DA. The effect of acupuncture on salivary flow rates in patients with radiation-induced xerostomia. Minerva Stomatol. 2008;57(7-8):343-8.
35. Johnstone PA, Peng YP, May BC, Inouye WS, Niemtzow RC. Acupuncture for pilocarpine-resistant xerostomia following radiotherapy for head and neck malignancies. Int J Radiat Oncol Biol Phys. 2001;50(2):353-7.
36. Blom M, Lundeberg T. Long-term follow-up of patients treated with acupuncture for xerostomia and the influence of additional treatment. Oral Dis. 2000;6(1):15-24.
37. Rydholm M, Strang P. Acupuncture for patients in hospital-based home care suffering from xerostomia. J Palliat Care. 1999;15(4):20-3.
38. Dawidson I, Angmar-Mansson B, Blom M, Theodorsson E, Lundeberg T. Sensory stimulation (acupuncture) increases the release of vasoactive intestinal polypeptide in the saliva of xerostomia sufferers. Neuropeptides. 1998;32(6):543-8.
39. Dawidson I, Angmar-Mansson B, Blom M, Theodorsson E, Lundeberg T. Sensory stimulation (acupuncture) increases the release of calcitonin gene-related peptide in the saliva of xerostomia sufferers. Neuropeptides. 1999;33(3):244-50.
40. Blom M, Dawidson I, Fernberg JO, Johnson G, Angmar-Mansson B. Acupuncture treatment of patients with radiation-induced xerostomia. Eur J Cancer B Oral Oncol. 1996;32B(3):182-90.
41. Blom M, Dawidson I, Angmar-Mansson B. The effect of acupuncture on salivary flow rates in patients with xerostomia. Oral Surg Oral Med Oral Pathol. 1992;73(3):293-8.
42. Brennan MT, Shariff G, Lockhart PB, Fox PC. Treatment of xerostomia: a systematic review of therapeutic trials. Dent Clin North Am. 2002;46(4):847-56.
43. Jedel E. Acupuncture in xerostomia--a systematic review. J Oral Rehabil. 2005;32(6):392-6.
44. Deng G, Hou BL, Holodny AI, Cassileth BR. Functional magnetic resonance imaging (fMRI) changes and saliva production associated with acupuncture at LI-2 acupuncture point: a randomized controlled study. BMC Complement Altern Med. 2008;8:37. PMCID: PMC2474572.
45. Johnstone PA, Niemtzow RC, Riffenburgh RH. Acupuncture for xerostomia: clinical update. Cancer. 2002;94(4):1151-6.
46. Johnstone PA, Polston GR, Niemtzow RC, Martin PJ. Integration of acupuncture into the oncology clinic. Palliat Med. 2002;16(3):235-9.
47. Pfister D, Vickers A, Deng G. Acupuncture for pain and dysfunction after neck dissection: preliminary results of a randomized controlled trial. J Support Oncol. 2008;6(6):295-6.
48. Blom M, Dawidson I, Angmar-Mansson B. Acupuncture treatment of xerostomia caused by irradiation of the head and neck region: case reports. J Oral Rehabil. 1993;20(5):491-4.
49. Blom M, Kopp S, Lundeberg T. Prognostic value of the pilocarpine test to identify patients who may obtain long-term relief from xerostomia by acupuncture treatment. Arch Otolaryngol Head Neck Surg. 1999;125(5):561-6.
50. Berman BM, Lao L, Langenberg P, Lee WL, Gilpin AM, Hochberg MC. Effectiveness of acupuncture as adjunctive therapy in osteoarthritis of the knee: a randomized, controlled trial. Ann Intern Med. 2004;141(12):901-10.
51. Furlan AD, van Tulder MW, Cherkin DC, Tsukayama H, Lao L, Koes BW, et al. Acupuncture and dryneedling for low back pain. Cochrane Database Syst Rev. 2005(1):CD001351.
52. Ezzo JM, Richardson MA, Vickers A, Allen C, Dibble SL, Issell BF, et al. Acupuncture-point stimulation for chemotherapy-induced nausea or vomiting. Cochrane Database Syst Rev. 2006(2):CD002285.
53. Lee A, Done ML. Stimulation of the wrist acupuncture point P6 for preventing postoperative nausea and vomiting. Cochrane Database Syst Rev. 2004(3):CD003281.
54. Proctor ML, Smith CA, Farquhar CM, Stones RW. Transcutaneous electrical nerve stimulation and acupuncture for primary dysmenorrhoea. Cochrane Database Syst Rev. 2002(1):CD002123.
55. Ezzo J, Berman B, Hadhazy VA, Jadad AR, Lao L, Singh BB. Is acupuncture effective for the treatment of chronic pain? A systematic review. Pain. 2000;86(3):217-25.
56. Manheimer E, White A, Berman B, Forys K, Ernst E. Meta-analysis: acupuncture for low back pain. Ann Intern Med. 2005;142(8):651-63.
57. Mao JJ. Validation of acupuncture expectancy scale in cancer patients". North American Research Conference on Complementary and Integrative Medicine [Abstract], 5/09, Minneapolis, MN. 2009.
58. Crow R, Gage H, Hampson S, Hart J, Kimber A, Thomas H. The role of expectancies in the placebo effect and their use in the delivery of health care: a systematic review. Health Technol Assess. 1999;3(3):1-96.

59. Birch S, Jamison RN. Controlled trial of Japanese acupuncture for chronic myofascial neck pain: assessment of specific and nonspecific effects of treatment. Clin J Pain. 1998;14(3):248-55.
60. Kalauokalani D, Cherkin DC, Sherman KJ, Koepsell TD, Deyo RA. Lessons from a trial of acupuncture and massage for low back pain: patient expectations and treatment effects. Spine (Phila Pa 1976). 2001;26(13):1418-24.
61. Mao JJ, Armstrong K, Farrar JT, Bowman MA. Acupuncture expectancy scale: development and preliminary validation in China. Explore (NY). 2007;3(4):372-7.
62. Diagnostic and Statistical Manual of Mental Disorders. 4th Edition. Washington, D.C.: American Psychiatric Association, 1994.
63. Pocock SJ. Clinical Trials: A practical Approach. New York: John Wiley & Sons; 1983.
64. Deadman P, Al-Khafaji M, Baker K. A Manual of Acupuncture. East Sussex, England: Journal of Chinese Medicine Publications; 1999.
65. Blom M, Lundeberg T, Dawidson I, Angmar-Mansson B. Effects on local blood flux of acupuncture stimulation used to treat xerostomia in patients suffering from Sjogren's syndrome. J Oral Rehabil. 1993;20(5):541-8.
66. Dawidson I, Angmar-Mansson B, Blom M, Theodorsson E, Lundeberg T. The influence of sensory stimulation (acupuncture) on the release of neuropeptides in the saliva of healthy subjects. Life Sci. 1998;63(8):659-74.
67. Dawidson I, Blom M, Lundeberg T, Angmar-Mansson B. The influence of acupuncture on salivary flow rates in healthy subjects. J Oral Rehabil. 1997;24(3):204-8.
68. Helms JM. Acupuncture Energetics: A Clinical Approach for Physicians. Berkeley, CA: Medical Acupuncture Publishers; 1997.
69. Park J, White A, Stevinson C, Ernst E, James M. Validating a new non-penetrating sham acupuncture device: two randomised controlled trials. Acupunct Med. 2002;20(4):168-74.
70. Park J. Sham needle control needs careful approach. Pain. 2004;109(1-2):195-6; author reply 7-9.
71. Usichenko TI, Dinse M, Hermsen M, Witstruck T, Pavlovic D, Lehmann C. Auricular acupuncture for pain relief after total hip arthroplasty - a randomized controlled study. Pain. 2005;114(3):320-7.
72. Usichenko TI, Dinse M, Lysenyuk VP, Wendt M, Pavlovic D, Lehmann C. Auricular acupuncture reduces intraoperative fentanyl requirement during hip arthroplasty--a randomized double-blinded study. Acupunct Electrother Res. 2006;31(3-4):213-21.
73. Usichenko TI, Hermsen M, Witstruck T, Hofer A, Pavlovic D, Lehmann C, et al. Auricular Acupuncture for Pain Relief after Ambulatory Knee Arthroscopy-A Pilot Study. Evid Based Complement Alternat Med. 2005;2(2):185-9.
74. Pacholke HD, Amdur RJ, Morris CG, Li JG, Dempsey JF, Hinerman RW, et al. Late xerostomia after intensity-modulated radiation therapy versus conventional radiotherapy. Am J Clin Oncol. 2005;28(4):351-8.
75. Cleeland CS, Mendoza TR, Wang XS, Chou C, Harle MT, Morrissey M, et al. Assessing symptom distress in cancer patients: the M.D. Anderson Symptom Inventory. Cancer. 2000;89(7):1634-46.
76. Chambers MS, Rosenthal DI, Weber RS. Radiation-induced xerostomia. Head Neck. 2007;29(1):58-63.
77. Cella DF, Tulsky D, Gray G, Sarafian B, Linn E, Bonomi A, et al. The Functional Assessment of Cancer Therapy Scale: Development and validation of the general measure. J Clin Oncol. 1993;11:570-9.
78. Trotti A, Colevas AD, Setser A, Rusch V, Jaques D, Budach V, et al. CTCAE v3.0: development of a comprehensive grading system for the adverse effects of cancer treatment. Semin Radiat Oncol. 2003;13(3):176-81.
79. Siqueira WL, Bermejo PR, Mustacchi Z, Nicolau J. Buffer capacity, pH, and flow rate in saliva of children aged 2-60 months with Down syndrome. Clin Oral Investig. 2005;9(1):26-9.
80. Park MS, Chang JY, Kim YY, Kang JH, Kho HS. Physical and biological properties of yam as a saliva substitute. Arch Oral Biol. 2009;55(2):177-83.
81. Bellamy JL, Cady RK, Durham PL. Salivary levels of CGRP and VIP in rhinosinusitis and migraine patients. Headache. 2006;46(1):24-33.
82. Brown H, Prescott R. Applied Mixed Models in Medicine. West Sussex, England: John Wiley and Sons, Ltd; 1999.
83. Wolfinger R. Covariance structure selection in general mixed models. Communications in Statistics; Simulation and Computation. 1993;22(4):1079-106.
84. Baron RM, Kenny DA. The moderator-mediator variable distinction in social psychology research: Conceptual, strategic, and statistical considerations. J Pers Soc Psychol. 1986;51:1173-82.

85. MacKinnon DP. Analysis of mediating variables in prevention and intervention research. NIDA Res Monogr. 1994;139:127-53.
86. Norman GR, Sloan JA, Wyrwich KW. Interpretation of changes in health-related quality of life: the remarkable universality of half a standard deviation.[see comment]. Medical Care. 2003;41(5):582-92.
87. Sloan JA, Frost MH, Berzon R, Dueck A, Guyatt G, Moinpour C, et al. The clinical significance of quality of life assessments in oncology: a summary for clinicians. Support Care Cancer. 2006;14:998.
88. MacPherson H, Thomas K, Walters S, Fitter M. A prospective survey of adverse events and treatment reactions following 34,000 consultations with professional acupuncturists. Acupunct Med 2001;19(2):93-102.
89. Lao L. Acupuncture practice, past and present: is it safe and effective? J Soc Integr Oncol 2006;4(1):13-5.
90. Lao L, Hamilton GR, Fu J, Berman BM. Is acupuncture safe? A systematic review of case reports. Altern Ther Health Med 2003;9(1):72-83.
91. Ernst G, Strzyz H, Hagmeister H. Incidence of adverse effects during acupuncture therapy-a multicentre survey. Complement Ther Med 2003;11(2):93-7.
92. Chung A, Bui L, Mills E. Adverse effects of acupuncture: Which are clinically significant? Can Fam Physician 2003;49:985-989.
93. National Acupuncture Foundation. Clean Needle Technique Manual for Acupuncturists. Washington, D.C.: National Acupuncture Foundation, 1997.
94. Filshie J. Safety aspects of acupuncture in palliative care. Acupunct Med 2001;19(2):117-22.
95. Mendoza, T.R., et al., The rapid assessment of fatigue severity in cancer patients. Cancer, 1999. 85: p. 1186-1196.
96. Glinder JG, Compas BE. Self-blame attributions in women with newly diagnosed breast cancer: A prospective study of psychological adjustment. Health Psychology. 1999;18(5):475-481.
97. Hu JC, Kwan L, Saigal CS, Litwin MS. Regret in men treated for localized prostate cancer. J Urol. Jun 2003;169(6):2279-2283.
98. Radloff, L.S., The CES-D scale: A new self-report depression scale for research in the general population. Applied Psychological Measurement, 1977. 1: p. 385-401.
99. Sherbourne, C.D. and A.L. Stewart, The Medical Outcome Study Social Support Survey. Social Science and Medicine, 1991. 32: p. 705-714.
100. Sherbourne, C.D., et al., Social support and stressful life events: Age differences in their effects on health-related quality of life among the chronically ill. Quality of Life Research, 1992. 1: p. 235-246.
101. Cleeland, C.(2006). The measurement of pain from metastatic bone disease: capturing the patient's experience. Clin Cancer Res. 12(20 Pt 2): p. 6236s-6242s.
102. Cleeland, C. (1991). Brief Pain Inventory. Pain Research Group. Houston: M. D. Anderson Cancer Center.
103. Tan, G., et al. (2004). Validation of the Brief Pain Inventory for chronic nonmalignant pain. Journal of Pain, 5(2): p. 133-7.
104. National Acupuncture Foundation. Clean Needle Technique Manual for Acupuncturists. Washington, D.C.: National Acupuncture Foundation, 1997.
105. MacPherson H, Thomas K, Walters S, Fitter M. A prospective survey of adverse events and treatment reactions following 34,000 consultations with professional acupuncturists. Acupunct Med 2001;19(2):93-102.
106. Lao L. Acupuncture practice, past and present: is it safe and effective? J Soc Integr Oncol 2006;4(1):13-5.
107. Lao L, Hamilton GR, Fu J, Berman BM. Is acupuncture safe? A systematic review of case reports. Altern Ther Health Med 2003;9(1):72-83.
108. Ernst G, Strzyz H, Hagmeister H. Incidence of adverse effects during acupuncture therapy-a multicentre survey. Complement Ther Med 2003;11(2):93-7.
109. Chung A, Bui L, Mills E. Adverse effects of acupuncture: Which are clinically significant? Can Fam Physician 2003;49:985-989.
110. Fisher AA. Allergic dermatitis from acupuncture needles. Cutis 1986;38(4):226.
111. Koizumi H, Tomoyori T, Kumakiri M, Ohkawara A. Acupuncture needle dermatitis. Contact Dermatitis 1989;21(5):352.
112. Filshie J. Safety aspects of acupuncture in palliative care. Acupunct Med 2001;19(2):117-22.

Supplement 1: Summary of Protocol Revisions

IRB Approval Date	Change	Rationale
4/30/2012	1. Removed Exclusion Criteria #10: [Patients] Participants who are taking, or who have taken any investigational new drug within the past 30 days, or who are planning to take such a drug during the course of the study.<br>2. The following text was added for study procedures:<br>**Treatment will be withheld if lab values are not within an acceptable range (ANC>500; platelet count>20K\ml; INR, 3.0); if the study participant is diagnosed with a mental incapacitation or significant emotional or psychiatric disorder that interferes with his/her ability to cooperate with study procedures; or if a participant receives any heparin or warfarin during the treatment period.**<br>**Participants will be dropped from the study, at any point during the study, if the participant begins taking a prohibited medication (Appendix F) or if the participant encounters a cerebrovascular accident or spinal cord injury.**<br>3. The “Other acupuncture” measure was added to the study evaluations.	1. We have revised our eligibility criteria #10 in order to increase participant recruitment rates.<br>2. The new text was included to clarify treatment procedures with regards to changes in blood count, mental health or exclusionary medications during treatment and study period.<br>3. Other acupuncture measure included to assess if the participant uses any acupuncture outside of the study.
8/13/2012	1. Change was made to procedures to collect necessary vital and medication data from the patient charts and/or over the phone in the event that the patient was unable to come to the MD Anderson appointment.<br>2. Added a questionnaire phone script to the evaluations	1. The change was made to be able to abstract the necessary vitals and medication from the patient charts/over the phone. To help increase patient compliance by giving more flexibility around their clinic schedules.<br>2. To help increase patient compliance by giving more flexibility around their clinic schedules. Included for use when obtaining questionnaires over the phone.
10/24/2013	1. Changes made to the sham acupuncture treatment to add a correction plan for those male participants who have a beard:<br>**The sham procedure for participants in G2 is: Sham Location 1 - placebo needle at inactive point located 0.5 cun below and 0.5 cun lateral to CV 24 on the chin (for participants who have beards this point may be omitted and indicated on treatment forms).**	1. Included as a corrective action plan on deviation log for male participants randomized to the sham group for the chin location (S1) if they have a beard.
2/28/2014	1. Change made to exclusion criteria # 10: Added the text **“certain”** beta adrenergic antagonists. Atelonol, Carvedilol and Metoprolol Succinate were eliminated of the list of prohibited medications.<br>Old Text (if applicable): Participants taking amifostine, cholinergic agonist medications (pilocarpine, cevimeline), beta adrenergic antagonists, anticholinergic agents, saliva substitutes or other medications known to affect salivary function will also be excluded.<br>New Text: Participants taking amifostine, cholinergic agonist medications (pilocarpine, cevimeline), **certain** beta adrenergic antagonists, anticholinergic agents, saliva substitutes or other medications known to affect salivary function will also be excluded	1. Change made as a clarification of prohibited meds. Atelonol, Carvedilol and Metoprolol Succinate were eliminated of the list of prohibited medications.

6/9/2014	1. Change made to procedures text on page 6 to include the analysis of salivary constituents at mid treatment:<br><br>Old Text (if applicable): All measures will be collected again during the course of radiation treatment (mid-treatment, ~~except for the assessment of salivary constituents~~), at the end of the radiation treatment period, and at 2-4, 5-9, and 10-15 months.<br><br>New Text: All measures will be collected again during the course of radiation treatment mid-treatment, at the end of the radiation treatment period, and at 2-4, 5-9, and 10-15 months.	1. The change was made to include the analysis of salivary constituents at mid treatment.
10/31/2014	1. Changes made to the overview and design text on page 4 in order to increase accrual at Fudan Cancer Hospital:<br><br>Old Text (if applicable): ~~Half the patients in each group (n=50~~) will be recruited from each center (150 patients/center) for a total sample size after attrition of 300 patients (375 patients before attrition)<br><br>New Text: **Patients** will be recruited from each center for a total sample size after attrition of 300 patients (375 patients before attrition)	1. Changed wording in order to increase accrual rate at Fudan Cancer Hospital, as MD Anderson was struggling to meet accrual goals, but overall sample size remained the same. This allowed us to complete recruitment within a reasonable timeframe.
3/4/2015	1. Change made to the sample size from 300 to 435 patients.<br>2. Changes made to sample size calculations since the study sample size went from 300 to 435. The drop-out rate went from 20% to 31%.	1. Increased accrual due to the rate of inevaluable higher than expected.<br>2. Adjustment to the sample size calculations drop-out rate to reflect changes made to the study sample size.

Supplement 1: Summary of Statistical Analysis Revisions

IRB Approval Date	Change	Rationale
1/07/2013	1. We added the following text to include a description for interim analysis:<br><br>**We will evaluate the data on a yearly basis by performing a yearly interim analysis. Our primary outcome is self-reported xerostomia symptoms using the Xerostomia Questionnaire (XQ). At each interim analysis, we will examine the difference in change scores (between baseline and the end of radiotherapy or 7 weeks) of the XQ between arms. If the true acupuncture arm is less effective than other arms and this comparison is statistically significant at <0.001, we will terminate the study early (Peto method). These interim analyses will not affect the significance level of the final analysis.**<br>**In order to address safety concerns, at each interim analysis we will also evaluate the incidence of grade 3 or higher adverse events in each arm separately. If patients in any arm experience more than 20% patients experience grade 3 or higher adverse events that are either definitely or probably related to acupuncture treatment, we will terminate that arm early.**	1. Inclusion of description for interim analyses per MD Anderson DSMB. Results of interim analyses were not shared with the investigators.

Supplement 1. Study Protocol & Statistical Analysis Plan

Final Protocol

1. SPECIFIC AIMS

Xerostomia (dry mouth) is a common problem among cancer patients who have received radiation treatment to the head and neck. Xerostomia often severely impairs quality of life (QOL), and available treatments have a low success rate. Patients suffering from radiation-induced xerostomia experience taste aberrations, dysphagia, odynophagia, difficulty sleeping and speaking, and loss of appetite. Such changes can lead to substantial nutritional deficits that can be difficult to reverse. Several pharmaceutical approaches to treatment, including saliva substitutes, chewing gum, lozenges, pilocarpine, and amifostine, have yielded limited results.

Studies conducted in the United States and Europe suggests that acupuncture is useful for treating radiation-induced xerostomia. No studies, however, have examined the use of acupuncture to reduce the severity of radiation-induced xerostomia or measured the duration of response to acupuncture treatment. Initial research conducted at The University of Texas M. D. Anderson Cancer Center (M. D. Anderson) and collaborative research at Fudan University Cancer Hospital (Fudan Cancer Hospital) in Shanghai, China, found that acupuncture can diminish symptoms in patients with xerostomia and could prevent the severity of xerostomia symptoms and improve quality of life in patients undergoing radiotherapy.

The proposed multi-center, randomized, placebo-controlled, single blind trial will examine the effectiveness of acupuncture at preventing radiation-induced xerostomia in patients undergoing radiotherapy for head and neck cancer at M. D. Anderson or nasopharyngeal carcinoma (NPC) at Fudan Cancer Hospital. Prior to receiving radiation treatment, patients will be randomized to one of three groups: Group 1 (G1) will receive true acupuncture, Group 2 (G2) will receive sham acupuncture, and Group 3 (G3) will receive standard care during the radiation treatment period. Subjective and objective measures of xerostomia will be collected prior to, during, and at the end of the radiation treatment period as well as 2-4, 5-9, and 10-15 months later, based on each patient's follow-up clinic schedule. The aims of this study are to:

PRIMARY AIM

1. Determine if true acupuncture is more effective than sham acupuncture or standard care for preventing radiation-induced xerostomia among cancer patients at M. D. Anderson and Fudan Cancer Hospital. The primary outcome will be self-reported xerostomia symptoms using the Xerostomia Questionnaire (XQ).

SECONDARY AIMS

1. Examine group differences in salivary flow rates using unstimulated and stimulated whole saliva flow rates. This will provide a more objective measure of salivary function.
2. Determine if true acupuncture is more effective than sham acupuncture or standard care for reducing the incidence of radiation-induced xerostomia.
3. Examine whether true acupuncture results in better overall quality of life than sham acupuncture or standard of care.
4. Determine acupuncture's effects on saliva-based factors including pH, buffer capacity, and viscosity as well as levels of total protein and the neuropeptides calcitonin-gene-related peptide (CGRP) and vasoactive intestinal polypeptide (VIP).
5. Examine the role of expectancy for the benefits of acupuncture in predicting outcomes. The role of expectancy as a moderator of the effects of treatment will be thoroughly examined.

2. RESEARCH STRATEGY

A. Significance

The proposed research is important for several reasons. The salivary glands are uniquely sensitive to damage from irradiation, and the development of radiation-induced xerostomia is clearly related to the exposure of salivary tissue. Mean parotid radiation doses >35Gy result in permanent xerostomia for most patients with no meaningful likelihood for recovery (1). Reduction in salivary flow begins during the first few days of treatment, with an 80% decrease noted at 6 weeks. In addition to a decrease in volume, other quantitative changes noted are a decrease in salivary pH, an increase in viscosity, and a reduction of salivary constituents (i.e., immunoglobulins, buffering capacity, small organic molecules) (2, 3).

Due to the high prevalence of smoking in China (4), head and neck cancer rates are high. In China, the incidence of nasopharyngeal cancer (NPC) is higher than in Western countries (5, 6). Available statistics indicate that the incidence of head and neck cancers in Shanghai is 14.1/100K and 6.5/100K for men and women, respectively (7). Among these, approximately 1000 are treated annually with radiation therapy at Fudan Cancer Hospital, and almost all patients receiving radiation to the head and neck area develop xerostomia. Furthermore, in the United States, nearly 40,000 patients are diagnosed annually with head and neck cancer, and approximately 80% will receive radiotherapy (8). In FY08, more than 1000 patients diagnosed with head and neck cancer were seen at M. D. Anderson and 466 underwent radiation treatment.

During the past decade, several new strategies to prevent radiation-induced xerostomia have been investigated including: 1) improved targeting of the radiation beam through intensity-modulated radiation therapy (IMRT); 2) development of radioprotective agents such as amifostine, and; 3) new surgical techniques that involve relocation of the submandibular gland to the submental space before radiation treatment. These approaches are limited by several factors, including technological constraints, primary site of disease, and proximity of the tumor and involved lymph nodes to the parotid glands. Treatment for xerostomia is, at best, palliative for symptom relief. Several approaches, including saliva substitutes, chewing gum, lozenges, pilocarpine, and amifostine, have been attempted with limited results and low acceptance due to unpleasant side effects (9-17). Also, pilocarpine is not commonly used in China, where there is currently no widely accepted treatment for radiation-induced xerostomia. Although IMRT has been shown to reduce the incidence of late grade 2 xerostomia (18, 19), all patients do not receive IMRT, and it is not commonly available in many areas of China. Also, IMRT only partly spares the parotid glands and minor salivary glands within the oral cavity; thus, the development of xerostomia remains high (20-24). In a pilot trial at M. D. Anderson investigating the use of acupuncture to treat radiation-induced xerostomia (25), most participants had received IMRT, yet these patients still benefited from the acupuncture treatment (see Preliminary Studies C.8).

Acupuncture therapy has been practiced in China and other regions for thousands of years. According to the World Health Organization, it is used in at least 78 countries worldwide (26). The therapeutic effects of acupuncture treatment range widely, and Eastern cultures commonly use it for a variety of conditions from alleviation of general fatigue to the treatment of specific diseases (27). According to traditional Chinese medicine (TCM), acupuncture points are specific sites along meridians that are related to internal organs. Modern anatomical and physiological studies have found that these acupoints are areas on the skin surface with lower electrical resistance and increased conductance compared to that of their

immediate environment (28). Some studies have shown that acupuncture points correspond to peripheral cranial and spinal nerve endings (29). To stimulate these points, the most common technique remains penetration of the skin by thin, solid, metallic needles, which are manipulated manually or by electrical stimulation.

From a TCM perspective, loss of body fluids is considered a Yin deficiency syndrome (30). Although Yin deficiencies may be related to various organ systems, xerostomia is primarily considered a deficiency of the Stomach and/or Kidney Yin. Individual patients may concomitantly exhibit signs and symptoms of Lung Yin, Heart Yin, and/or Qi-Blood deficiencies as well as any number of other underlying imbalances. Most xerostomia studies conducted to date that base acupuncture point selection on classical TCM theory have been designed to address Stomach and/or Kidney Yin deficiency and "clear heat." Heat in TCM dries body fluids and can occur in both excess and deficiency states. In xerostomia patients, excess heat is caused from the radiation and deficiency heat is caused by the underlying Yin imbalance.

A number of positive clinical trials (25, 31-41) have been conducted examining the use of acupuncture to treat radiation-induced xerostomia (See Appendix C for a Table summarizing the findings). However, two systematic reviews (42, 43) indicated that most of these trials have been small and methodological rigor is often lacking, indicating the need for larger randomized trials.

Neuronal substrates involved in changes in saliva production after stimulation of acupuncture point LI-2 have been explored by Deng and colleagues (44) using functional magnetic resonance imaging. In this randomized, sham-controlled cross-over trial, 20 healthy volunteers received both true and sham acupuncture in random order. Unilateral manual acupuncture was associated with bilateral activation of areas of the brain where gustatory, olfactory, visual stimuli and signals from expectation/suggestion are integrated. Sham acupuncture did not produce this effect. Furthermore, true acupuncture caused significantly more saliva production in participants than sham acupuncture ($p=0.02$), and there was a positive correlation ($r=0.63$) between the amount of saliva produced and changes in the regions of interest volume. This brain imaging trial, along with the other small positive studies, suggests further research is needed in this area. Finally, there needs to be a better understanding of the putative biological mechanisms involved in the treatment of xerostomia using acupuncture.

This trial seeks to improve scientific knowledge regarding the benefits of acupuncture for xerostomia by conducting a multi-center, placebo-controlled, phase III clinical trial of acupuncture to prevent xerostomia. In addition to self-report measures of xerostomia and quality of life (QOL), we will quantify changes in saliva flow and examine potential mechanisms through quantitative assessment of changes in saliva following acupuncture treatment. Although other studies (2, 3) have evaluated salivary viscosity, pH, and buffering capacity related to xerostomia, changes in these measures in response to acupuncture have not been investigated. We will also expand data from previous acupuncture studies (38, 39) reporting changes in objective indices by measuring changes in calcitonin-gene-related peptide (CGRP) and vasoactive intestinal polypeptide (VIP).

B. <u>Innovation</u>

Since the mid-1990s, studies conducted by different investigators from both the United States (25, 31, 35, 44-47) and Europe (34, 36-41, 48, 49) have shown that acupuncture can have a

positive effect on symptoms related to radiation-induced xerostomia. Patients have reported relief in as few as 5 treatments with benefits lasting up to 3 years. Only our two studies (see sections C.8 and C.9), however, have examined whether acupuncture can prevent this often-debilitating side-effect of radiation treatment from occurring or if it can diminish the symptoms once it develops. There have also been no large, definitive, phase III placebo- controlled trials to determine whether acupuncture is an effective treatment for managing radiation-induced xerostomia.

Most previous trials of acupuncture to treat xerostomia have been single-institution studies. Furthermore, few studies have examined NPC, as it is not prevalent in Western countries, which is where all the previous research on acupuncture for xerostomia has been conducted. The proposed trial is unique in that we will extend the findings from our phase II trial and pilot placebo-controlled trial conducted at Fudan Cancer Hospital by now conducting a multi-center, placebo-controlled, phase III clinical trial of acupuncture to prevent xerostomia in patients with NPC and other head and neck cancers (mainly oropharyngeal cancer). The inclusion of a placebo control group will allow us to more definitively determine the benefits of acupuncture for preventing this difficult-to-manage side effect of radiotherapy to the head and neck. Examining the effects within two different cancer populations, at two different centers, in two different countries, will increase the generalizability of our findings. Importantly, in addition to using validated self-report measures for xerostomia accepted by the Food and Drug Administration as the “gold standard” for the approval of treatments for xerostomia, we will assess more general aspects of QOL, assess objective measures of salivary flow, and quantify changes in potential mechanisms through assessment of salivary constituents.

Another innovative aspect of this study involves evaluation of participant expectations on outcomes. This will allow further exploration of how psychological phenomena may be related to outcomes in acupuncture research. Although well-conducted, placebo-controlled clinical trials have offered insights into the efficacy of acupuncture, the substantial effect seen in many placebo acupuncture groups presents a significant challenge in interpreting treatment results. Many studies, including several meta-analyses (50-56), have found that both real and placebo acupuncture produce statistically and clinically significant changes when compared to no treatment or standard/enhanced medical care. These findings suggest that the effect found in placebo acupuncture groups cannot be entirely attributed to either regression to the mean or natural disease processes; thus, the “non-specific” effect of acupuncture must be responsible in part for patients’ clinical response. Nevertheless, until we can parse the components that make up the “non-specific” effect, this effect, powerful as it may be, is likely to be discarded from a scientific standpoint. Years of psychological research has found that expectancy is one of the central pieces in this so-called “non-specific” effect. In particular, response expectancy, a form of outcome expectancy defined as “expectations held by the individual about one’s own emotional and physiological response” related to a situation or therapy, may produce important clinical changes (57). In a systematic review, Crow et al. (58) showed that when clinicians stated positive outcome expectations as opposed to uncertain expectations, most studies found improvements in patient self- reports of anxiety, pain, and distress.

A few studies have explored the association between expectancy and clinical outcomes in the context of acupuncture (59, 60). While these studies provided preliminary evidence that greater expectancy may produce better clinical response, none used a validated instrument to measure expectancy. In the proposed study, we will use an expectancy questionnaire that has been validated in both English and Mandarin (61). Investigating expectancy as part of the complex mind-body interactions of acupuncture care will likely yield important knowledge that

is both scientifically sound and clinically meaningful to patients who suffer from xerostomia and other distressing symptoms.

C. Approach

C.1 Overview and Design

The primary purpose of this trial is to investigate whether true acupuncture is more effective than sham acupuncture or standard care for preventing radiation-induced xerostomia. Prior to radiation therapy, patients will be randomized into three groups: G1 (n=100) will receive true acupuncture, G2 (n=100) will receive sham acupuncture, and G3 (n=100) will receive standard care. Patients in the acupuncture treatment groups will receive up to 21 treatments over the course of radiotherapy with no more than 3 treatments per week. The duration of acupuncture therapy in the proposed study is the same as in our pilot trials. It has been shown to have a good effect in our phase II trial and small placebo controlled trial conducted at Fudan Cancer Hospital. Furthermore, inconvenience is minimized for participants because acupuncture is provided on the same day as radiation therapy.

Potential study participants will be identified by faculty in the departments of Radiation Oncology at Fudan Cancer Hospital and M. D. Anderson and referred to the research nurse for assessment of eligibility and informed consent counseling. Potentially eligible patients will be contacted in person when they are in the clinic so that the research nurse can describe the study in detail, answer the patients' questions, and assess their interest in the study. Approximately half the patients will be recruited from each center (193 patients at MD Anderson and 242 at Fudan) for a total sample size of 435 patients (see Appendix D. Study Schema, Study Schedule, Study Timeline).

C.2 <u>Eligibility and Participants</u>

<u>Inclusion criteria</u>: Adult ≥ 18 years of age and able to give informed consent; Diagnosed with head and neck cancer, which will be primarily oropharyngeal, and scheduled to undergo IMRT with or without concurrent chemotherapy at M. D. Anderson. Treatment plan that includes external beam radiation at a mean dose of at least 24 Gy or more to one of the parotid glands (the other gland can receive less than 24 Gy); Anatomically intact parotid and submandibular glands; Karnofsky performance status > 60 (see Appendix E);

<u>Exclusion criteria</u>: History of xerostomia prior to head and neck radiation therapy or history of Sjögren's disease or another underlying systemic illness known to cause xerostomia; Prior head and neck radiation treatment; Suspected or confirmed physical closure of salivary gland ducts on either side; Known bleeding disorders or taking any dose of warfarin or heparin; Upper or lower extremity deformities that could interfere with accurate acupoint location or alter the energy pathway as defined by traditional acupuncture theory; Local skin infections at or near the acupuncture sites or active systemic infection; History of cerebrovascular accident or spinal cord injury since the mechanism of acupuncture may be associated with central nervous system activity; Mental incapacitation or significant emotional or psychiatric disorder that, in the opinion of the investigator, precludes study entry as these patients may not be able to cooperate with this slightly invasive procedure or with the data collection process; Current acknowledged use of any illicit drugs or evidence of alcohol abuse as defined by The American Psychiatric Association criteria (62); Current acknowledged use of other alternative medicines, such as herbal preparations, that could affect salivary function. If a patient is using any salivary substitute, they will be asked to refrain from using for at least 24 hours prior to saliva and questionnaire data collection. Each patient will be asked for a list of herbal supplements they are currently taking, and this will be reviewed on an individual basis.

Patients will be excluded if they are taking any herbs known or suspected to affect salivary function. Participants taking amifostine, cholinergic agonist medications (pilocarpine, cevimeline), certain beta adrenergic antagonists, anticholinergic agents, saliva substitutes or other medications known to affect salivary function will also be excluded (see Appendix F). If a patient reports use of one of the prohibited medications, they will be considered non-compliant and dropped from the study. Finally, participants will be excluded who are currently receiving acupuncture for any condition or if they have ever used acupuncture before. If a participant is admitted to the hospital for any reason during the treatment period, an in-patient order for treatment will be requested.

Participants: Over 400 patients undergo IMRT radiation for head and neck cancer each year at M. D. Anderson or one of its satellite clinics. Within the next 2 years at least two more satellite clinics will open, increasing the possible patient base. At Fudan Cancer Hospital, approximately 450 patients undergo IMRT for NPC each year. Our pilot study experience at both institutions suggests that approximately 70% of these patients will be eligible for the proposed study. Therefore, at least 500 patients will be eligible per year for a total of over 1500 patients during the 36 months designated for recruitment.

All eligible patients will be asked to participate. We expect a relatively modest acceptance rate of approximately 60%. Patients generally decline either because they do not want to be randomized or because of time constraints. This conservatively estimated patient accrual rate would permit us to recruit at least 70 patients per year at each institution. In our previous studies, the attrition rate was less than 20%; however with longer follow-up, we expect the rate will increase somewhat. Thus, with even slightly higher attrition, we will still be able to accrue at least 50 evaluable patients per year per institution, for a total of 300 evaluable patients over the approximate 3 years of recruitment (n=100 per group; n=125 per group before attrition).

C.3 Procedures

After informed consent and prior to randomization and radiation treatment, the research nurses will collect baseline measures. Patients will be informed the study is examining the effects of two different forms of acupuncture delivered during radiotherapy. This is important because in most previous sham-controlled acupuncture trials patients could accurately guess group assignment. In this trial, patients in both acupuncture groups are in fact getting real needles inserted at real acupuncture points on the body and, therefore, this is technically classified as real acupuncture. Therefore, this is not deception and, in fact, we do not actually know whether G1 will have any better outcomes than G2. In reality, we are testing two forms of acupuncture. When a patient in G2 finishes the trial, we will debrief them to let them know that they were in an acupuncture group that we were uncertain if it would work or not. We will offer them three free sessions of acupuncture after the 12 month follow-up assessment.

A modified medical examination of participants will be obtained that includes a medical history and physical; review of systems, concomitant medications, and head and neck malignancy; surgical and radiation history; and planned radiation treatment. Self-report measures and sialometry measurements, including unstimulated whole salivary flow rate (USFR) and stimulated whole salivary flow rate (SSFR), will be completed. In order to identify potential mechanisms associated with acupuncture elicited stimulation of saliva secretion, measurement of pH and buffering capacity will be performed on fresh saliva from SSFR. Samples will then be frozen for later analysis of viscosity, total protein and neuropeptides, including CGRP, and VIP. After all analyses are complete, the saliva samples will be

destroyed.

At each acupuncture visit, vital signs (blood pressure, pulse) will be taken, and medications will be reviewed prior to the acupuncture treatment. Since the SC group does not receive acupuncture, they will not have vital signs taken and medications will not be reviewed on a weekly basis. All measures will be collected again during the course of radiation treatment (mid-treatment; except the assessment of salivary constituents), at the end of the radiation treatment period, and at 2-4, 5-9, and 10-15 months. These assessments may be collected over the phone if the patient is unable to come to MD Anderson. Patients will also be asked to complete a form about their oral hygiene. All patients will be provided with standard oral care recommendations. These include keeping their mouth clean by brushing their teeth at least two times a day and preferably after each meal. They will be also encouraged to not use candies, lozenges, or drink sugary beverages. They will also be told to consume up to 1.5 liters a day of water to help diminish the effects of radiation-induced xerostomia and keep hydrated.

Follow-up assessments will be obtained 2-4, 5-9, and 10-15 months after the end of radiation treatment for a total of 6 assessments (baseline; middle of radiotherapy; end of radiotherapy; and 2-4, 5-9, and 10-15 months after the end of radiation treatment). Participants will be given a small gift certificate (100 RMB [approximately $15] value at Fudan Cancer Hospital; $35 value at M. D. Anderson) for completing each assessment. After all data are collected at the 12-month follow-up visit, patients in the G2 and G3 study arms will be offered true acupuncture treatment for free for one week (3 treatments). For the complete study schema, schedule, and timeline please see Appendix D. All data collection will be conducted by research staff who are blinded to group assignments. A procedures manual will be developed that will include all aspects of conducting the trial, including the acupuncture treatments.

Patients will be randomized by a form of adaptive randomization, called minimization, because simple randomization could result in covariate imbalances (63). Statistical adjustment of covariates can take imbalances into consideration, but results are generally more credible when they are obtained from groups with comparable baseline distributions. In minimization, group assignment is done sequentially. In this study, the patient characteristics used for group assignment will be stage of disease, age (running mean), sex, the mean planned parotid doses (left and right side will be calculated separately and balanced between groups; <10, 10-<20, 20-<26, 26-<30, 30-<35, 35-<40, 40-<50, 50-<60, ≥60), induction therapy (yes/no), and concurrent chemotherapy (yes/no). Randomization will be conducted at each site separately. This will ensure equal distribution of all factors across all groups and balanced at each site. Information on group assignment will be only communicated to the acupuncturists by the data managers to insure that the research staff remains blinded.

Treatment will be withheld if lab values are not within an acceptable range (ANC>500; platelet count>20K\ml; INR, 3.0); if the study participant is diagnosed with a mental incapacitation or significant emotional or psychiatric disorder that interferes with his/her ability to cooperate with study procedures; or if a participant receives any heparin or warfarin during the treatment period.

Participants will be dropped from the study, at any point during the study, if the participant begins taking a prohibited medication (Appendix F) or if the participant encounters a cerebrovascular accident or spinal cord injury.

C.4 Acupuncture Treatment Procedures

Treatments will be given in the acupuncture clinics at M. D. Anderson and Fudan Cancer Hospital. Patients will be placed in a supine position and, thus, blocked from seeing any of the needles. The needles will remain in place for 20 minutes with each treatment. Standardized techniques for point location will be utilized, which are based on anatomical landmarks as well as proportional measurements using the patient's own body. For example, finger breadth is based on each patient's middle finger, and the proportional unit of measure, the "cun," is defined as the distance between the two medial ends of the creases of the interphalangeal joints when the middle finger is flexed (30, 64). The acupuncture procedures will be performed using standardaseptic technique (64). The needles come in individual sterile packages, and the same level of sterility as that used for an invasive medical procedure will be applied.

All treatment providers will be licensed, experienced acupuncturists. The acupuncturist and research nurses at Fudan Cancer Hospital have been trained at M. D. Anderson. Any new acupuncturists will have one-on-one training with the lead research acupuncturist from M. D. Anderson (Dr. Kay Garcia). All acupuncturists will also participate in a video-based training program. At the end of the training period, a return demonstration of 10 treatments (5 active and 5 sham) on individuals who are not potential study subjects will be required. Finally, in order to ensure continued quality control, participating acupuncturists will be observed and evaluated three times per year. Faculty and staff from M. D. Anderson will also visit Fudan Cancer Hospital twice a year to check on the running of the trial. We are confident in the quality control procedures in place as we have been conducting collaborative research now for over 6 years, including the specifics of this protocol in two different trials at Fudan Cancer Hospital.

a. True acupuncture

Although many point combinations could be used, the investigators have attempted to identify a set of acupuncture points that integrates TCM and biomedicine, with a focus on using a minimal number of sites. The active acupuncture points for this study were selected on the basis of successful experience from our pilot studies as well as on previously published trials (35-41, 45, 46, 65-67). Points were also selected on the basis of their indications according to the classical theory of TCM (30, 64) and current understanding of the various anatomical locations and neurovascular tissues associated with each point (68).

The body acupuncture points selected for this protocol are Ren 24, Lung 7 (LU 7), and Kidney 6 (K6). One placebo needle will also be placed at Gallbladder 32 (Gb32) on the right side. This is intended to provide participants in the active treatment group with a stimulus that will not elicit De Qi sensation. Ear points selected are Shenmen, Point Zero, Salivary Gland 2' (SG 2-prime), and Larynx. Except for Ren 24, which is located in the midline and the placebo needle at Gb32, all points will be treated bilaterally. The only facial point used in this study will be Ren 24. After careful consideration and discussion, we have chosen not to use points on the face that were selected in some prior studies (i.e., Stomach 4-7). Even though problems are rare, we prefer to err on the side of precaution and avoid needling tissues that may still be friable or easily injured after radiation. Furthermore, Johnstone et al. (35, 45, 46) obtained good results without using facial points. Ren 24 is located in the midline and should not pose a problem. Patients will be excluded from the study, however, if there is any indication of skin irritation or infection at this location.

Needle insertion endpoints will be the standardized recommended depth of insertion (30, 64) or achievement of De Qi sensation per the acupuncturists' determination. De Qi is a feeling of

tingling, aching, numbness, or warmth at the acupuncture site. Acupuncturists also describe this feeling as if the muscle is grabbing the needle. Propagation of this sensation along acupuncture channels is thought to carry the therapeutic signal to target areas (30, 68). Once the standard depth of insertion or De Qi is achieved at the body points, the needles will not be manipulated further unless one becomes displaced. No electrical stimulation will be applied to the needles in this study. Acupuncture needles used for the body points will be 0.25 x 40-mm in length and for the ears 0.16 x 15-mm in length (see Appendix G for further details).

b. Sham acupuncture

Well-designed clinical acupuncture trials require a sham procedure that is indistinguishable from the real treatment, yet inactive. Although no "gold standard" has been established for placebo controls in acupuncture trials, ideally, non-penetrating needles placed at inactive points should be used. Park and colleagues validated a non-penetrating, telescoping needle with a separate device that attaches it to the skin (69, 70). This device will be used on body points in the proposed study but is too heavy and bulky for use on auricular points. For sham auricular points, Usichenko and colleagues have used points on the helix of the ear with success in 3 different studies (71-73). We propose to use a similar procedure for this study.

The sham procedure for participants in G2 is: Sham Location 1 - placebo needle at inactive point located 0.5 cun below and 0.5 cun lateral to CV 24 on the chin (for participants who have beards this point may be omitted and indicated on treatment forms); Sham Location 2 - placebo needle at inactive point located 0.5 cun radial and 0.5 cun proximal to SJ 6 between SJ and LI Channels (bilateral upper extremities); Sham Location 3 - placebo needle at inactive point located 1.0 cun below and 0.5 cun lateral to St 36, between St and Gb Channels (bilateral lower extremities). In order to elicit De Qi in the control group, one 0.25 x 40mm acupuncture needle will be used at Gb 32 above the right knee. This point is not indicated for dry mouth. Finally, four 0.16 x 15mm acupuncture needles on the helix of each ear (8 ear points) will be included. Location of inactive points will be confirmed with an electrodermal point finder.

The sham treatment will be given according to the same schedule as the true acupuncture treatment. Participants in both groups will be placed in a comfortable position. Each point will be identified and marked on the skin. A total of 14 points will be used in both groups. As the active acupuncture group is receiving treatment using a placebo needle and the placebo acupuncture group is receiving active treatment at a real acupuncture point (both at Gb 32) and with acupuncture needles inserted at inactive points on the ear, the blinding of the two groups should be excellent. In the two-arm placebo-controlled pilot study conducted at Fudan Cancer Hospital using the same two acupuncture groups (G1 and G2), perfect blinding was indicated (see C.9 below).

c. Usual care

Participants in G3 will receive standard care. This will include being provided with standard oral care recommendations. Participants in all groups will receive the same recommendations.

C.5 Evaluation

All questionnaires will be administered at the patient's clinic visits. If, however, there are time constraints, patients will be given a self-addressed stamped envelope in which to return the questionnaires. Baseline assessments will be completed within 7 days prior to starting radiotherapy, and patients will then complete the same assessments again at middle and end of radiotherapy and 2-4, 5-9, and 10-15 months after the end of radiotherapy. At middle and end of radiotherapy, data will be collected before acupuncture treatment. For patients at

Fudan Cancer Hospital, the questionnaires were forward and backward translated into Mandarin Chinese (the MDASI and FACT-G were also validated in Mandarin Chinese; see Appendix H). All of the questionnaires and saliva collection techniques have been used successfully at both institutions.

To determine if true acupuncture is more effective than sham acupuncture or standard care for reducing the incidence of radiation-induced xerostomia we will evaluate xerostomia graded according to Common Terminology Criteria for Adverse Events (CTCAE) (78) (see Appendix I).

a. <u>Xerostomia and quality of life questionnaires</u>

i. Xerostomia Questionnaire (XQ): The XQ is an 8-item questionnaire that has been validated in several cohorts (22, 74). Subjects rate each symptom on an 11-point ordinal Likert scale from 0 to 10, with higher scores indicating greater dryness or discomfort due to dryness. The item cores are added and the sum is transformed linearly to produce a final summary score ranging between 0 and 100, with higher scores representing more xerostomia. The clinical response of the XQ has been suggested by Eisbruch (22) and Pacholke research (74) with XQ scores ≤ 30 regarded as mild to no symptoms of xerostomia. This measure may also be collected over the phone if the patient is unable to come to MD Anderson.

ii. The Functional Assessment of Cancer Therapy (FACT-G): Health-related quality of life will be assessed with the FACT-G (77). This instrument is able to discriminate between individuals with metastatic and non- metastatic disease, as well as between patients at different stages of illness. The scale has been found to have good concurrent validity, high internal consistency (0.89), and good test-re-test reliability (0.82 to 0.88).

iii. M. D. Anderson Symptom Inventory for Head and Neck Cancer (MDASI-HN): Cancer-related symptoms will be assessed using the validated MDASI-HN (75, 76). The MDASI measures, on a numeric rating scale of 0-10, both the severity of symptoms and the interference symptoms cause in patients' daily activities. The 13 core MDASI symptom items are based on extensive evaluation of symptoms common to cancer and cancer treatment. The MDASI-HN includes 9 head and neck–specific items. The instrument was validated in a cohort of more than 200 patients and found to be highly reliable (76).

iv. Fatigue will be assessed using the Brief Fatigue Inventory (BFI) [95]. The BFI is a 9-item questionnaire designed to be used in the clinical setting to rapidly assess fatigue severity. The items are ranked from 0 to 10, and patients rate their fatigue at its "worst" and "usual" and as it is "now," with 0 = "no fatigue" and 10 = "fatigue as bad as you can imagine." Patients also rate how much their fatigue has interfered with their life. This single-dimension instrument was tested in a sample of 305 patients with cancer, and provided an internally stable measure of fatigue severity (0.80-0.92) [95].

v. The Brief Pain Inventory (Short Form) is a validated, widely used, self-administered questionnaire to assess severity of pain and impact of pain on daily functioning among patients (101-103). Item 1 assesses whether or not patients are currently experiencing pain other than minor headache, sprain, or toothache. Item 2 allows patients to indicate on a whole body diagram the exact location of pain. Items 3-6 measure severity of pain on a 0-10 numeric scale. Item 7 assesses current treatment and item 8 assesses relief from pain treatments or medications within the last 24 hours. Finally, item 9 includes 7 subratings

regarding the impact pain within the previous 24 hours has had on patients' QOL.

vi. Depression will be assessed using the Centers for Epidemiological Studies-Depression measures (CES-D) [98]. The CES-D is a well-validated 20-item self-report measure of depression that focuses on affective components of depression. Respondents rate the frequency of the behavior or feeling using a 4-point Likert- type scale ranging from "almost never" to "almost always." The internal consistency of the instrument is high in the general population and in patient populations. It also has demonstrated adequate convergent validity with other measures of depression [98].

vii. To thoroughly evaluate social processes, we will assess the availability of social support perceived by participants, the frequency and quality of their social interactions, and the level of expression of emotions with others. Perceived availability of social support will be measured using the Medical Outcomes Study Social Support Survey (MOS-SSS) [99], which was developed with the SF-36 quality of life index and other MOS measures in one of the largest and most comprehensive studies of health status in the chronically ill. The scale focuses on the perception of the availability of functional support. Patients rate the perceived availability of: emotional/informational support, tangible support, positive interactions, and affectional support. Internal consistency was high, ranging from 0.91 to 0.96, and test-retest reliability ranged from 0.72 to 0.76. It has good predictive validity reflecting significant relations between social support and overall health status [100]. We have also added a few questions that ask how satisfied they are with the contacts.

viii. Two items assessing behavioral and characterological self-blame for patients' cancer cause will be administered based on previous work in breast cancer patients (96). Patients will also complete two treatment regret items previously used by Hu et al. in a sample of prostate cancer patients (97). These questions will take about 1 minute to complete.

ix. Self-report measure of oral hygiene will be assessed weekly during radiotherapy, and then 2-4, 5-9, and 10-15 months after the end of radiotherapy. Patients will be asked to indicate in the past week the frequency with which they brushed their teeth, flossed their teeth, and estimate how much water they consumed on daily basis.

x. Self-report measure of other acupuncture will be assessed at the middle of radiotherapy, end of radiotherapy, and at 2-4, 5-9, and 10-15 months after the end of radiotherapy. Participants will be asked to indicate if they have received any acupuncture that is not study related as well as the frequency, indications it was received for, and any benefit experienced. If any participant has received acupuncture that is not study related, we will request contact information of the provider(s) in order to obtain the acupuncture points used in the treatment. This measure may also be collected over the phone if the patient is unable to come to MD Anderson.

b. <u>Saliva flow examination</u>

<u>Unstimulated Whole Saliva (USFR):</u> Patients are instructed to refrain from eating, drinking, and dental hygiene for a minimum of 60 minutes before saliva collection. The patient is seated upright in a quiet area where he/she will remain undisturbed and to minimize orofacial movements. The patient will relax in the designated area for 15 minutes before saliva collection. Patients are instructed not to attempt to increase or control salivation actively (such as sucking or swallowing) but to simply relax. A collection vial (including seal and cover) will be weighed on a calibrated balance with accuracy to 0.001 gm. The patient is instructed to first clear his/her mouth by swallowing. Then, with the head held slightly forward, the patient is

instructed not to talk or swallow during the 5-minute collection but to allow saliva to collect in the floor of the mouth. The 5-minute collection time is initiated on a digital timer and in view of the patient. The patient should then expectorate the accumulated saliva into the pre-weighed vial after 60 seconds. The patient should repeat this procedure 4 more times for a total collection time of 5 minutes. Patients are reminded not to swallow during the entire collection period. At the end of the 5 minutes, the collection vial is promptly sealed, weighed, placed on ice and transported to the laboratory.

Stimulated Whole Saliva (SSFR): Patients are to rest for 5 minutes prior to stimulated saliva collection. The exogenous stimulant will be a neutral chewing gum from Wrigley (unflavored gum base) used previously for SSFR. The patient will chew the gum for 3 minutes, then expectorate the gum and saliva into a disposable cup. Next, the patient should swallow to clear the mouth. The patient's saliva will then be collected in the vial for 5 minutes using a method identical to USFR collection. After a 5 minute rest period, repeat (i.e. chew gum for 3 minutes, expectorate gum and saliva into a disposable cup, swallow, then collect saliva for 5 minutes as above.)

c. Saliva constituent analyses

We will determine acupuncture's effects on saliva-based factors including pH, buffering capacity, and viscosity as well as levels of total protein, CGRP and VIP from SSFR collected at baseline, end of radiotherapy (week 7), and 2-4, 5-9, and 10-15 months later. The value of pH and buffering capacity will be measured using a portable pH meter with micro electroprobe. Buffering capacity will be measured using the method previously described by Siqueria et al. (79). Buffering capacity and pH will be conducted on fresh saliva. Viscosity, total protein, CGRP, and VIP will be measured at M. D. Anderson from frozen samples assayed in batch to decrease variance. Viscosity of the saliva (0.5 ml) will be determined using LVT Wells-Brookfield cone-and- plate digital viscometer (Brookfield Engineering Laboratory) (80). The total amount of protein in each saliva sample will be determined using the Bradford assay (Biorad protein assay kit). Levels of CGRP and VIP will be quantified using neuropeptide-specific RIA kits (Peninsula-Bachem) (81). The concentration of peptides will be normalized by the amount of protein in each sample. All assays will be conducted in the laboratory of Dr. Peiying Yang. Once the saliva samples have been assayed they will be destroyed.

d. Expectations

Acupuncture Expectancy Scale (AES): To determine the relationship between outcome expectancy related to acupuncture and clinical response, we will use the Acupuncture Expectancy Scale. This 4-item instrument was developed by Mao et al. and found to be reliable (Cronbach's α of 0.82) and valid by positive correlation with patient self-reported efficacy and satisfaction (61). The scale was further validated among cancer patients who were mostly acupuncture naïve (57). Expectancy of a benefit from acupuncture appeared to be increased by educating patients on the scientific theory and clinical evidence of acupuncture. Higher expectancy was also found in patients who had previously participated in an acupuncture trial compared with those who had not participated in an acupuncture trial (57). The scale has been translated and validated in Mandarin in China. In the proposed study, we will evaluate expectancy as a predictor of response to acupuncture. This questionnaire will be completed at baseline by all participants. Patients in the acupuncture treatment groups will complete the form again during the middle and at the end of radiotherapy.

e. Background/Exposure

Background/Exposure Questionnaire variables: The purpose of this questionnaire is to collect information on environmental and occupational exposures, phenotypic characteristics, health history, family history of cancer, and behavior patterns that could be associated with cancer of the head and neck. The data collection instrument will be comprised of the interview-based questionnaires that are currently used at MDACC. The questionnaire includes questions on the following items:

-- Demographic data, including name, address, date and place of birth, marital status, ethnicity, total years of education, and income.
-- Tobacco and alcohol exposure, including past and present smoking and drinking status, number of years smoked/drunk, and number of cigarettes smoked or drinks consumed per day.
-- Behavior patterns that could affect an individual's exposure.
-- Medical history information on previous health such as history of cancer, immune conditions, and hormone and x-ray treatment.

C.6 <u>Statistical Considerations</u>

As our first step, before analyses, we will conduct extensive descriptive analyses on the data collected at baseline and at each follow-up. Descriptive statistics (i.e., frequencies, ranges, means, proportions, standard deviations, measures of skewness and kurtosis), including 95% confidence intervals (CIs), will be computed for the measures. We will closely examine distribution characteristics of the variables using box plots, histograms, scatter plots, and the Kolmogorov-Smirnov test of normality where appropriate. Distribution assumptions will be evaluated, and if indicated, normalizing transformations or robust procedures will be used. We will evaluate bivariate associations between the outcome measures and selected demographic and disease-related variables, including age, ethnicity, time since diagnosis, and disease stage, using Pearson product-moment correlation coefficients, chi-square, or eta-squared estimates from analyses of variance where appropriate.

a. <u>Data analysis</u>

Our primary objective is to determine if true acupuncture is more effective than sham acupuncture or standard care for preventing radiation-induced xerostomia among cancer patients at M. D. Anderson and Fudan Cancer Hospital. The primary outcome will be self-reported xerostomia symptoms using the Xerostomia Questionnaire (XQ). We will have information available at baseline and at 5 additional points in time (middle of radiotherapy, end of radiotherapy, and 2-4, 5-9, and 10-15 months after the end of radiotherapy).

Our initial analysis will be a repeated-measures analysis of XQ scores over time by acupuncture group, testing for group effects, time effects, and group by time interaction. This will be done using generalized linear mixed model regression (GLMM) (see below for further details). The primary significance level which will be used is 0.05. For sample size justifications, our primary time endpoint is the end of radiotherapy, or 7 weeks. All three arms will be compared at this time point using an analysis of variance, and if the test is significant ($p<0.05$), we will use a Duncan's Multiple Range Test which controls the type I comparison-wise error rate to determine which groups are different from each other.

At each point in time, we will also be able to determine if xerostomia has occurred (grade 1 or higher on the v3.0 CTCAE) and will classify each patient as either experiencing xerostomia (one or more episodes of xerostomia) or not. A secondary goal is to determine if the proportion of persons who experience xerostomia in the true acupuncture group is less than that in the sham acupuncture group or the standard care group. We will use the

binomial test to test for these differences at each follow-up. We will also use the clinical response criteria of determining whether or not xerostomia has occurred or not as used by Eisbruch (22) and Pacholke (74). A significant response will be indicated if the summation XQ score ≤ is 30. Eisbruch and colleagues revealed with bilateral radiation therapy treatment, the peak effect of XQ xerostomia scoring at 3 months post treatment is 45 +/- 10 (22). Using the criteria of ≤ 30 on the XQ score, we will use the binomial test to test for these differing proportions between groups at each follow-up. However, if 15 tests are made, in order for the results to be considered as significance as those with a 0.05 significance level, the significance level would need to be 0.003 or less (0.05/15) using a conservative Bonferroni method. We will also include a GLMM analysis to evaluate treatment and time effects upon incidence (general patterns in incidence). This analysis will test the overall treatment effect, the overall time effect and a treatment by time effect.

Other outcomes include the MDASI-HN, FACT-G, USFR, SSFR, and saliva constituents including pH, buffering capacity, and viscosity as well as levels of total protein, CGRP, and VIP. These variables are repeated measurements on multiple outcomes, possibly including hierarchical structure over subscales and related outcome measures. Desired inference includes hypotheses about treatment effects at different times and on different outcomes. Also, inference about treatment effects has to appropriately adjust for recorded baseline characteristics of the enrolled patients. With these considerations in mind, we propose the following strategy for the data analysis of these variables. As we expect scores on the criterion measures to be correlated within individuals over time, we will use GLMM. Separate sets of analyses will be conducted for each criterion variable (e.g., primary outcome: severity of self-reported xerostomia symptoms (XQ); secondary outcomes: MDASI-HN, FACT-G, saliva flow rates, and saliva constituents). For each criterion variable, we will use data across the set of post-intervention assessment points. In modeling these data, intervention condition is a between-subjects factor, time is a within-subjects factor, and baseline measures will be included as covariates.

GLMM is a flexible analytic approach with wide use in the health sciences (82). Mixed model regression allows for repeated measures across individuals by modeling the correlation among the repeated measures. GLMM can accommodate a range of correlation structures among the measures, as well as continuous and discrete outcome distributions, unbalanced designs, different link and variance functions, and, in the case of the analysis of independent data, is equivalent to logistic, ordinary least squares regression, or analysis of covariance modeling.

For the GLMM, first we will assess the covariance structure of the data so that inferences about means are valid. We cannot assume that repeated measures for an individual will be equally correlated because two measurements taken at adjacent times may be more highly correlated than two measurements taken several time points apart. To select the best method for modeling the repeated measures, we will use the methods of Wolfinger (83) and statistics such as Akaike's and Schwarz's information criteria.

After determining the appropriate covariance structure, we will develop longitudinal models of the effect of intervention condition on post-intervention (follow-up) criterion measures that take into consideration the baseline values for the corresponding criterion measure as well as important covariates. Mixed model parameter estimates and tests will allow us to compare intervention effects and to examine intervention effects over the course of follow-up time

points. Post-intervention follow-up criterion measures will be regressed onto study intervention condition, time of assessment, a polynomial function of time (for example, quadratic) if appropriate, and the measure of the criterion at baseline. In addition, we will add important covariates, including adaptive randomization variables and selected demographic variables, such as ethnicity and education, as well as suitable baseline health-related variables and medical characteristics. Tests of the interaction of intervention condition with time of measurement will be evaluated to determine whether the effect of intervention condition varied as a function of time. In the case of statistically significant interactions of intervention condition with time, tests of the effect of intervention condition will be conducted at each individual time point. We will use Bonferonni corrections for each set of multiple comparisons where appropriate. Standard methods of model diagnostics will be used to identify influential observations and to determine suitable transformations where necessary.

We will compare compliance with acupuncture between patients randomized to the acupuncture and sham acupuncture groups. Compliance will be measured as the number of times the participants received their scheduled intervention per week. At each assessment after the end of radiotherapy, we will determine if patients in the two groups differ in the number of times they received their scheduled intervention (since the last analysis) using a Wilcoxon rank-sum test. We expect that these frequencies will not be normally distributed so we plan to use a nonparametric test. If there is a statistically significant difference or trend for significance in compliance between any two groups ($p<0.10$), we will also determine if there is a difference between other outcomes. We will use either t-tests or nonparametric tests such as the Wilcoxon rank-sum test to determine the significance of these differences.

We will also explore the association between expectancy and outcomes using information from the Acupuncture Expectancy Scale (AES). Expectancy will be assessed using the AES at baseline in the acupuncture and sham acupuncture groups and will be correlated with severity of xerostomia using the XQ (and other continuous variables) during and after radiotherapy. More complex analyses will also evaluate whether expectancy moderates outcomes. Following the procedures of Baron and Kenny (84), the evaluation of moderation involves examining the interaction between intervention condition and baseline expectancy. In a series of mixed model analyses, tests of the interaction of intervention condition with expectancy will be evaluated to determine whether baseline expectancy changes the impact of acupuncture condition on the outcome measures [i.e., determine whether acupuncture (active or inactive) is more effective based on baseline expectancy]. Changes in USFR, SSFR, and saliva constituents will also be examined as possible mediators of the effects of acupuncture on xerostomia symptoms following the procedures of Baron and Kenny (84) using generalized linear mixed model regression. The mediation effect estimate will be computed according to MacKinnon (85), who describes mediation as the difference of the intervention effect on outcome with and without the presence of the mediators, or alternatively, the product of the effect of the intervention on the mediators and the effect of the mediators on the outcome controlling for the intervention condition.

We will evaluate the data on a yearly basis by performing a yearly interim analysis. Our primary outcome is self- reported xerostomia symptoms using the Xerostomia Questionnaire (XQ). At each interim analysis, we will examine the difference in change scores (between baseline and the end of radiotherapy or 7 weeks) of the XQ between arms. If the true acupuncture arm is less effective than other arms and this comparison is statistically significant at <0.001, we will terminate the study early (Peto method). These interim analyses will not affect the significance level of the final analysis. In order to address safety concerns, at each interim analysis we will also evaluate the incidence of grade 3 or higher adverse events in each arm separately. If patients in any arm experience more than 20% patients

experience grade 3 or higher adverse events that are either definitely or probably related to acupuncture treatment, we will terminate that arm early.

b. Missing data and drop-outs

Some individuals will fail to complete all questionnaires. GLMM is designed to handle this type of missing data and will give unbiased estimates of intervention effects provided that the probability of having missing data depends only on the covariates in the model. We will check this assumption by looking at predictors of missing data. We will also run analyses to examine whether study participants who drop out of the study differ from those who do not. We will examine the data with complete case analyses (intent to treat). With the use of GLMM, we will be able to include all participants in the analysis, even those with missing values for some time points and those who drop out early. Everyone who begins their intended treatment is considered to be part of the trial whether they finish it or not. The methods of GLMM in effect estimates missing values, but we will also use alternative multiple imputation methods to estimate missing values using differing assumptions if the data appear to not be missing at random.

c. Sample size calculations

These precision and power estimates are based on a final post-intervention sample size of 300 study participants, 100 in each of the three intervention groups. To achieve this post-intervention sample size, we will oversample by 31% (i.e., 435 participants) to account for attrition. That is, we will allow for up to a 31% drop- out rate.

Our primary analysis is a mixed model regression analysis with repeated measures to evaluate the between group differences across the post-intervention time points with adjustment for covariates. Assuming a two- sided significance level of 0.05, we will have at least 80% power to detect differences between any pair of group means of 0.4 standard deviation units (SD – Effect Size Index) across the three post-intervention time points. In addition, the detectable mean difference between groups is 0.49 SD units with a two-sided significance level of 0.01. In our previous study, we found differences on post-intervention XQ means ranging from 0.80 SDs at the end of radiotherapy to 0.68 SDs at 1 month when comparing the acupuncture group with the standard care group. The associated XQ average values for the two groups at 1 month post radiotherapy were: 32.2 (SD=10.6) for acupuncture group and 41.5 (SD=9.6) for control group. As we do not know the exact differences that will occur between the sham acupuncture group and the other two groups, and given the definition of clinically significant effects (i.e., effect sizes of approximately 0.5 SDs being argued to be clinically meaningful) (25, 86, 87) the current study is still well-positioned to detect clinically significant effects between groups that may be present. We prefer to justify sample size using a paired t-test for simplicity and because it is conservative approach, although the later analyses using repeated measures will have greater power to detect differences between groups.

C.7 Limitations

There are several challenges with the current study that should be noted. There is the possibility of loss to follow-up to a greater degree than in our pilot studies due to following patients out to 1 year post radiotherapy. Several strategies will be used to decrease the chance of missing data and non-compliance. Forms will be reviewed upon receipt to ensure all items are completed, and if incomplete, participants will be contacted to determine if the question was deliberately skipped and, if not, to get a response. Scheduling of follow-up visits will also coincide with visits to the radiation oncologist, and as such, missing data will be kept at a minimum. Nevertheless, some individuals may fail to complete all questionnaires and

statistical procedures noted above will be implemented. Also, although we will make every effort to recruit minority and underserved participants, as all patients will be recruited from either M. D. Anderson or Fudan Cancer Hospital, the study population will be somewhat select. Nevertheless, the sample will be representative of patients seen at the two hospitals.

C.8 Adverse Events

Acupuncture is a safe technique with few complications. The majority of reported adverse effects are best attributed to insufficient basic medical knowledge, inadequate acupuncture education, or lack of compliance with standardized practice of clean needle technique (104). Several studies by different investigators have shown that most serious adverse events are due to a lack of education or negligence on the part of the practitioner and are not due to the treatment itself (105-109). The side effects most commonly reported when acupuncture is performed correctly by properly trained personnel are relatively minor and include fainting, nausea, vomiting, bruising, and mild discomfort (105-109). In the past, patients occasionally developed contact dermatitis to components in the needles (110, 111), but this currently is rare with improved manufacturing techniques for the stainless steel needles.

For higher risk patients (i.e., those with heart disease or neutropenia), special precautions may be necessary such as avoiding the use of electrostimulation in patients with pacemakers or taking additional measures to prevent infection in patients with compromised immunity (112). Electrical stimulation will not be used in this study, and patients with evidence of infection or compromised immunity will not be included. Although the likelihood of acupuncture-induced infection in this study is low, procedures will be treated as invasive medical procedures and performed using aseptic techniques. The acupuncture needles used will be sterile and individually packaged. Reportable adverse events will include:

a. syncope
b. infection at the acupuncture site
c. allergic contact dermatitis at the acupuncture site
d. excess bleeding at the acupuncture site

C.9 Xerostomia acupuncture research at M. D. Anderson and Fudan Cancer Hospital

A single-arm pilot study completed at M. D. Anderson assessed whether radiation-induced xerostomia could be reversed using acupuncture (25). A total of 19 patients received 8 treatments over 4 weeks, with weekly assessments during treatment and then at week 5 and week 8 (1 month after treatment). Fifteen patients received IMRT, and subset analysis did not reveal any significant relationship between the amount of radiation received and response to acupuncture. The median interval from completion of radiation therapy to start of acupuncture was 21.5 months. Xerostomia scores, as assessed by the Xerostomia Inventory and the Patient Benefit Questionnaire were significantly better after acupuncture, beginning at week 2 and continuing through week 8. QOL assessed using the FACT-H&N indicated a significant difference in scores for questions related to head/neck cancer at weeks 4 and 8 ($p=0.04$ and $p=0.006$, respectively). At week 8, there was also a significant difference in the physical well-being subscale ($p=0.04$) as well as in the total score ($p=0.03$).

For the past 3 years, we have also been conducting a randomized controlled trial at Fudan Cancer Hospital examining the effects of acupuncture on prevention of radiation-induced xerostomia. Eighty-five patients were randomized to receive either acupuncture three times a week during radiotherapy or standard care. All patients were treated following the same treatment plan, as proposed in the current study. Self-report assessments were collected weekly for the 7 weeks of radiotherapy and on week 11 (1 month after radiotherapy) and 6 months post treatment. Saliva flow using unstimulated and stimulated whole saliva flow rates

was measured at baseline and weeks 1, 3, 4, 6, 7, and 11 and 6 months post acupuncture/radiotherapy. On weeks 3 and 6 the saliva tests were conducted before the acupuncture treatments.

As can be seen in Figure 1, starting in week 3 of treatment and lasting through the 1-month follow-up (week 11), the XQ scores were significantly lower for patients who received acupuncture (GLMM analyses, including a quadratic term for time). By the end of treatment and 1 month later, the group differences were 10 points or greater [week 7: acupuncture = 44.2 vs control = 54.5 (effect size = 0.80); week 11 acupuncture = 32.2 vs control = 41.5 (effect size = 0.68)]. Importantly, the mean score in the acupuncture group dropped to just above 30, which is viewed as a clinically significant response (22, 74). Examination of XQ scores of 30 or above by group using chi square analyses showed that by week 11, the acupuncture group had significantly fewer patients with scores over 30 suggesting clinically significant differences (week 11- acupuncture 60.0%; control 88.4%, P < 0.004). In addition, the effect size differences also suggest clinically significant differences (25, 86, 87). Similar findings are seen in Figures 2 and 3 for quality of life assessed with the MDASI-HN.

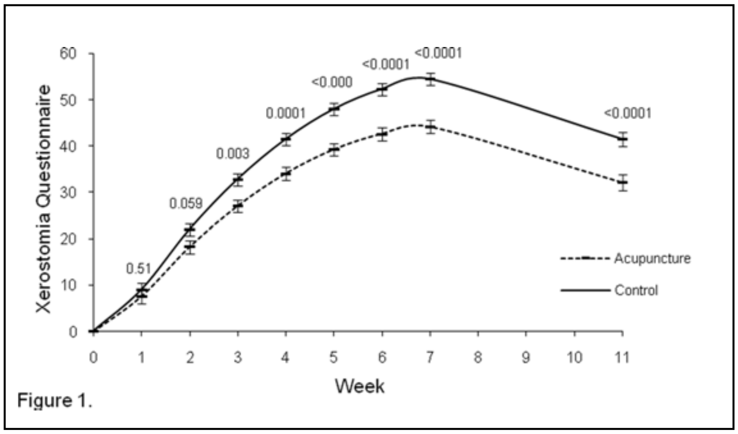

Figure 1.

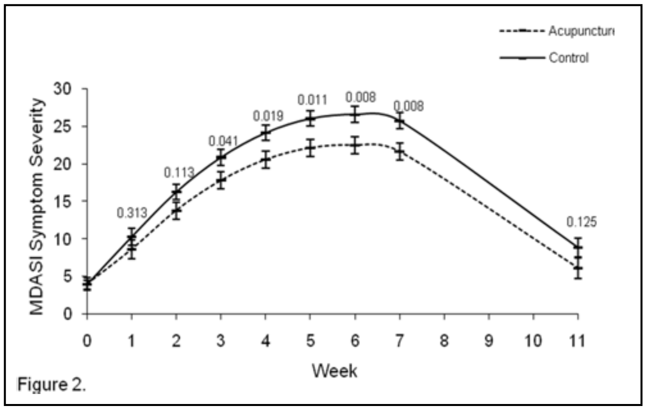

Figure 2.

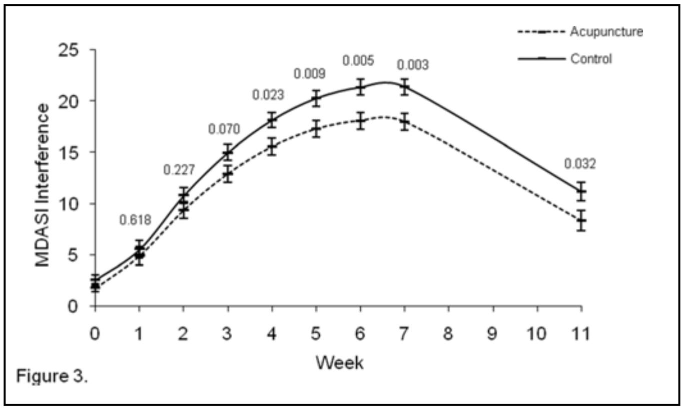

Figure 3.

Importantly, the measures of saliva flow, both stimulated and unstimulated, also indicated improvement for the acupuncture group (Figure 4). This was true even when the saliva sampling was done before the start of acupuncture treatment, as on

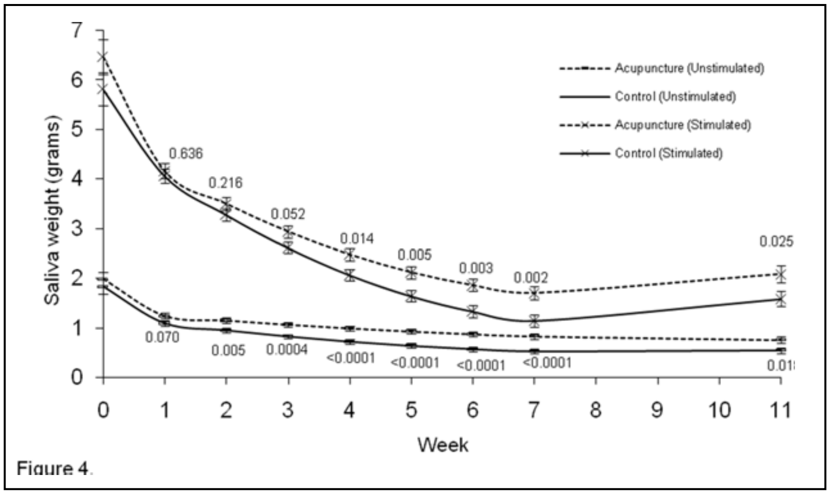

Figure 4.

weeks 3 and 6 (sampling done after acupuncture treatment on weeks 1, 2, 4, 5, and 7). Group differences emerged as early as week 2 and remained significant through week 11.

C.10 Progress Report Since Last Review

The original application was submitted in June 2009. Since then, we have completed a 6-month follow-up of 70 of the original 85 patients from the randomized trial to examine the duration of the response. Although both groups had a reduction in xerostomia symptoms, the difference in XQ scores between groups remained about the same, with lower scores in the acupuncture group (acupuncture = 21.9 vs control = 34.0, $p < 0.0006$). Examination of XQ scores of 30 or above using chi square analyses showed that by 6 months, the acupuncture group had significantly fewer patients with scores over 30 (acupuncture 27.6%; control 63.6%, $p = 0.005$). The group differences in MDASI-HN scores also remained different (MDASI-HN Symptoms: acupuncture = 2.6 vs control = 4.6, $p = 0.07$; MDASI-HN Interference: acupuncture = 2.6 vs control = 4.0, $p = 0.02$). Importantly, 6 months after the end of radiotherapy, the measure of stimulated saliva flow indicated continued improvement for the acupuncture group (acupuncture = 1.57 vs control = 0.95, $p = 0.003$). For unstimulated saliva flow, group means were in the expected direction but were not statistically significantly different (acupuncture = 0.50 vs control = 0.46, $p = 0.60$). The proposed trial will allow us to follow a larger sample size out to 12 months to determine the stability of the response.

Since this grant application was last reviewed, we also completed a small placebo/sham acupuncture trial at Fudan Cancer Hospital in order to demonstrate the feasibility and examine initial efficacy. This pilot trial used a design similar to that of the previous trial and the one outlined in the proposed study, including the inclusion/exclusion criteria (e.g., patients undergoing IMRT). Of the 30 eligible patients approached, 21 patients consented and were randomly assigned to real or sham acupuncture. The recruitment rate was similar to that in the previous study, and the research nurse remained blinded to group assignment. The XQ was collected weekly starting at baseline for the duration of radiotherapy and then again 1 month later. Salivary flow rate was collected at baseline, and weeks 1, 3, 4, 6, and then again 1 month later. Although the main purpose of this pilot trial was simply to show feasibility of doing a placebo-controlled trial, initial examination of the data suggests improved outcomes for the true versus sham acupuncture. In fact, the findings were remarkably similar to the previous trial, with the expected lower rate of xerostomia symptoms for both groups due to patients undergoing IMRT. However, there were significant group differences starting in week 3 for XQ levels (see Figure 5). The saliva flow outcomes were in the expected direction, but due to the small sample size and high variance, differences did not reach statistical significance (see Figures 6 and 7).

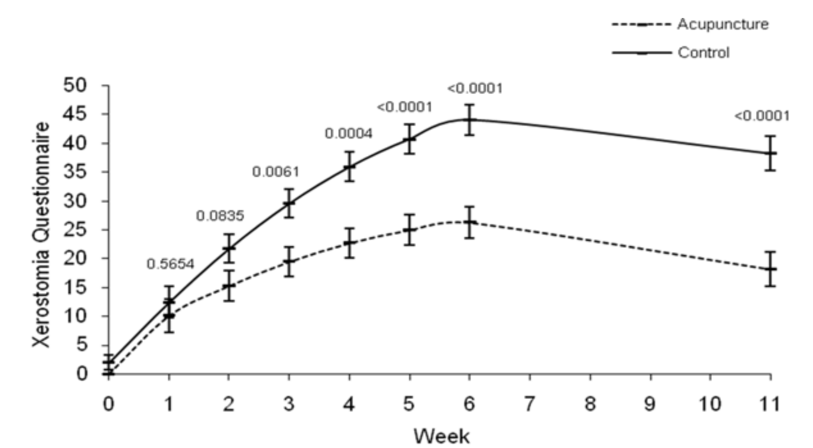

Figure 5.

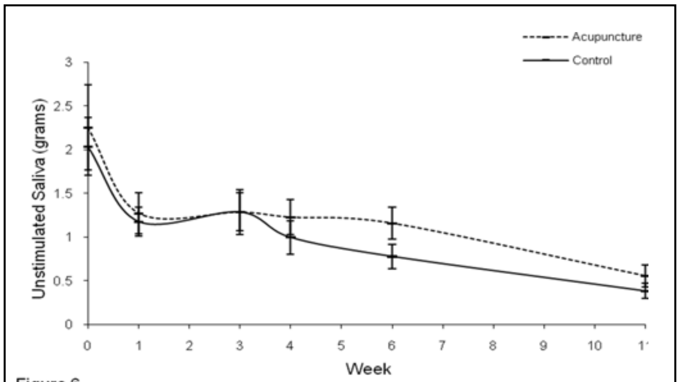

Figure 6.

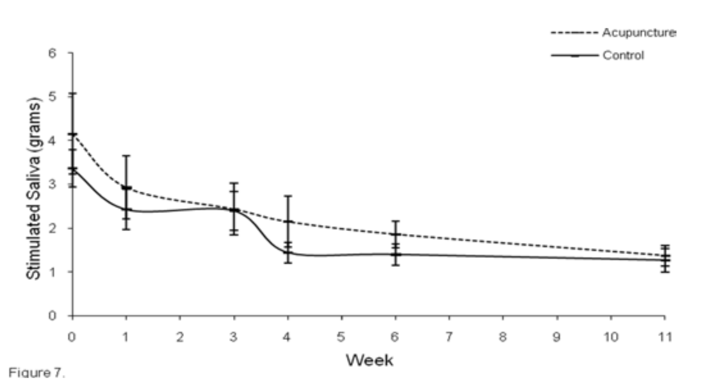

Figure 7.

The sham group remained blinded, with all patients believing they were in the active acupuncture group. Importantly, there was no indication that the sham treatment protocol had an impact on the development of xerostomia (similar outcomes to the standard care group from the previous trial, although slightly diminished XQ score due to IMRT). However, a larger trial is necessary to confirm that the placebo treatment group is in fact similar to the standard care group. Based on the positive findings on subjective (XQ) and objective (salivary flow) measures from the previous trial that compared acupuncture to standard care, it is important at this stage to conduct a large phase III clinical trial, as outlined in the proposed study, comparing true acupuncture to sham acupuncture and standard care.

In summary, our research group has had extensive experience conducting clinical trials at both M. D. Anderson and Fudan Cancer Hospital, and we have been able to implement the highest level of quality control in the oversight of the trials. Initial clinical evidence from our studies conducted at Fudan University Cancer Hospital suggests that acupuncture can prevent the development of xerostomia and reduce the symptoms of xerostomia when it develops after radiotherapy in patients with NPC. The proposed study will extend these findings by
(1) ensuring the effects are not simply attributable to placebo, (2) examining the benefits for an extended period of time, and (3) conducting the trial at a second center in patients with a different type of head and neck cancer to determine the generalizability of the findings.

LITERATURE CITED

1. Emami B, Lyman J, Brown A, Coia L, Goitein M, Munzenrider JE, et al. Tolerance of normal tissue to therapeutic irradiation. Int J Radiat Oncol Biol Phys. 1991;21(1):109-22.
2. Chambers MS, Toth BB, Martin JW, Fleming TJ, Lemon JC. Oral and dental management of the cancer patient: prevention and treatment of complications. Support Care Cancer. 1995;3(3):168-75.
3. Chambers MS. Clinical commentary on prophylactic treatment of radiation-induced xerostomia. Arch Otolaryngol Head Neck Surg. 2003;129(2):251-2.
4. Yang GH. Epidemiology study of smoking of China in 1996. China Journal of Cancer. 1998;7:3-5.
5. Chang ET, Adami HO. The enigmatic epidemiology of nasopharyngeal carcinoma. Cancer Epidemiol Biomarkers Prev. 2006;15(10):1765-77.
6. Liu YL, Cao KJ, Ma GS, Deng F. Analysis of incidence and morbidity of nasopharyngeal cancer in Guangzhou. Chinese Journal of Tumor. 2008;17(7):563-4.

7. Tang ZY, Shen ZZ, Zhao S. Contemporary Oncology 2008, 11th Edition. 2008:987.
8. Jemal A, Siegel R, Ward E, Murray T, Xu J, Thun MJ. Cancer statistics, 2007. CA Cancer J Clin. 2007;57(1):43-66.
9. Fox PC, Van der Ven PF, Baum BJ, Mandel ID. Pilocarpine for the treatment of xerostomia associated with salivary gland dysfunction. Oral Surg Oral Med Oral Pathol. 1986;61(3):243-8.
10. Greenspan D, Daniels TE. Effectiveness of pilocarpine in postradiation xerostomia. Cancer. 1987;59(6):1123-5.
11. Johnson JT, Ferretti GA, Nethery WJ, Valdez IH, Fox PC, Ng D, et al. Oral pilocarpine for postirradiation xerostomia in patients with head and neck cancer. N Engl J Med. 1993;329(6):390-5.
12. Zimmerman RP, Mark RJ, Tran LM, Juillard GF. Concomitant pilocarpine during head and neck irradiation is associated with decreased posttreatment xerostomia. Int J Radiat Oncol Biol Phys. 1997;37(3):571-5.
13. Horiot JC, Lipinski F, Schraub S, Maulard-Durdux C, Bensadoun RJ, Ardiet JM, et al. Post-radiation severe xerostomia relieved by pilocarpine: a prospective French cooperative study. Radiother Oncol. 2000;55(3):233-9.
14. Brizel DM, Wasserman TH, Henke M, Strnad V, Rudat V, Monnier A, et al. Phase III randomized trial of amifostine as a radioprotector in head and neck cancer. J Clin Oncol. 2000;18(19):3339-45.
15. Rudat V, Meyer J, Momm F, Bendel M, Henke M, Strnad V, et al. Protective effect of amifostine on dental health after radiotherapy of the head and neck. Int J Radiat Oncol Biol Phys. 2000;48(5):1339- 43.
16. Wasserman T, Mackowiak JI, Brizel DM, Oster W, Zhang J, Peeples PJ, et al. Effect of amifostine on patient assessed clinical benefit in irradiated head and neck cancer. Int J Radiat Oncol Biol Phys. 2000;48(4):1035-9.
17. Lindegaard JC, Grau C. Has the outlook improved for amifostine as a clinical radioprotector? Radiother Oncol. 2000;57(2):113-8.
18. Chao KS, Majhail N, Huang CJ, Simpson JR, Perez CA, Haughey B, et al. Intensity-modulated radiation therapy reduces late salivary toxicity without compromising tumor control in patients with oropharyngeal carcinoma: a comparison with conventional techniques. Radiother Oncol. 2001;61(3):275-80.
19. Lee N, Xia P, Quivey JM, Sultanem K, Poon I, Akazawa C, et al. Intensity-modulated radiotherapy in the treatment of nasopharyngeal carcinoma: an update of the UCSF experience. Int J Radiat Oncol Biol Phys. 2002;53(1):12-22.
20. Eisbruch A, Rhodus N, Rosenthal D, Murphy B, Rasch C, Sonis S, et al. The prevention and treatment of radiotherapy - induced xerostomia. Semin Radiat Oncol. 2003;13(3):302-8.
21. Amosson CM, Teh BS, Van TJ, Uy N, Huang E, Mai WY, et al. Dosimetric predictors of xerostomia for head-and-neck cancer patients treated with the smart (simultaneous modulated accelerated radiation therapy) boost technique. Int J Radiat Oncol Biol Phys. 2003;56(1):136-44.
22. Eisbruch A, Kim HM, Terrell JE, Marsh LH, Dawson LA, Ship JA. Xerostomia and its predictors following parotid-sparing irradiation of head-and-neck cancer. Int J Radiat Oncol Biol Phys. 2001;50(3):695-704.
23. Eisbruch A, Ten Haken RK, Kim HM, Marsh LH, Ship JA. Dose, volume, and function relationships in parotid salivary glands following conformal and intensity-modulated irradiation of head and neck cancer. Int J Radiat Oncol Biol Phys. 1999;45:577-87.
24. Pow EH, Kwong DL, McMillan AS, Wong MC, Sham JS, Leung LH, et al.

Xerostomia and quality of life after intensity-modulated radiotherapy vs. conventional radiotherapy for early-stage nasopharyngeal carcinoma: initial report on a randomized controlled clinical trial. Int J Radiat Oncol Biol Phys. 2006;66(4):981-91.
25. Garcia MK, Chiang JS, Cohen L, Liu M, Palmer JL, Rosenthal DI, et al. Acupuncture for radiation induced xerostomia in patients with cancer: a pilot study. Head Neck. 2009;31(10):1360-8.
26. WHO. World Health Organization: WHO Traditional Medicine Strategy 2002-2005. Geneva: World Health Organization; 2002.
27. Kaptchuk TJ. The Web That Has No Weaver: Understanding Chinese Medicine. Chicago: Congdon & Weed; 1983.
28. Mittleman E, Graynor JS. A brief overview of the analgesic and immunologic effects of acupuncture in domestic animals. JAVMA. 2000;217:1201-5.
29. A relationship between points of meridian and peripheral nerves. Acupuncture Anesthetic Theory Study: Human Anatomy Department of Shanghai Medical University. Shanghai: Shanghai Peoples' Publishing House, 1973.
30. Deng L, Gan Y, He S, Ji X, al. E. Chinese Acupuncture and Moxibustion. Cheng Y, editor. Beijing: Foreign Languages Press; 1997.
31. Cho JH, Chung WK, Kang W, Choi SM, Cho CK, Son CG. Manual acupuncture improved quality of life in cancer patients with radiation-induced xerostomia. J Altern Complement Med. 2008;14(5):523-6.
32. Meidell L, Holritz Rasmussen B. Acupuncture as an optional treatment for hospice patients with xerostomia: an intervention study. Int J Palliat Nurs. 2009;15(1):12-20.
33. Simcock R, Fallowfield L, Jenkins V. Group acupuncture to relieve radiation induced xerostomia: a feasibility study. Acupunct Med. 2009;27(3):109-13.
34. Braga FP, Sugaya NN, Hirota SK, Weinfeld I, Magalhaes MH, Migliari DA. The effect of acupuncture on salivary flow rates in patients with radiation-induced xerostomia. Minerva Stomatol. 2008;57(7-8):343-8.
35. Johnstone PA, Peng YP, May BC, Inouye WS, Niemtzow RC. Acupuncture for pilocarpine-resistant xerostomia following radiotherapy for head and neck malignancies. Int J Radiat Oncol Biol Phys. 2001;50(2):353-7.
36. Blom M, Lundeberg T. Long-term follow-up of patients treated with acupuncture for xerostomia and the influence of additional treatment. Oral Dis. 2000;6(1):15-24.
37. Rydholm M, Strang P. Acupuncture for patients in hospital-based home care suffering from xerostomia. J Palliat Care. 1999;15(4):20-3.
38. Dawidson I, Angmar-Mansson B, Blom M, Theodorsson E, Lundeberg T. Sensory stimulation (acupuncture) increases the release of vasoactive intestinal polypeptide in the saliva of xerostomia sufferers. Neuropeptides. 1998;32(6):543-8.
39. Dawidson I, Angmar-Mansson B, Blom M, Theodorsson E, Lundeberg T. Sensory stimulation (acupuncture) increases the release of calcitonin gene-related peptide in the saliva of xerostomia sufferers. Neuropeptides. 1999;33(3):244-50.
40. Blom M, Dawidson I, Fernberg JO, Johnson G, Angmar-Mansson B. Acupuncture treatment of patients with radiation-induced xerostomia. Eur J Cancer B Oral Oncol. 1996;32B(3):182-90.
41. Blom M, Dawidson I, Angmar-Mansson B. The effect of acupuncture on salivary flow rates in patients with xerostomia. Oral Surg Oral Med Oral Pathol. 1992;73(3):293-8.
42. Brennan MT, Shariff G, Lockhart PB, Fox PC. Treatment of xerostomia: a

systematic review of therapeutic trials. Dent Clin North Am. 2002;46(4):847-56.

43. Jedel E. Acupuncture in xerostomia--a systematic review. J Oral Rehabil. 2005;32(6):392-6.
44. Deng G, Hou BL, Holodny AI, Cassileth BR. Functional magnetic resonance imaging (fMRI) changes and saliva production associated with acupuncture at LI-2 acupuncture point: a randomized controlled study. BMC Complement Altern Med. 2008;8:37. PMCID: PMC2474572.
45. Johnstone PA, Niemtzow RC, Riffenburgh RH. Acupuncture for xerostomia: clinical update. Cancer. 2002;94(4):1151-6.
46. Johnstone PA, Polston GR, Niemtzow RC, Martin PJ. Integration of acupuncture into the oncology clinic. Palliat Med. 2002;16(3):235-9.
47. Pfister D, Vickers A, Deng G. Acupuncture for pain and dysfunction after neck dissection: preliminary results of a randomized controlled trial. J Support Oncol. 2008;6(6):295-6.
48. Blom M, Dawidson I, Angmar-Mansson B. Acupuncture treatment of xerostomia caused by irradiation of the head and neck region: case reports. J Oral Rehabil. 1993;20(5):491-4.
49. Blom M, Kopp S, Lundeberg T. Prognostic value of the pilocarpine test to identify patients who may obtain long-term relief from xerostomia by acupuncture treatment. Arch Otolaryngol Head Neck Surg. 1999;125(5):561-6.
50. Berman BM, Lao L, Langenberg P, Lee WL, Gilpin AM, Hochberg MC. Effectiveness of acupuncture as adjunctive therapy in osteoarthritis of the knee: a randomized, controlled trial. Ann Intern Med. 2004;141(12):901-10.
51. Furlan AD, van Tulder MW, Cherkin DC, Tsukayama H, Lao L, Koes BW, et al. Acupuncture and dryneedling for low back pain. Cochrane Database Syst Rev. 2005(1):CD001351.
52. Ezzo JM, Richardson MA, Vickers A, Allen C, Dibble SL, Issell BF, et al. Acupuncture-point stimulation for chemotherapy-induced nausea or vomiting. Cochrane Database Syst Rev. 2006(2):CD002285.
53. Lee A, Done ML. Stimulation of the wrist acupuncture point P6 for preventing postoperative nausea and vomiting. Cochrane Database Syst Rev. 2004(3):CD003281.
54. Proctor ML, Smith CA, Farquhar CM, Stones RW. Transcutaneous electrical nerve stimulation and acupuncture for primary dysmenorrhoea. Cochrane Database Syst Rev. 2002(1):CD002123.
55. Ezzo J, Berman B, Hadhazy VA, Jadad AR, Lao L, Singh BB. Is acupuncture effective for the treatment of chronic pain? A systematic review. Pain. 2000;86(3):217-25.
56. Manheimer E, White A, Berman B, Forys K, Ernst E. Meta-analysis: acupuncture for low back pain. Ann Intern Med. 2005;142(8):651-63.
57. Mao JJ. Validation of acupuncture expectancy scale in cancer patients”. North American Research Conference on Complementary and Integrative Medicine [Abstract], 5/09, Minneapolis, MN. 2009.
58. Crow R, Gage H, Hampson S, Hart J, Kimber A, Thomas H. The role of expectancies in the placebo effect and their use in the delivery of health care: a systematic review. Health Technol Assess. 1999;3(3):1-96.
59. Birch S, Jamison RN. Controlled trial of Japanese acupuncture for chronic myofascial neck pain: assessment of specific and nonspecific effects of treatment. Clin J Pain. 1998;14(3):248-55.

60. Kalauokalani D, Cherkin DC, Sherman KJ, Koepsell TD, Deyo RA. Lessons from a trial of acupuncture and massage for low back pain: patient expectations and treatment effects. Spine (Phila Pa 1976). 2001;26(13):1418-24.
61. Mao JJ, Armstrong K, Farrar JT, Bowman MA. Acupuncture expectancy scale: development and preliminary validation in China. Explore (NY). 2007;3(4):372-7.
62. Diagnostic and Statistical Manual of Mental Disorders. 4th Edition. Washington, D.C.: American Psychiatric Association, 1994.
63. Pocock SJ. Clinical Trials: A practical Approach. New York: John Wiley & Sons; 1983.
64. Deadman P, Al-Khafaji M, Baker K. A Manual of Acupuncture. East Sussex, England: Journal of Chinese Medicine Publications; 1999.
65. Blom M, Lundeberg T, Dawidson I, Angmar-Mansson B. Effects on local blood flux of acupuncture stimulation used to treat xerostomia in patients suffering from Sjogren's syndrome. J Oral Rehabil. 1993;20(5):541-8.
66. Dawidson I, Angmar-Mansson B, Blom M, Theodorsson E, Lundeberg T. The influence of sensory stimulation (acupuncture) on the release of neuropeptides in the saliva of healthy subjects. Life Sci. 1998;63(8):659-74.
67. Dawidson I, Blom M, Lundeberg T, Angmar-Mansson B. The influence of acupuncture on salivary flow rates in healthy subjects. J Oral Rehabil. 1997;24(3):204-8.
68. Helms JM. Acupuncture Energetics: A Clinical Approach for Physicians. Berkeley, CA: Medical Acupuncture Publishers; 1997.
69. Park J, White A, Stevinson C, Ernst E, James M. Validating a new non-penetrating sham acupuncture device: two randomised controlled trials. Acupunct Med. 2002;20(4):168-74.
70. Park J. Sham needle control needs careful approach. Pain. 2004;109(1-2):195-6; author reply 7-9.
71. Usichenko TI, Dinse M, Hermsen M, Witstruck T, Pavlovic D, Lehmann C. Auricular acupuncture for pain relief after total hip arthroplasty - a randomized controlled study. Pain. 2005;114(3):320-7.
72. Usichenko TI, Dinse M, Lysenyuk VP, Wendt M, Pavlovic D, Lehmann C. Auricular acupuncture reduces intraoperative fentanyl requirement during hip arthroplasty--a randomized double-blinded study. Acupunct Electrother Res. 2006;31(3-4):213-21.
73. Usichenko TI, Hermsen M, Witstruck T, Hofer A, Pavlovic D, Lehmann C, et al. Auricular Acupuncture for Pain Relief after Ambulatory Knee Arthroscopy-A Pilot Study. Evid Based Complement Alternat Med. 2005;2(2):185-9.
74. Pacholke HD, Amdur RJ, Morris CG, Li JG, Dempsey JF, Hinerman RW, et al. Late xerostomia after intensity-modulated radiation therapy versus conventional radiotherapy. Am J Clin Oncol. 2005;28(4):351-8.
75. Cleeland CS, Mendoza TR, Wang XS, Chou C, Harle MT, Morrissey M, et al. Assessing symptom distress in cancer patients: the M.D. Anderson Symptom Inventory. Cancer. 2000;89(7):1634-46.
76. Chambers MS, Rosenthal DI, Weber RS. Radiation-induced xerostomia. Head Neck. 2007;29(1):58-63.
77. Cella DF, Tulsky D, Gray G, Sarafian B, Linn E, Bonomi A, et al. The Functional Assessment of Cancer Therapy Scale: Development and validation of the general measure. J Clin Oncol. 1993;11:570-9.
78. Trotti A, Colevas AD, Setser A, Rusch V, Jaques D, Budach V, et al. CTCAE v3.0: development of a comprehensive grading system for the adverse effects of

cancer treatment. Semin Radiat Oncol. 2003;13(3):176-81.

79. Siqueira WL, Bermejo PR, Mustacchi Z, Nicolau J. Buffer capacity, pH, and flow rate in saliva of children aged 2-60 months with Down syndrome. Clin Oral Investig. 2005;9(1):26-9.
80. Park MS, Chang JY, Kim YY, Kang JH, Kho HS. Physical and biological properties of yam as a saliva substitute. Arch Oral Biol. 2009;55(2):177-83.
81. Bellamy JL, Cady RK, Durham PL. Salivary levels of CGRP and VIP in rhinosinusitis and migraine patients. Headache. 2006;46(1):24-33.
82. Brown H, Prescott R. Applied Mixed Models in Medicine. West Sussex, England: John Wiley and Sons, Ltd; 1999.
83. Wolfinger R. Covariance structure selection in general mixed models. Communications in Statistics; Simulation and Computation. 1993;22(4):1079-106.
84. Baron RM, Kenny DA. The moderator-mediator variable distinction insocial psychology research: Conceptual, strategic, and statistical considerations. J Pers Soc Psychol. 1986;51:1173-82.
85. MacKinnon DP. Analysis of mediating variables in prevention and intervention research. NIDA Res Monogr. 1994;139:127-53.
86. Norman GR, Sloan JA, Wyrwich KW. Interpretation of changes in health-related quality of life: the remarkable universality of half a standard deviation.[see comment]. Medical Care. 2003;41(5):582-92.
87. Sloan JA, Frost MH, Berzon R, Dueck A, Guyatt G, Moinpour C, et al. The clinical significance of quality of life assessments in oncology: a summary for clinicians. Support Care Cancer. 2006;14:998.
88. MacPherson H, Thomas K, Walters S, Fitter M. A prospective survey of adverse events and treatment reactions following 34,000 consultations with professional acupuncturists. Acupunct Med 2001;19(2):93-102.
89. Lao L. Acupuncture practice, past and present: is it safe and effective? J Soc Integr Oncol 2006;4(1):13-5.
90. Lao L, Hamilton GR, Fu J, Berman BM. Is acupuncture safe? A systematic review of case reports. Altern Ther Health Med 2003;9(1):72-83.
91. Ernst G, Strzyz H, Hagmeister H. Incidence of adverse effects during acupuncture therapy-a multicentre survey. Complement Ther Med 2003;11(2):93-7.
92. Chung A, Bui L, Mills E. Adverse effects of acupuncture: Which are clinically significant? Can Fam Physician 2003;49:985-989.
93. National Acupuncture Foundation. Clean Needle Technique Manual for Acupuncturists.Washington, D.C.: National Acupuncture Foundation, 1997.
94. Filshie J. Safety aspects of acupuncture in palliative care. Acupunct Med 2001;19(2):117-22.
95. Mendoza, T.R., et al., The rapid assessment of fatigue severity in cancer patients. Cancer, 1999. 85: p. 1186-1196.
96. Glinder JG, Compas BE. Self-blame attributions in women with newly diagnosed breast cancer: A prospective study of psychological adjustment. Health Psychology. 1999;18(5):475-481.
97. Hu JC, Kwan L, Saigal CS, Litwin MS. Regret in men treated for localized prostate cancer. J Urol. Jun 2003;169(6):2279-2283.
98. Radloff, L.S., The CES-D scale: A new self-report depression scale for research in the general population. Applied Psychological Measurement, 1977. 1: p. 385-401.

99. Sherbourne, C.D. and A.L. Stewart, The Medical Outcome Study Social Support Survey. Social Science and Medicine, 1991. 32: p. 705-714.

99. Sherbourne, C.D., et al., Social support and stressful life events: Age differences in their effects on health-related quality of life among the chronically ill. Quality of Life Research, 1992. 1: p. 235-246.

100. Cleeland, C.(2006). The measurement of pain from metastatic bone disease: capturing the patient's experience. Clin Cancer Res. 12(20 Pt 2): p. 6236s-6242s.

101. Cleeland, C. (1991). Brief Pain Inventory. Pain Research Group. Houston: M. D. Anderson Cancer Center.

102. Tan, G., et al. (2004). Validation of the Brief Pain Inventory for chronic nonmalignant pain. Journal of Pain, 5(2): p. 133-7.

103. National Acupuncture Foundation. Clean Needle Technique Manual for Acupuncturists. Washington, D.C.: National Acupuncture Foundation, 1997.

104. MacPherson H, Thomas K, Walters S, Fitter M. A prospective survey of adverse events and treatment reactions following 34,000 consultations with professional acupuncturists. Acupunct Med 2001;19(2):93-102.

105. Lao L. Acupuncture practice, past and present: is it safe and effective? J Soc Integr Oncol 2006;4(1):13-5.

106. Lao L, Hamilton GR, Fu J, Berman BM. Is acupuncture safe? A systematic review of case reports. Altern Ther Health Med 2003;9(1):72-83.

107. Ernst G, Strzyz H, Hagmeister H. Incidence of adverse effects during acupuncture therapy-a multicentre survey. Complement Ther Med 2003;11(2):93-7.

108. Chung A, Bui L, Mills E. Adverse effects of acupuncture: Which are clinically significant? Can Fam Physician 2003;49:985-989.

109. Fisher AA. Allergic dermatitis from acupuncture needles. Cutis 1986;38(4):226.

110. Koizumi H, Tomoyori T, Kumakiri M, Ohkawara A. Acupuncture needle dermatitis. Contact Dermatitis 1989;21(5):352.

111. Filshie J. Safety aspects of acupuncture in palliative care. Acupunct Med 2001;19(2):117-22.
