# Supplementary material for: Effect of True and Sham Acupuncture on Radiation-Induced Xerostomia Among Patients With Head and Neck Cancer: A Randomized Clinical Trial
Source: JAMA Netw Open. 2019 Dec 6;2(12):e1916910. doi: 10.1001/jamanetworkopen.2019.16910 (PMC6902763; doi:10.1001/jamanetworkopen.2019.16910)
Supplement: Supplement 2. — eTable 1. Inclusion and Exclusion Eligibility Criteria eTable 2. Radiation Therapy Procedures eTable 3. Acupuncture Procedures eReferences. eTable 4. Acupuncture Expectation Score by Group [file jamanetwopen-2-e1916910-s002.pdf]

Supplementary Online Content

Garcia MK, Meng Z, Rosenthal DI, et al. Effect of true and sham acupuncture on radiation-induced xerostomia among patients with head and neck cancer: a randomized clinical trial. *JAMA Netw Open*. 2019;2(12):e1916910. doi:10.1001/jamanetworkopen.2019.16910

eTable 1. Inclusion and Exclusion Eligibility Criteria

eTable 2. Radiation Therapy Procedures

eTable 3. Acupuncture Procedures

eReferences.

eTable 4. Acupuncture Expectation Score by Group

This supplementary material has been provided by the authors to give readers additional information about their work.

© 2019 Garcia MK et al. *JAMA Network Open.*

eTable 1. Inclusion and Exclusion Eligibility Criteria

Inclusion:	Exclusion:
• Adult ≥ 18 years of age.<br>• Able to give informed consent.<br>• Diagnosed with head and neck squamous cell carcinoma (primarily oropharyngeal or nasopharyngeal).<br>• Planned intensity-modulated radiation therapy (IMRT), with or without concurrent chemotherapy, at a mean dose of at least 24 Gy to at least one of the parotid glands. (The other gland could receive any dose level).<br>• Anatomically intact parotid and submandibular glands.<br>• Karnofsky performance status > 60.	• History of xerostomia prior to head and neck radiation therapy, Sjögren's disease, or another underlying systemic illness known to cause xerostomia.<br>• Prior head and neck radiation treatment.<br>• Suspected or confirmed physical closure of salivary gland ducts on either side.<br>• Known bleeding disorders or taking any dose of warfarin or heparin.<br>• Upper or lower extremity deformities that could interfere with accurate acupoint location or alter the energy pathway as defined by traditional acupuncture theory.<br>• Local skin infections at or near the acupuncture sites or active systemic infection.<br>• History of cerebrovascular accident or spinal cord injury.<br>• Mental incapacitation or significant emotional or psychiatric disorder that, in the opinion of the investigator, may prevent the patient from cooperating with the slightly invasive procedures or the data collection process.<br>• Current use of any illicit drugs or evidence of alcohol abuse as defined by the American Psychiatric Association.<br>• Current use of alternative medicines, such as herbal (traditional Chinese medicine) preparations, that could affect salivary function. (If a patient was using any salivary substitute, they were asked to refrain from using the product for at least 24 hours prior to saliva and questionnaire data collection.)<br>• Taking amifostine, cholinergic agonist medications (pilocarpine, cevimeline), certain beta-adrenergic antagonists, anticholinergic agents, or other medications known to affect salivary function.<br>• Prior acupuncture treatment.

© 2019 Garcia MK et al. *JAMA Network Open.*

eTable 2. Radiation Therapy Procedures

- Patients were immobilized in the supine treatment position by a thermoplastic head and shoulder device.
- Clinical target simulation was performed.
- Gross tumor volume (GTV) included the primary tumor and metastatic lymph nodes.
- Clinical target volume (CTV) of gross tumor (CTV-G) included the nasopharynx, retropharyngeal lymph node, anterior one-third of the clivus, skull base, the inferior sphenoid sinus, pterygoid fossa, parapharyngeal space, posterior one-third of the nasal cavity, and the maxillary sinus (the whole sphenoid sinus and clivus covered for stage T3 and T4).
- CTV of the neck nodal regions (CTV-N) in N1–3 disease patients included level II, III, IV, and V.
- Patients with negative neck node only received prophylactic irradiation to the upper neck lymph drainage region, including levels II, III and VA.
- Neck level Ib was excluded in CTV-N in patients without involved node(s) in level Ib in China, but level Ib was included in Houston if level IIa or the anterior nasal cavity were involved.
- Planning target volume (PTV) of gross tumor (PTV-G) of CTV-G and PTV of neck nodal regions (PTV-N) of CTV-N encompassed the CTV plus a 2- or 3-mm margin with fine adjustment.
- All critical adjacent structures including parotid glands and submandibular glands, except for the brainstem, spinal cord, temporal lobe, eyeballs, lens, optic nerves, chiasm, and larynx were segmented. Inverse planning software (ADAC Pinnacle 7.4 or 7.6) was used for plan optimization.
- Planning goals included mean doses of 26 Gy to the parotid of the involved neck side, 16 Gy to the uninvolved neck side, and less than 30 Gy to 50% volume of the parotid glands.
- Prescription doses were 66–70.4 Gy in 30–32 fractions to the GTV of nasopharynx, 66-70 Gy to the positive neck nodes, 60 Gy to the high-risk clinical target volume, and 54 Gy to the low-risk clinical target volume.
- All patients were irradiated 1 fraction daily, 5 days per week. Daily CT or kV image verification was performed.

© 2019 Garcia MK et al. *JAMA Network Open.*

eTable 3. Acupuncture Procedures

Acupuncture Treatment Procedures

Treatments were given in the acupuncture clinics at M. D. Anderson and Fudan Cancer Hospital. Patients were placed in a supine position and, thus, blocked from seeing the needles. The needles remained in place for 20 minutes with each treatment. Standardized techniques for point location were utilized and based on anatomical landmarks as well as proportional measurements using the patient's own body. For example, finger breadth is based on each patient's middle finger, and the proportional unit of measure, the "cun," is defined as the distance between the two medial ends of the creases of the interphalangeal joints when the middle finger is flexed. [1,2] The acupuncture procedures will be performed using standard aseptic technique. [3] The needles came in individual sterile packages, and the same level of sterility as that used for an invasive medical procedure was applied.

All treatment providers were licensed, experienced acupuncturists. The acupuncturists and research nurses at Fudan Cancer Hospital were trained at M. D. Anderson. All acupuncturists participated in a video-based training program. At the end of the training period, a return demonstration of 10 treatments (5 active and 5 sham) on individuals who are not potential study subjects was required. Finally, in order to ensure continued quality control, participating acupuncturists were evaluated 3 times per year for the first year and 1-2 times per year in subsequent years. Faculty and staff from M. D. Anderson visited Fudan Cancer Hospital twice a year to check on the running of the trial.

a. True acupuncture

Although many point combinations could be used, the investigators attempted to identify a set of acupuncture points that integrated TCM and biomedicine, with a focus on using a minimal number of sites. The active acupuncture points for this study were selected on the basis of successful experience from our pilot studies as well as on previously published trials [4-15]. Points were also selected on the basis of their indications according to the classical theory of TCM [1,2] and current understanding of the various anatomical locations and neurovascular tissues associated with each point. [16]

The body acupuncture points selected for this protocol were Ren 24, Lung 7 (LU 7), and Kidney 6 (K6). One placebo needle was placed at Gallbladder 32 (Gb32) on the right side. This was intended to provide participants in the active treatment group with a stimulus that did not elicit De Qi sensation. Ear points selected were Shenmen, Point Zero, Salivary Gland 2' (SG 2-prime), and Larynx. Except for Ren 24, which is located in the midline and the placebo needle at Gb32, all points will be treated bilaterally. The only facial point used in this study was Ren 24. We chose not to use points on the face that were selected in some prior studies (i.e., Stomach 4-7). Even though problems are rare, we preferred to err on the side of precaution and avoid needling tissues that could still be friable or easily injured after radiation. Furthermore, Johnstone et al. [4,12,13] obtained good results without using facial points. Ren 24 is located in the midline and did not pose a problem. Patients were excluded from the study, if there was any indication of skin irritation or infection at this location.

Needle insertion endpoints were standardized recommended depths of insertion [1,2] or achievement of De Qi sensation per the acupuncturists' determination. Once the standard depth of insertion or De Qi was achieved at the body points, the needles were not manipulated further unless one became displaced. No electrical stimulation was applied to the needles. Acupuncture needles used for body points were 0.25 x 40-mm in length and for the ears 0.16 x 15-mm in length.

© 2019 Garcia MK et al. *JAMA Network Open.*

b. Sham acupuncture

Non-penetrating needles with the Park device [17,18] were placed at inactive points as follows: Sham Location 1 - placebo needle at inactive point located 0.5 cun below and 0.5 cun lateral to CV 24 on the chin (for participants with beards this point was omitted and indicated on treatment forms); Sham Location 2 - placebo needle at inactive point located 0.5 cun radial and 0.5 cun proximal to SJ 6 between SJ and LI Channels (bilateral upper extremities); Sham Location 3 - placebo needle at inactive point located 1.0 cun below and 0.5 cun lateral to St 36, between St and Gb Channels (bilateral lower extremities). In order to elicit De Qi in the control group, one 0.25 x 40mm acupuncture needle was used at Gb 32 above the right knee. This point is not indicated for dry mouth. Finally, four 0.16 x 15mm acupuncture needles on the helix of each ear (8 ear points) was included.

The sham treatment was given according to the same schedule as the true acupuncture treatment. A total of 14 points was used in both groups. As the active acupuncture group received treatment using a placebo needle and the placebo acupuncture group received active treatment at a real acupuncture point (both at Gb 32) and with acupuncture needles inserted at inactive points on the ear, the blinding of the two groups was maintained.

The following was documented at each visit:

G1

Treatment Duration	Points	Depth		Sens. of De Qi				Comments
				L		R		
				Yes (0)	No (1)	Yes (0)	No (1)	
Start:	CV24	mid						
	Lu7	L	R					
	K6	L	R					
	Gb 32-Park							
	Shenmen	L	R					
Stop:	Point 0	L	R					
	SG 2 (prime)	L	R					
	Larynx	L	R					

G2

Treatment Duration	Points	Depth		Sens. of De Qi				Comments
				L		R		
				Yes (0)	No (1)	Yes (0)	No (1)	
Start:	S1 - Chin							
	S2 - BUE							
	S3 - BLE							
	Gb 32 - Seirin	R						
Stop:	Helix 1	L	R					
	Helix 2	L	R					
	Helix 3	L	R					
	Helix 4	L	R					

© 2019 Garcia MK et al. *JAMA Network Open.*

eReferences

1. Deng LY. *Chinese Acupuncture and Moxibustion.* Beijing: Foreign Languages Press; 1997.
2. Dedman P, Al-Khafaji M, Baker K. *A Manual of Acupuncture.* East Sussex, UK: Journal of Chinese Medicine Publications; 1998.
3. National Acupuncture Foundation. Clean Needle Technique Manual for Acupuncturists. Washington, D.C.: National Acupuncture Foundation, 1997.
4. Johnstone PA, Peng YP, May BC, Inouye WS, Niemtzow RC. Acupuncture for pilocarpine-resistant xerostomia following radiotherapy for head and neck malignancies. *Int J Radiat Oncol Biol Phys.* 2001;50(2):353-357.
5. Blom M, Lundeberg T. Long-term follow-up of patients treated with acupuncture for xerostomia and the influence of additional treatment. *Oral Dis.* 2000;6(1):15-24.
6. Rydholm M, Strang P. Acupuncture for patients in hospital-based home care suffering from xerostomia. *J Palliat Care.* 1999;15(4):20-23.
7. Blom M, Dawidson I, Angmar-Mansson B. The effect of acupuncture on salivary flow rates in patients with xerostomia. *Oral Surg Oral Med Oral Pathol.* 1992;73(3):293-298.
8. Blom M, Dawidson I, Fernberg JO, Johnson G, Angmar-Mansson B. Acupuncture treatment of patients with radiation-induced xerostomia. *Eur J Cancer B Oral Oncol.* 1996;32B(3):182-190.
9. Dawidson I, Angmar-Mansson B, Blom M, Theodorsson E, Lundeberg T. Sensory stimulation (acupuncture) increases the release of calcitonin gene-related peptide in the saliva of xerostomia sufferers. *Neuropeptides.* 1999;33(3):244-250.
10. Dawidson I, Angmar-Mansson B, Blom M, Theodorsson E, Lundeberg T. Sensory stimulation (acupuncture) increases the release of vasoactive intestinal polypeptide in the saliva of xerostomia sufferers. *Neuropeptides.* 1998;32(6):543-548.
11. Dawidson I, Angmar-Mansson B, Blom M, Theodorsson E, Lundeberg T. The influence of sensory stimulation (acupuncture) on the release of neuropeptides in the saliva of healthy subjects. *Life Sci.* 1998;63(8):659-674.
12. Johnstone PA, Niemtzow RC, Riffenburgh RH. Acupuncture for xerostomia: clinical update. *Cancer.* 2002;94(4):1151-1156.
13. Johnstone PA, Polston GR, Niemtzow RC, Martin PJ. Integration of acupuncture into the oncology clinic. *Palliat Med.* 2002;16(3):235-239.
14. Meng Z, Garcia MK, Hu C, et al. Randomized controlled trial of acupuncture for prevention of radiation-induced xerostomia among patients with nasopharyngeal carcinoma. *Cancer.* 2012;118(13):3337-3344.
15. Meng Z, Kay Garcia M, Hu C, et al. Sham-controlled, randomised, feasibility trial of acupuncture for prevention of radiation-induced xerostomia among patients with nasopharyngeal carcinoma. *Eur J Cancer.* 2012;48(11):1692-1699.
16. Helms JM. *Acupuncture energetics: a clinical approach for physicians.* Berkeley, Calif: Medical Acupuncture Publishers; 1997.
17. Park J, White A, Stevinson C, Ernst E, James M. Validating a new non-penetrating sham acupuncture device: two randomised controlled trials. *Acupunct Med.* 2002;20(4):168-174.
18. Park JJ. Developing and validating a sham acupuncture needle. *Acupunct Med.* 2009;27(3):93.

© 2019 Garcia MK et al. *JAMA Network Open.*

eTable 4. Acupuncture Expectation Score by Group

Institution	Mean (SD)*		
	TA	SA	SCC
Combined	N=118†	N=124	N=116
Baseline	9.03 (4.06)	9.22 (4.26)	9.41 (4.12)
Middle of treatment	8.62 (4.42)	7.82 (3.99)	
End of treatment	8.75 (4.29)	8.42 (4.20)	
Fudan	N=71	N=74	N=76
Baseline	9.29 (3.95)	9.71 (4.39)	9.46 (4.11)
Middle of treatment	8.45 (6.40)	8.10 (4.06)	
End of treatment	8.80 (4.23)	8.01 (4.15)	
MDACC	N=47	N=50	N=40
Baseline	8.64 (4.24)	8.52 (4.00)	9.33 (4.18)
Middle of treatment	8.85 (4.20)	7.43 (3.89)	
End of treatment	8.69 (4.43)	9.02 (4.23)	

*Raw means and standard deviations (SD); †N=baseline sample size + at least one follow-up at any timepoint.

© 2019 Garcia MK et al. *JAMA Network Open.*
