# Supplementary material for: Effect of True and Sham Acupuncture on Radiation-Induced Xerostomia Among Patients With Head and Neck Cancer: A Randomized Clinical Trial
Source: JAMA Netw Open. 2019 Dec 6;2(12):e1916910. doi: 10.1001/jamanetworkopen.2019.16910 (PMC6902763; doi:10.1001/jamanetworkopen.2019.16910)
Supplement: Supplement 3. — Data Sharing Statement [file jamanetwopen-2-e1916910-s003.pdf]

# Data Sharing Statement

Garcia. Effect of True and Sham Acupuncture on Radiation-Induced Xerostomia Among Patients With Head and Neck Cancer. *JAMA Netw Open*. Published December 06, 2019.
10.1001/jamanetworkopen.2019.16910

**Data**
**Data available:** Yes
**Data types:** Deidentified participant data
**How to access data:** lcohen@mdanderson.org
**When available:** With publication

**Supporting Documents**
**Document types:** None

**Additional Information**
**Who can access the data:** researchers whose proposed use of the data has been approved
**Types of analyses:** for any purpose
**Mechanisms of data availability:** after approval of a proposal and with a signed data access agreement
